# AAK1-mediated phosphorylation of PDLIM5 and Talin1 promotes focal adhesion disassembly to accelerate cell migration

**DOI:** 10.1038/s41467-026-72501-w

**Published:** 2026-05-04

**Authors:** Daniela Krocianova, Alexander D. Dagg, Rory A. Clayton, David Potesil, Veronika Fedorova, Adam Harmanec, Viktoria Benova, Veronika Bosakova, Jonathan G. G. Kaufman, Petra Martinkova, Miroslava Alblova, Bernard T. Kelly, Katerina Hanakova, Pavel Roudnicky, Stephanie J. Spielman, Jan Fric, Filip Sroubek, Josef Houser, Antoni G. Wrobel, Evzen Boura, David J. Owen, Zbynek Zdrahal, Zuzana Kadlecova

**Affiliations:** 1 Department of Histology and Embryology, Faculty of Medicine, Masaryk University, Brno, Czech Republic; 2 Cambridge Institute for Medical Research, University of Cambridge, Hills Road, Cambridge, UK; 3 CEITEC, Masaryk University, Brno, Czech Republic; 4 Czech Academy of Sciences, Institute of Information Theory and Automation, Prague, Czech Republic; 5 Faculty of Mathematics and Physics, Charles University, Prague, Czech Republic; 6 Institute of Organic Chemistry and Biochemistry of the Czech Academy of Sciences, Prague, Czech Republic; 7 International Clinical Research Center, St. Anne’s University Hospital in Brno, Brno, Czech Republic; 8 Centre of Molecular Structure, Institute of Biotechnology of the Czech Academy of Sciences, BIOCEV, Vestec, Czech Republic; 9 Childhood Cancer Data Lab, Alex’s Lemonade Stand Foundation, Bala Cynwyd, PA USA; 10 Institute of Hematology and Blood Transfusion, Prague, Czech Republic; 11 Core Facility Biomolecular Interactions and Crystallization, Masaryk University, Brno, Czech Republic; 12 Department of Biochemistry, University of Oxford, Oxford, UK; 13 Structural Biology of Disease Processes Laboratory, The Francis Crick Institute, London, UK

**Keywords:** Focal adhesion, Cell signalling, Endocytosis

## Abstract

AAK1 and BMP2K are serine/threonine kinases traditionally known for phosphorylating AP2 during clathrin-mediated endocytosis (CME), but their broader roles remained incompletely defined. Here, using motif-guided in silico, biochemical, and phosphoproteomic screens, we identify PDLIM5 and Talin1 as direct AAK1/BMP2K substrates. Despite high kinase-domain similarity, only AAK1 promotes cell migration and potentiates focal adhesion (FA) turnover. Live-cell imaging shows that AAK1 recruitment to FAs peaks as disassembly begins. The conserved AAK1 C-terminal PDZ-binding motif mediates direct, low-affinity binding to PDLIM5, providing a plausible mechanism for localized substrate access. Dynamic analyses of phospho-mimetic and phospho-null mutants support a model in which AAK1-dependent phosphorylation promotes timely release of PDLIM5 and Talin1 during FA disassembly. These findings reveal a kinase-driven contribution to FA turnover distinct from protease- and phosphatase-based mechanisms and suggest that functional divergence between AAK1 and BMP2K may provide a strategy to modulate cell migration with reduced impact on CME.

## Abstract (web summary)

Cell migration depends on controlled adhesion turnover, but its regulation remains unclear. Here the authors show that AAK1 promotes focal adhesion disassembly via PDLIM5 and Talin1 phosphorylation, enabling efficient cell migration.

## Introduction

Serine and threonine phosphorylation, the backbone of cellular signaling, is tightly regulated by protein kinases that must accurately recognize specific substrates among many similar protein sequences to ensure proper signal transduction and scaffolding. This precision maintains cellular homeostasis, whereas its dysregulation can lead to disease. Consequently, elucidating the regulatory mechanisms governing kinase activity and substrate specificity is critical for the development of targeted therapies^1,2^.

**Fig. 1:**
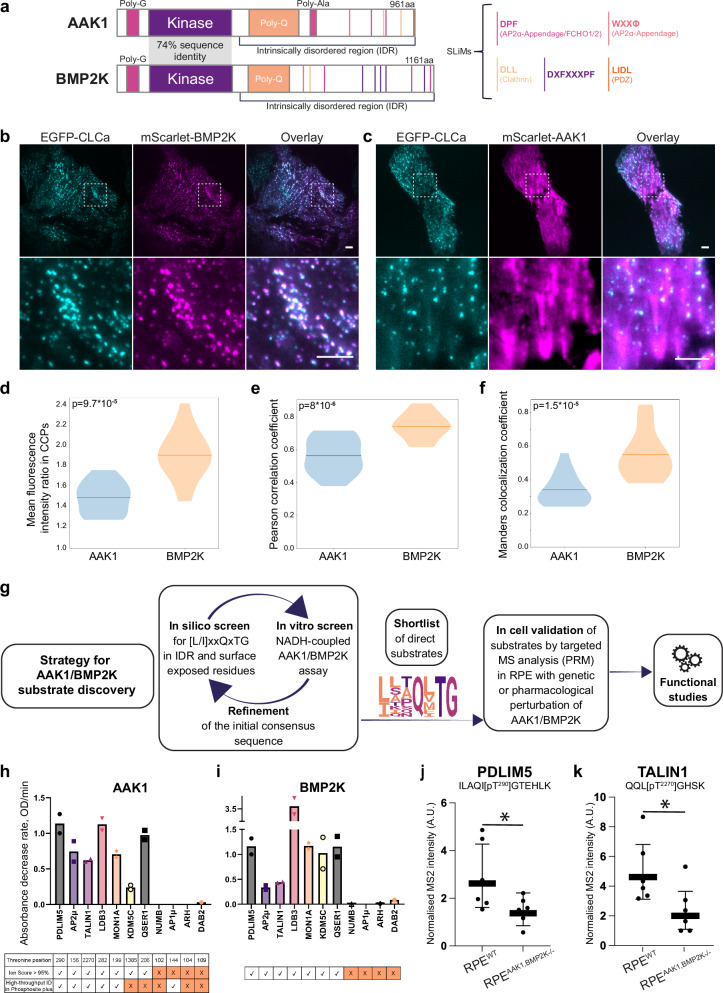
Discovery and validation of direct substrates of AAK1 and BMP2K in RPE Cells. **a** Schematic overview of domains from the canonical splice variants of AAK1 and BMP2K based on Uniprot and Ensembl database (accession numbers Q2M2I8-1/ NM_014911.5 and Q9NSY1-1, NM_198892.2). Cartoon adapted from^4^. Short linear motifs (SLiMs) and known interactors have been taken from the ELM database^83^. **b** TIRFM images showing distribution of mScarlet-BMP2K or **c** mScarlet-AAK1 in respect to eGFP-CLCa as a marker of CCPs in RPE cells, scale bar 5μm. Dashed white rectangles indicate regions magnified in the lower panels. **d–f** Quantitative analysis of AAK1 and BMP2K distribution in respect to eGFP-CLCa, comparing mean intensity ratios, Pearson correlation coefficients, and Manders colocalization coefficients (AAK1: *n* = 15 cells; BMP2K: *n* = 17 cells; three independent biological replicates, Welch’s two-tailed t-test, the horizontal line within each violin represents the mean value). **g** Experimental workflow used for discovering new physiological substrates of AAK1 and BMP2K. **h**, **i** Bar graphs showing the in vitro phosphorylation rates of candidate peptide substrates. Initial rates measured at 1 mM peptide concentration (X-axis) and normalized to the phosphorylation rate of PDLIM5. Data points represent two independent experiments. Annotations below the X-axis include localization probability scores from Mascot phosphosite analysis and references from the PhosphoSitePlus database. **j** Comparison of the abundance of the PDLIM5 phosphopeptide ILAQI[pT 290]GTEHLK in RPE^WT^ and RPE^AAK1,BMP2K-/-^, (*p* = 0.035, Welch’s two-tailed t-test, *n* = 6 independent biological replicates, error bar represents mean ± SD). **k** Comparison of the abundance of the Talin1 phosphopeptide QQL[pT 2270]GHSK in RPE^WT^ and RPE^AAK1,BMP2K-/-^ (*p* = 0.040, Welch’s two-tailed t-test, *n* = 6 independent biological replicates, error bar represents mean ± SD).

This is especially important for paralogous kinases, where one paralog may be effectively targeted therapeutically, while its closely related counterpart remains poorly understood^3^. A prime example is the Numb-associated kinase (NAK) family. Among its members, the paralogs AAK1 and BMP2K (also known as BIKE) share 74% sequence identity across their kinase domains (Fig. 1a)^4^. This high similarity yields nearly indistinguishable active sites, making it extremely challenging to design inhibitors that selectively target AAK1 over BMP2K and vice versa^5^. The broad-spectrum NAK inhibitors simultaneously targeting both AAK1 and BMP2K obscure their unique contributions and exaggerate perceived functional redundancy.

AAK1, however, has garnered considerable attention due to its potential in antiviral therapies^6,7^, neuropathic pain interventions^8,9^, and in enhancing the efficacy of T-cell–based cancer immunotherapies^10,11^. Moreover, AAK1 inhibition sensitizes melanoma cells to MAPK pathway inhibitors, highlighting its potential in overcoming treatment resistance in advanced cancers^12^. Despite this interest, the molecular mechanisms by which AAK1 exerts these diverse effects remain unclear, emphasizing the need to define its regulation and substrate repertoire. By contrast, BMP2K has remained comparatively overlooked, with only a handful of specialized functions (such as a role in erythroid differentiation) identified to date^13^.

Currently, the only well-established substrate shared by both AAK1 and BMP2K is AP2μ subunit (AP2μ) T156^14–17^, with surrounding residues matching the [L/I]XXQXTG phosphorylation motif described in yeast NAK kinases Ark1/Prk1^18,19^. We have shown that T156 phosphorylation facilitates recruitment of NECAP, SNX9, and dynamin, driving productive clathrin-coated pit (CCP) formation^20^. This has long supported the view that AAK1 and BMP2K are largely redundant mediators of endocytic trafficking in vertebrates. In addition to their presumed shared substrate preferences, both AAK1 and BMP2K possess an atypical activation segment - a β-strand followed by a C-terminal helix^4,21^. This structural feature confers constitutive activity, allowing the kinases to bypass the phosphorylation step required for activation in most kinases^22,23^. Might their regulation instead depend primarily on localization and recruitment by specific interaction partners, mediated via their long, intrinsically disordered C-terminal regions?

To address if and how these kinases achieve substrate and functional specificity, we performed systematic substrate screening coupled with targeted mass spectrometry (MS) approaches, testing both [L/I]XXQXTG and non-consensus phosphorylation motifs. Our data also reveal the regulatory mechanisms underlying the functional divergence between AAK1 and BMP2K. AAK1 preferentially localizes to mature FAs, and perturbing AAK1 activity reduces FA turnover and slows cell migration. These findings reveal a previously unknown contribution of AAK1 to FA dynamics and cell motility. They also challenge the assumption that AAK1 and BMP2K are redundant in endocytosis, showing instead that AAK1 has a specialized function not shared by BMP2K. Together, our data suggest that divergence between these paralogous kinases may lie primarily in when and where they act, rather than in big differences in intrinsic substrate preferences.

## Results

We first examined the localization patterns of AAK1 and BMP2K at the plasma membrane (PM) using live-cell total internal reflection fluorescence microscopy (TIRFM). To this end, we generated retinal pigment epithelial (RPE) cell lines where endogenous kinases were replaced by N-terminally mScarlet-tagged AAK1 (RPE^AAK1-rescue^) or BMP2K (RPE^BMP2K-rescue^) at approximately three-fold higher levels than their endogenous counterparts (Supplementary Fig. 1a, b). The distinct localization of AAK1 and BMP2K indicated that they may have divergent functional roles despite their sequence similarity (Fig. 1b, c). BMP2K exhibits prominent concentration within CCPs at the PM, as evidenced by a linear increase in fluorescence intensity alongside eGFP-Clathrin light chain A (eGFP-CLCa) levels (Fig. 1d–f). In contrast, AAK1 is broadly distributed across the PM, with 40% lower fluorescence intensity in CCPs in comparison to BMP2K. This difference suggests that AAK1 may participate in cellular processes beyond CME, prompting us to initiate a comprehensive screen to identify novel direct substrates of these kinases.

### In silico screening and in vitro assessment of candidate substrates

We used a combined workflow involving discovery and validation phases (Fig. 1g) to identify candidate substrates for AAK1 and BMP2K. First, in silico and in vitro screens were used to identify phosphorylation sites containing the [L/I]XXQXTG motif, proposed as the consensus motif based on studies of the yeast NAKs - Ark1 and Prk1^19^. We then validated the candidate substrates in RPE cell lysates using parallel reaction monitoring (PRM) coupled with liquid chromatography–mass spectrometry (LC-MS)^24^. Targeted PRM-LC-MS enabled us to quantify phosphopeptide abundances associated with AAK1 and BMP2K activity and refine the consensus motif.

An in silico screen identified 82 human proteins containing the [L/I]XXQXTG sequence with threonine in disordered or surface-exposed regions (Supplementary Data 1). Using an NADH-coupled kinase assay^25^, we tested synthetic peptides derived from these sequences for phosphorylation by the catalytic domains of both AAK1 and BMP2K. The well-characterized AP2μ peptide containing residue T156 (ITSQVTG) served as a positive control. (Fig. 1h, i; Supplementary Fig. 2). These experiments demonstrated that phosphorylation by AAK1 and BMP2K requires a threonine phosphoacceptor with hydrophobic residues at positions +1 and +5, while acidic residues are excluded, refining the consensus motif to [L/I]XXQϕTG (Fig. 1g, Supplementary Table 1, 2). This refined consensus sequence also explains why previously proposed substrates Dab2 T109, Arh T104, NUMB T102, and AP1μ T144^26^ showed no detectable phosphorylation in our in-vitro experiments (Fig. 1h, i; Supplementary Fig. 2; Supplementary Table 1, 2): their sequences either lack the critical recognition residues of the [L/I]XXQϕTG motif and/or contain inhibitory acidic residues (Supplementary Table 2). Based on robust in vitro phosphorylation at [L/I]XXQϕTG, we selected 11 peptides (Supplementary Table 3) from convergent membrane trafficking and cytoskeletal pathways for PRM analysis in RPE cells. The peptides were also chosen based on optimal LC-MS characteristics, including length and solubility.

### Validation of high-confidence hits in RPE cells

To translate our in vitro data into a cellular context, we next tested the presence of these candidate phosphopeptides in RPE lysates and quantified how their phosphorylation responds to combined AAK1 and BMP2K deletion (RPE^AAK1,BMP2K-/-^, Supplementary Fig. 1c). The use of double knock-out cells allowed us to capture phosphorylation driven by either kinase while mitigating potential paralogue redundancy. Targeted MS using PRM detected eight of the eleven pre-selected phosphopeptides in RPE cells (Supplementary Table 3). Phosphorylation of PDLIM5 T290 and Talin1 T2270 decreased reproducibly by more than 50% in RPE^AAK1,BMP2K-/-^ (Fig. 1j, k). In contrast, we did not detect statistically significant changes in phosphorylation for the other identified phosphopeptides INTS3 pT287, KDM5C pT1385, KDM5D pT1369, and MRPL44 pT287 (Supplementary Fig. 3). Together with the established AP2μ T156 phosphosite, these findings identify PDLIM5 and Talin1 as the principal cellular phosphotargets of AAK1 and/or BMP2K within our candidate set. Importantly, the total protein levels of PDLIM5 and Talin1 did not decrease (Supplementary Fig. 4), supporting the interpretation that the reduced PRM signals reflect decreased phosphorylation rather than reduced protein levels. Both proteins are mechanosensitive adhesome elements: PDLIM5 relocates to force-bearing actin stress fibers and FAs, whereas Talin1 acts as a tension sensor linking integrins to the cytoskeleton and coordinating downstream signaling^27–29^. The similar sensitivities of both PDLIM5 and Talin1 suggest a potential, yet undefined, role for AAK1 or BMP2K within the adhesome, extending beyond their known role in endocytosis.

### Global proteome and phosphoproteome profiling reveal AAK1/BMP2K control of the adhesome

**Fig. 2:**
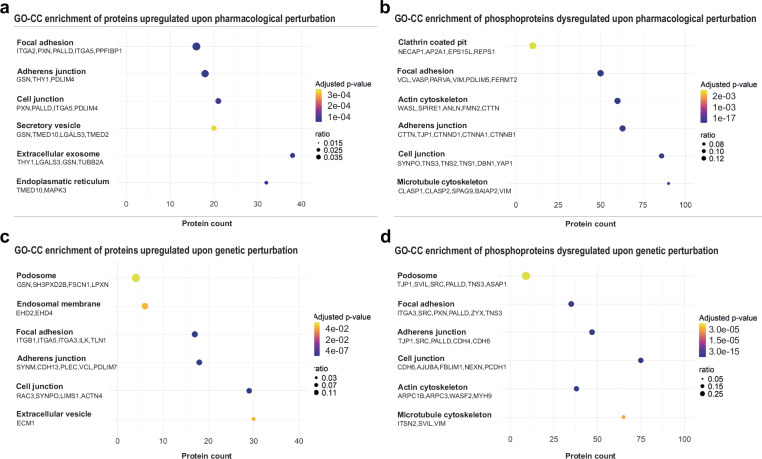
GO enrichment analysis reveals AAK1/BMP2K roles in maintaining adhesome and cytoskeletal component abundance. **a** Cellular components enriched for proteins upregulated in RPE^WT^LP cells following pharmacological inhibition (log2 fold change > 0.3, adjusted *p*-value < 0.05). **b** Cellular components enriched for proteins with dysregulated phosphorylation in RPE^WT^LP cells upon pharmacological inhibition (log2 fold change > 0.5, adjusted *p*-value < 0.05). **c** Cellular components enriched for proteins upregulated in RPE^AAK1,BMP2K-/-^ cells (log2 fold change > 0.5, adjusted *p*-value < 0.05). **d** Cellular components enriched for proteins with dysregulated phosphorylation in RPE^AAK1,BMP2K-/-^ cells (log2 fold change > 1, adjusted *p*-value < 0.05). Dot size represents the proportion of proteins in each category relative to the total analyzed, with color indicating the adjusted p-values for significance. Statistical analysis was performed using LIMMA with Benjamini-Hochberg correction.

To assess the global impact of AAK1 and BMP2K on adhesion networks, we analyzed the proteome and phosphoproteome of RPE cells under two orthogonal perturbations: pharmacological inhibition (RPE^WT^LP) and genetic double knockout (RPE^AAK1,BMP2K-/-^). Treatment of RPE cells with 10 μM LP-935509 for 6 h led to an 80% reduction in AP2μ T156 phosphorylation, as demonstrated in our previous work^20^ and reproduced in the next section (see below). LP-935509 potently inhibits both AAK1 and BMP2K (AAK1 IC₅₀ = 3.3 nM, BMP2K IC₅₀ 14 nM)^9^. In both conditions, we identified an upregulation of FA and cell junction components upon perturbation, suggesting a targeted regulatory mechanism rather than widespread proteome changes (Fig. 2, Supplementary Fig. 5, Supplementary Data 2, Data 3). Furthermore, comparing acute pharmacological inhibition with chronic genetic loss of AAK1/BMP2K reveals that the overlap is substantially more robust at the phosphoproteome level than at the proteome level. We identify ~364 phosphoproteins significantly dysregulated in both conditions ( ≈ 22 % intersection of the union), whereas only 23 proteins are commonly upregulated at the total protein level ( ≈ 4 % intersection; Supplementary Fig. 6a). This stronger convergence at the phosphoprotein level is consistent with a kinase-centered mechanism and indicates that both perturbations affect a shared set of phosphorylation-regulated proteins. Moreover, the intersecting dysregulated phosphoproteins are enriched for FA/cell–matrix junction and actin cytoskeleton components (Supplementary Fig. 6b). Consistent with this, GO Cellular Component (GO-CC) enrichment shows substantial pathway-level convergence between perturbations: 10 shared GO-CC terms ( ~ 20% of 49 total) for the upregulated proteome and 49 shared GO-CC terms ( ~ 47% of 104 total) for the dysregulated phosphoproteome (Supplementary Fig. 6c).

To investigate whether AAK1 and BMP2K phosphorylate non-consensus sequences, we analyzed phosphosites that were significantly downregulated in RPE^AAK1,BMP2K-/-^ cells. From the dataset (Supplementary Data 2), we identified threonine phosphosites followed by glycine that were most affected by the deletion of both kinases, including ARRB1 T410, TROT T157, IQCE T596, and FMN2 T105 (Supplementary Table 4). However, our in vitro kinase assays demonstrated that AAK1 and BMP2K did not phosphorylate any of these sites (Supplementary Fig. 2). These findings confirm that vertebrate AAK1 and BMP2K specifically target the [L/I]XXQϕTG motif (Supplementary Fig. 2, Supplementary Table 2). Furthermore, our global phosphoproteome profiling of RPE cells failed to detect phosphorylation of the previously proposed substrates Dab2 T109, Arh T104, or NUMB T102. This conclusion is supported by both our in vitro kinase assays and the absence of phosphorylation evidence in high-throughput databases, such as PhosphoSitePlus (Fig. 1h, i), which suggests that Dab2 T109, Arh T104, and NUMB T102 are unlikely to be physiological targets of AAK1 or BMP2K.

Together, these data indicate that AAK1/BMP2K-dependent phosphorylation shapes the phosphoproteome of adhesome components. In addition, the striking distribution of AAK1 at the PM, contrasting with BMP2K enrichment in CCPs, led us to hypothesize that AAK1 has a dominant role in FA signaling, which we test in the following sections.

### AAK1 accelerates cell migration by promoting FA turnover, a role independent of endocytosis

**Fig. 3:**
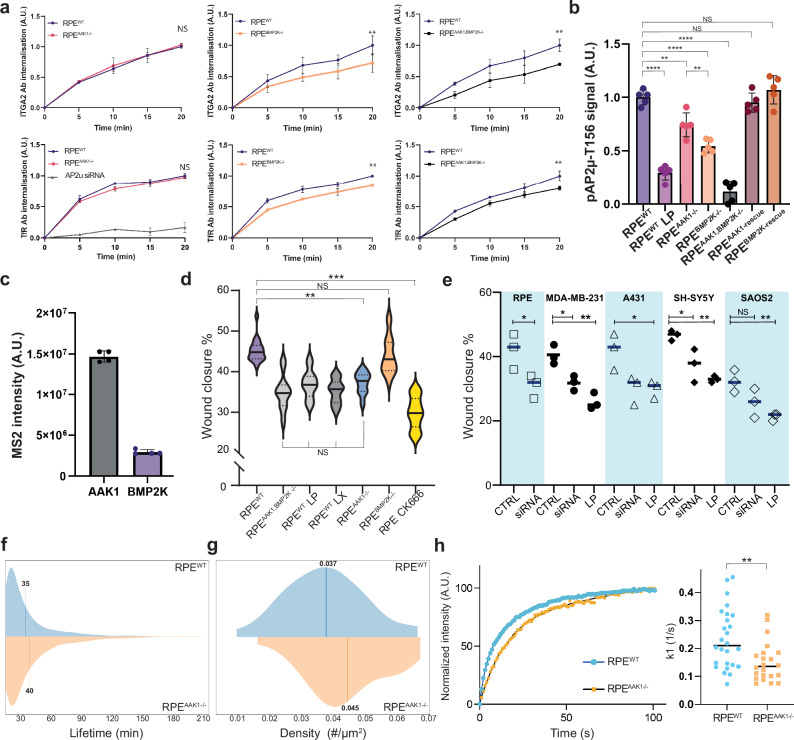
AAK1 drives cell migration via non-endocytic mechanisms. **a** Time course plots displaying the internalization rates of integrin α2 and TfR in RPE^WT^, RPE^AAK1-/-^, RPE^BMP2K-/-^ and RPE^AAK1,BMP2K-/-^, *n* = 3 biological replicates, error bars represent mean ± SD. Positive control: AP2μ knockdown ( ~ 80%) decreased TfR uptake by ~85%. Statistical significance is indicated as NS *p* > 0.05, **p < 0.01. **b** Bar graph representing the in-cell ELISA experiments to determine phosphorylation levels of AP2μ at T156 in RPE^WT^, RPE^WT^LP (*p* < 0.0001), RPE^AAK1-/-^ (*p* = 0.0012), RPE^BMP2K-/-^ (*p* < 0.0001) and RPE^AAK1,BMP2K-/-^ (*p* < 0.0001), *n* = 5 independent experiments, error bars represent mean ± SD. Statistical significance was assessed using one-way ANOVA followed by Tukey’s post hoc analysis. **c** Bar graph showing MS2 intensity for AAK1 and BMP2K as a sum of intensities of their five most intense proteotypic peptides to compare the abundance of AAK1 in comparison to BMP2K in RPE cells, *n* = 4 biological replicates, error bars represent mean ± SD. **d** Violin plot showing wound closure percentage as a proxy for cell migration in RPE^WT^, RPE^AAK1-/-^, RPE^BMP2K-/-^, RPE^AAK1,BMP2K-/-^ and RPE cells treated with LP-935509 and LX-9211, or CK666 (Arp2/3 complex inhibitor, positive control). Medians and quartiles are indicated by the horizontal line. Statistical analysis was performed from n = three independent biological replicates (24 wells per condition), using one-way ANOVA followed by Tukey’s post hoc test. Statistical significance is indicated as NS *p* > 0.05, **p < 0.01, ***p < 0.001. **e** Wound-healing assays in RPE, MDA-MB-231, A431, SH-SY5Y, and SAOS2 cells following AAK1 knockdown (siRNA) or AAK1 and BMP2K inhibition using LP-935509. Statistical analysis was performed from n = three independent biological replicates (24 wells per condition), using one-way ANOVA followed by Tukey’s post hoc test, horizontal bars indicate mean values. Statistical significance is indicated as NS = *p* > 0.05, *p < 0.05, **p < 0.01. **f** Ridgeline plot depicting distribution of FA lifetimes (assessed via mNeonGreen-paxillin) in RPE^WT^ and RPE^AAK1-/-^ cells, mean is indicated as a vertical line; *p* = 0.00002; RPE^WT^: *n* = 30 cells, 2808 segmented FAs; RPE^AAK1-/-^: *n* = 30 cells, 4554 segmented FAs; Mann–Whitney U test was used for statistical analysis. **g** Ridgeline plot depicting distribution of FA density, mean is indicated as a vertical line; *p* = 0.02; RPE^WT^: *n* = 30 cells, 2808 segmented FAs; RPE^AAK1-/-^: *n* = 30 cells, 4554 segmented FAs; Welch’s two-tailed t-test. **h** FRAP analysis of mNeonGreen–paxillin dynamics at FAs in RPE^WT^ and RPE^AAK1-/-^. Left: representative recovery curves, right: quantification of the fast recovery rate (k1). RPE^AAK1-/-^: *n* = 27 cells, RPE^WT^: *n* = 23 cells, *p* = 0.022, Welch’s two-tailed t-test.

Building on our localization and proteomic findings, we dissected the distinct roles of AAK1 and BMP2K by systematically comparing their contributions to CME versus FA dynamics. BMP2K deletion (RPE^BMP2K-/-^), or deletion of both kinases, reduced internalization of transferrin receptor (TfR) and integrin α2^30^. AP2μ knock-down served as a positive control and strongly reduced TfR internalization (Fig. 3a, Supplementary Fig. 1d). BMP2K deletion also sharply reduced phosphorylation of the T156 in AP2μ, a key regulator of CME rates (Fig. 3b). Surprisingly, despite AAK1 being fivefold more abundant than BMP2K in RPE cells (Fig. 3c), its deletion (RPE^AAK1-/-^) had no measurable effect on receptor internalization and reduced AP2μ phosphorylation only by 20%. This difference likely arises from the predominant localization of BMP2K at CCPs, compared to the weaker recruitment of AAK1 (Fig. 1b, f). Together, these data indicate that BMP2K, rather than AAK1, is the primary NAK regulating CME in RPE cells. However, identification of the adhesome proteins PDLIM5 and Talin1 as AAK1/BMP2K-responsive substrates, together with broader PM distribution of AAK1, prompted us to test whether AAK1 also contributes to adhesion-dependent processes during cell migration. To investigate this, we performed wound-healing and FA turnover assays. AAK1 deletion or siRNA-mediated knockdown reproducibly impaired RPE migration by ~25% (Fig. 3d, e and Supplementary Fig. 1h), whereas BMP2K loss had no measurable effect, revealing a specific and non-redundant role for AAK1 in cell motility. Importantly, the phenotype was kinase-dependent: two chemically unrelated AAK1/BMP2K targeting inhibitors - LP-935509 (an acyclic pyrazolopyrimidine) and LX-9211/BMS-986176 (a distinct bi(hetero)aryl ether chemotype reported to be highly selective across the kinome) similarly slowed migration (Fig. 3d). Using both compounds provides orthogonal pharmacological validation and reduces the likelihood that the migration defect reflects compound-specific off-target effects. To assess generalizability, we extended the analysis to additional cell lines with high AAK1 expression and efficient siRNA-mediated knockdown (Fig. 3e, Supplementary Fig. 1e). In both SH-SY5Y neuroblastoma cells and MDA-MB-231 breast carcinoma cells (epithelial origin but exhibiting a poorly polarized, mesenchymal-like phenotype^31,32^), AAK1 knockdown likewise slowed migration. Together, these data indicate that AAK1-dependent, kinase-driven motility extends across multiple cellular contexts, including cell lines lacking classical epithelial junctional organization.

Consistent with the migration defect, AAK1 loss altered FA dynamics. In RPE^AAK1-/-^, FA density increased and individual FAs persisted longer (Fig. 3f, g), implying impaired FA turnover^33,34^. To quantify this effect, we monitored mNeonGreen-paxillin recovery after photobleaching (FRAP) (Fig. 3h, Supplementary Fig. 1f)^35,36^. Paxillin is known to exhibit relatively slow FRAP recovery with a substantial stably bound fraction at FAs, making it a sensitive readout of changes in adhesion stability^35–37^. mNeonGreen-Paxillin expressed in RPE^AAK1-/-^ cells showed a pronounced reduction in the recovery rate (k₁), mirroring the slower paxillin exchange reported for overly stabilized FAs in other systems^38^. Because rapid paxillin turnover is essential for efficient cell migration, these data position AAK1 as a positive regulator of FA disassembly and motility. This prompted us to examine downstream effectors, beginning with the mechanosensor PDLIM5 and its phosphorylation at T290.

### Identification of PDLIM5 as an AAK1 substrate involved in FA turnover

**Fig. 4:**
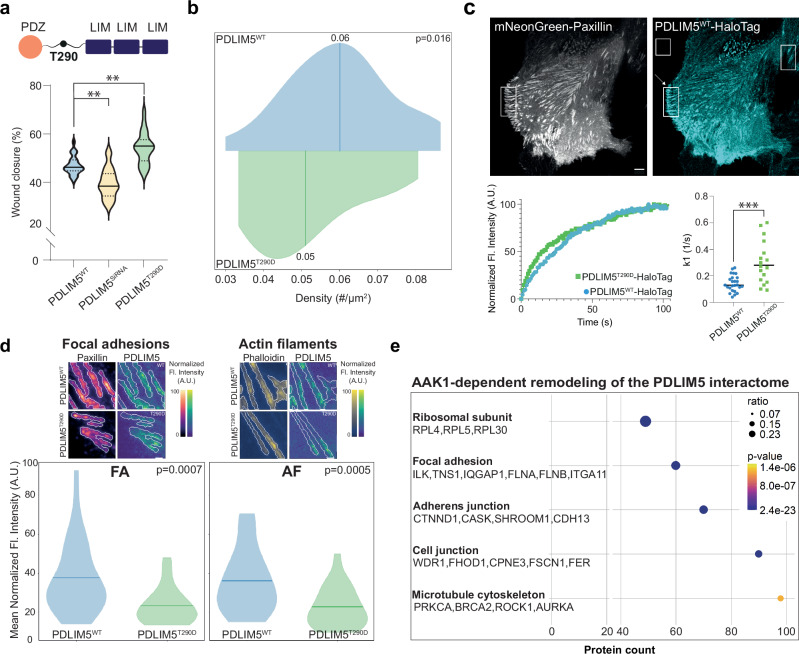
Phosphorylation of PDLIM5 at T290 accelerates FA dynamics and cell migration by switching PDLIM5 localization. **a** Top: Illustration of PDLIM5 domain organization and position of the T290 phosphosite. Bottom: Wound-healing assay, **p < 0.01, one-way ANOVA with Tukey’s post hoc test. **b** Ridgeline plot of FA densities (assessed via mNeonGreen-paxillin) in PDLIM5^T290D^ cells compared to PDLIM5^WT^. Mean is indicated as a vertical line. RPE PDLIM5^WT^: *n* = 26 cells, 3910 segmented FAs; RPE PDLIM5^T290D^: *n* = 21 cells, 2408 segmented FAs; Welch’s two-tailed t-test. **c** FRAP analysis of PDLIM5 at FAs. Representative confocal microscopy images of a cell co-expressing mNeonGreen-paxillin and PDLIM5-HaloTag with highlighted area of PDLIM5 colocalizing with paxillin (white rectangle) used for photobleaching, reference region (top right), and background area (top left); scale bar 5 μm. Representative example of fluorescence recovery curves for PDLIM5^WT^-HaloTag (blue) and PDLIM5^T290D^–HaloTag (green) and the recovery rates (k_1_) (*n* = 30 cells, *p* < 0.001). **d** Top: Representative TIRFM images of PDLIM5^WT^ and PDLIM5^T290D^ at FAs (mNeongreen-paxillin) and along actin filaments (phalloidin). Normalized fluorescence intensity is colored and indicated by the bars to the right of the images; scale bar 1 μm. Bottom: Violin plots with *p*-values of normalized fluorescence intensity of PDLIM5 at FAs and actin filaments. PDLIM5^WT^: *n* = 26 cells, 3910 segmented FAs, 2523 segmented actin filaments; PDLIM5^T290D^: *n* = 21 cells, 2408 segmented FAs, 1588 segmented actin filaments; Welch’s two-tailed t-test; the horizontal line within each violin represents the mean value. e Dot plot depicting AAK1-dependent remodeling of the PDLIM5 interactome. GO analysis includes only proteins with log2 fold change > 0.3 and *p*-value < 0.05. Statistical analysis was performed using LIMMA with Benjamini-Hochberg correction.

PDLIM5 is a PDZ-LIM domain protein (Fig. 4a) classified as an FA component^28,39^ with both structural and regulatory roles in the cytoskeleton. Its N-terminal PSD-95/Dlg/ZO-1 (PDZ) domain binds to α-actinin, tethering and stabilizing actin filament bundles. In addition, PDLIM5 serves as a signaling scaffold by assembling protein complexes with various kinases (e.g., protein kinase C and protein kinase D1)^40^ and is phosphorylated by AMPK^41^. The triad of LIN-11/Isl-1/MEC-3 (LIM) domains at its C-terminus is predicted to enable PDLIM5 to sense mechanical stress: they bind directly to tensed F-actin filaments, a conserved property of LIM proteins, and thereby contribute to force-dependent adhesion remodeling^42–44^. PDLIM5 knockdown in RPE cells ( ~ 90% efficiency, Supplementary Fig. 1e) caused a ~ 16% reduction in migration speed (Fig. 4a), confirming involvement of endogenous PDLIM5 in RPE migration. Reintroducing siRNA-resistant PDLIM5^WT^ and PDLIM5^T290D^ constructs at equal expression levels (Supplementary Fig. 1e) revealed that the phosphomimetic PDLIM5^T290D^ produced an opposite phenotype to AAK1 deletion. It increased migration speed by ~15% and decreased FA density by ~17% (Fig. 4a, b). Live-cell FRAP revealed accelerated exchange kinetics at FAs: PDLIM5^T290D^ recovered ~2-fold faster than PDLIM5^WT^ (Fig. 4c). Because the recovery plateau (mobile/immobile fraction) was unchanged (Supplementary Fig. 7a), this is most consistent with an increased apparent k_off_ (shorter residence time) together with a commensurate increase in effective k_on_, preserving the steady-state partitioning between kinetic pools^35,37^.

TIRFM further supported the shorter residence-time model by showing substantially reduced localization of PDLIM5^T290D^ at FAs and along actin filaments compared to PDLIM5^WT^ (Fig. 4d). This suggests that phosphorylation at T290 disrupts the multivalent interactions required for stable adhesome binding, shifting PDLIM5 from a more persistently bound state toward a more dynamic one, which may contribute to FA turnover and cytoskeletal remodeling. Phosphorylation at T290 could plausibly alter PDLIM5 conformation or weaken key intra- or intermolecular contacts, which in turn may reduce its association with F-actin and/or α-actinin–integrin–linked adhesion complexes. Consistent with this hypothesis, the composition of the PDLIM5 interactome is AAK1 dependent. In RPE^WT^ cells, PDLIM5 shows reduced association with adhesion scaffold and mechanosensing proteins ILK, tensin-1, IQGAP1, filamins A and B compared with RPE^AAK1−/−^ cells (Fig. 4e).

This is consistent with AAK1-dependent phosphorylation weakening the association of PDLIM5 with these interactors. Together, the migration and FA-density phenotypes, the accelerated FRAP exchange of the T290D phosphomimetic mutant, the reduced FA/actin-filament association, and the AAK1-dependent shift in the PDLIM5 interactome all point to a common mechanism. AAK1-dependent phosphorylation appears to bias PDLIM5 toward a more exchangeable state, thereby promoting timely FA turnover.

### Context-dependent recruitment of AAK1 to FAs drives substrate targeting

**Fig. 5:**
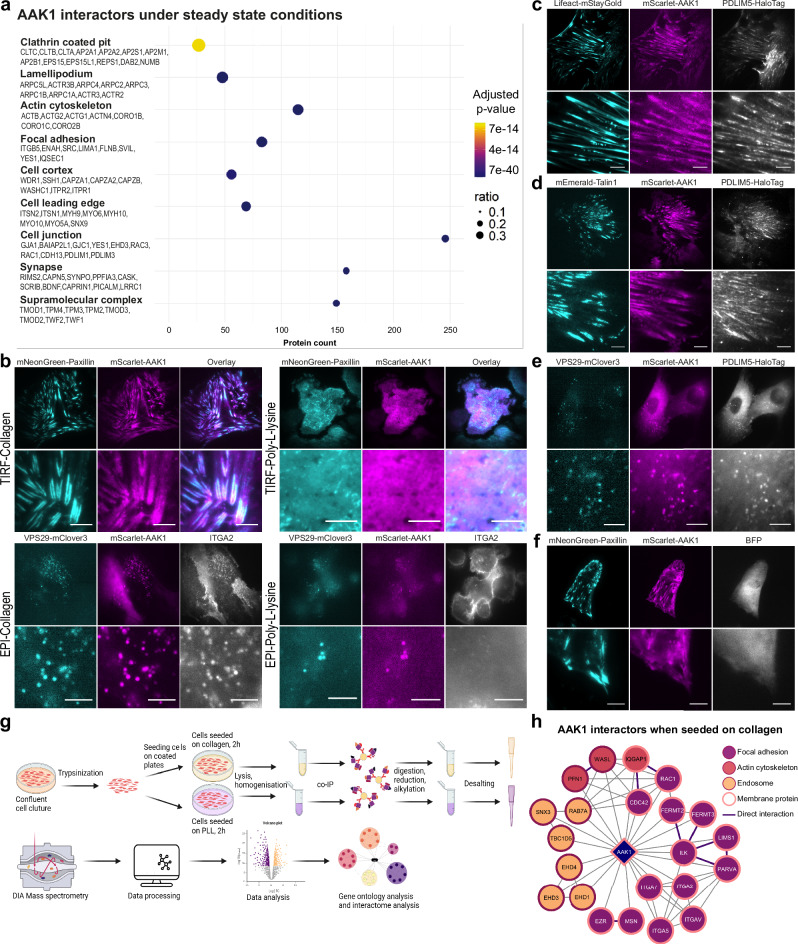
AAK1 localization and interaction network is dependent on ECM. **a** Dot plot visualizing the top enriched GO-CC terms for proteins co-immunoprecipitating with endogenous AAK1 under steady-state conditions. Dot size indicates the fraction of AAK1 interactors out of the total proteins annotated to each GO term, color indicates the adjusted *p*-value. Analysis was restricted to proteins with a log2 fold change > 1 and an adjusted *p*-value < 0.05. Statistical analysis was performed using LIMMA with Benjamini-Hochberg correction. **b** Live-cell imaging of mScarlet–AAK1 in RPE cells replated for 2 h on collagen I (integrin-engaging) or PLL (integrin-independent). Top: TIRFM showing AAK1 distribution relative to the FA marker mNeonGreen–paxillin; collagen promotes AAK1 enrichment at mature paxillin-positive FAs, whereas AAK1 remains diffuse on PLL. Bottom: Epifluorescence imaging showing AAK1 relative to VPS29–mClover3 (endosomes) and integrin α2 (ITGA2). **c** Colocalization of AAK1 with actin filaments (Lifeact) and PDLIM5 in collagen-seeded cells. **d** Colocalization of AAK1 with mEmerald-Talin1 and PDLIM5 in collagen-seeded cells. **e** Epifluorescence microscopy of mScarlet-AAK1 and its association with VPS29-positive and PDLIM5 positive endosomes in collagen-seeded cells. **f** Cytosolic BFP control imaged under the same TIRFM conditions as in panel B shows a homogeneous distribution, arguing that the FA associated AAK1 pattern is not due to uneven cell attachment or TIRFM illumination. **b–f** n = three independent experiments. **g** Scheme of the experimental workflow for co-IP MS and data analysis to compare context dependent AAK1-interactome. Created in BioRender. Kročianová, D. (2026) https://BioRender.com/8vb09uy. **h** GO analysis with STRING-based network visualization of proteins significantly enriched in the AAK1 interactome in collagen-seeded cells compared to PLL-seeded cells, highlighting functionally grouped components and edges indicate known interactions. Proteins with a *p*-value ≤ 0.05 and logFC > 0.3 were used for the DAVID GO analysis.

Having identified PDLIM5 and Talin1 as AAK1-responsive substrates, we next asked how AAK1 selectively targets substrates in cells. The atypical activation segment of AAK1 confers constitutive catalytic competence, suggesting that specificity is likely governed by context-dependent recruitment and local phosphorylation substrate recognition. Co-immunoprecipitation (co-IP) of endogenous AAK1 from collagen-adherent RPE cells under steady-state conditions recovered multiple FA components. Among these, α-actinin-4 was the most enriched (Supplementary Data 2). This protein is known to interact directly with PDLIM5, integrins, and the actin cytoskeleton, facilitating force transmission and FA maturation^45–48^ (Fig. 5a). PDLIM5 and Talin1 were detected in the AAK1 co-IP/MS datasets (Supplementary Data 2) but are not displayed in Fig. 5h because they did not pass the enrichment cutoffs used for the GO/STRING network (*p* ≤ 0.05 and log2 fold change > 0.3), as expected given that kinase–substrate interactions can be weak, transient and sub-stoichiometric. Because co-IP/MS is performed after lysis, weak interactions may be disrupted, and post-lysis associations may form, so we use these data to define context-dependent recruitment rather than to infer direct substrate binding. We therefore complemented co-IP/MS with live-cell TIRFM under defined adhesion conditions. RPE cells were replated for 2 h on collagen (integrin-engaging) or poly‑L‑lysine (PLL; integrin‑independent electrostatic attachment) to assess the contribution of integrin-mediated adhesion to AAK1 recruitment (Supplementary Movie 1)^49^. The 2-hour time window was selected to capture dynamic recruitment events, distinct from steady-state cellular conditions. Collagen-induced localization of AAK1 into large, mature paxillin-positive FAs that also contained integrin α2, PDLIM5 and Talin1 (Fig. 5b–d, Supplementary Fig. 8a). Notably, AAK1 was absent from nascent or short-lived adhesions. Moreover, we observed that VPS29-positive endosomes containing the key collagen receptor integrin α2 displayed substantial AAK1 localization, accompanied by PDLIM5 enrichment (Fig. 5e). In contrast to these observations, in cells seeded on PLL, AAK1 remained diffusely distributed at the PM (Fig. 5b).

Additional negative control experiments using transferrin-coated coverslips^49,50^, confirmed that AAK1 distribution remains uniform at the PM in absence of FA formation (Supplementary Fig. 8b). To further demonstrate that AAK1 FA localization is ECM-dependent and not merely an artifact of the mScarlet tag, labeling or uneven TIRFM illumination, we used the following negative controls: cytosolic BFP (Fig. 5f), PM–specific wheat germ agglutinin (Supplementary Fig. 8c), and a PtdIns^4,5^P2-specific pleckstrin homology domain probe (Supplementary Fig. 8d). Together, these controls rule out uneven cell attachment, TIRFM illumination bias, and tag mislocalization, confirming that AAK1 co-localization with mature FAs is a specific, ECM-dependent phenomenon.

To map the molecular basis of AAK1 dynamic adhesion recruitment, we directly compared the interactome of endogenous AAK1 after 2 h on collagen versus PLL, reproducing the conditions used in our TIRFM imaging (Fig. 5g, h). The AAK1 interactome showed a significant enrichment of integrins on collagen, specifically integrin α2, α5, αV, and α7. Notably, integrin α2 overall expression did not increase in collagen-plated cells (Supplementary Fig. 8e), indicating that its elevated presence in the AAK1 interactome is due to selective recruitment by AAK1 rather than higher integrin expression. The collagen-specific AAK1 interactome also included mechanotransduction ILK–PINCH–Parvin complex^51^ that facilitates the relay of force-dependent signals to stabilize integrin-ECM adhesion. Thus, AAK1 is specifically recruited to mature, tension-bearing FAs and thereby positioned to efficiently phosphorylate substrates PDLIM5 and Talin1.

### AAK1 peak recruitment marks the onset of FA disassembly

**Fig. 6:**
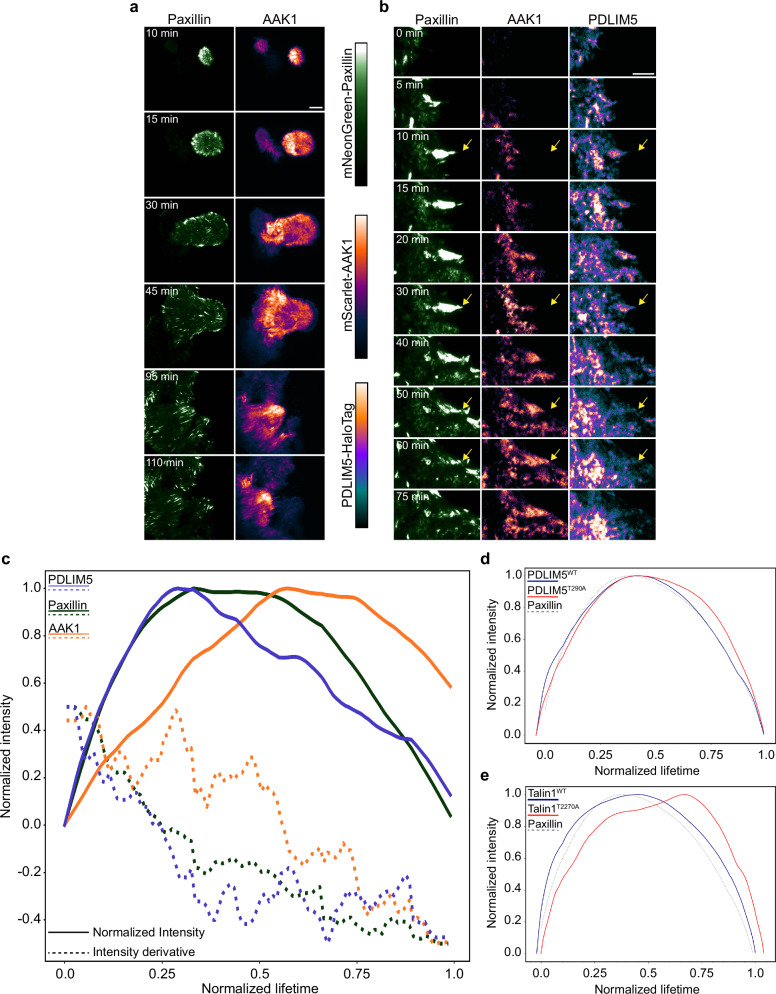
AAK1 recruitment peaks during FA disassembly. **a** Snapshots from live-cell TIRFM showing the dynamic redistribution of AAK1 at the PM in RPE cells upon attachment to collagen-coated surfaces, scale bar 10 μm. Cells co-express mNeonGreen-paxillin, mScarlet-AAK1, and PDLIM5-HaloTag. **b** Magnified view comparing the spatio-temporal hierarchy of recruitment of AAK1 and PDLIM5 to a FA (snapshots from movie presented in panel A). Yellow arrows highlight distinct stages of FA turnover, assembly, maturation, and disassembly, along with the comparative localization of each protein during these phases, scale bar 5 μm. **c** Lifetime-aligned intensity traces and recruitment-rate profiles for paxillin (green), PDLIM5 (blue), and AAK1 (orange) during FA turnover. Solid lines show the mean normalized intensity profiles across the FA lifetime, quantified from 823 FAs imaged by live-cell TIRFM. Dotted lines show the first derivatives of the intensity curves, reflecting the rates of recruitment or dissociation over time. **d**, **e** Lifetime-aligned mean intensity profiles from live-cell TIRFM comparing wild-type and phospho-null mutants at FAs. **d** PDLIM5^WT^ (blue) and PDLIM5^T290A^ (red) traces are plotted together with paxillin (gray dashed) as the FA reference. **e** Talin1^WT^ (blue) and Talin1^T2270A^ (red) traces are plotted together with paxillin (gray dashed). The x-axis represents normalized FA lifetime(0–1) using at least 1500 FAs per condition, and the y-axis shows normalized fluorescence intensity.

**Fig. 7:**
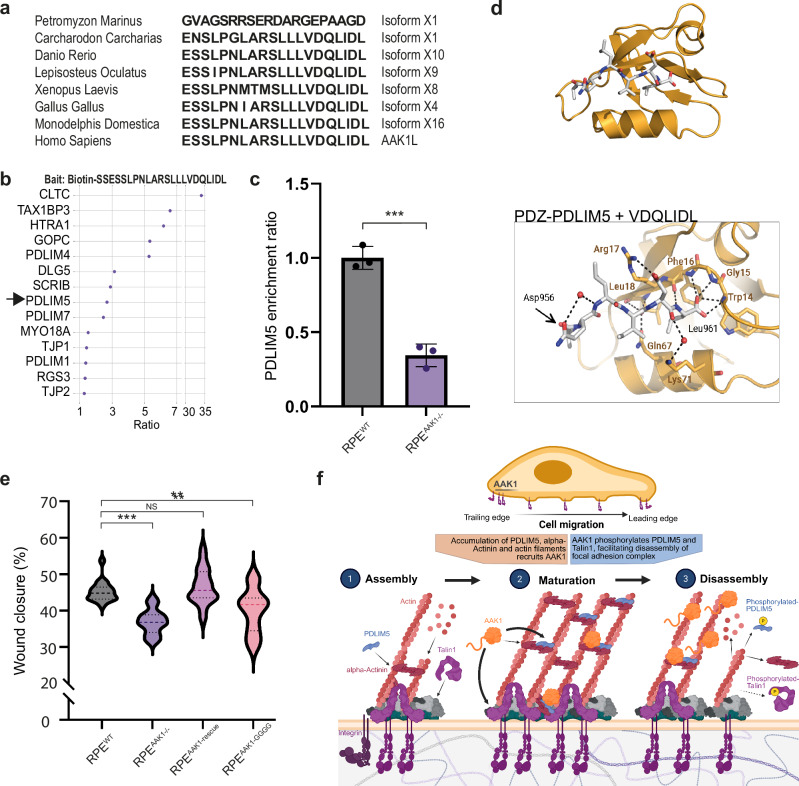
AAK1 interacts with PDLIM5 via LIDL motif and its role in cell migration. **a** Sequence alignment of AAK1 extreme C-terminus across various species, highlighting its evolutionary conservation in jawed vertebrates. **b** Dot plot representing the enrichment of clathrin and PDZ domain-containing proteins in an MS-based pull-down assay using AAK1 C-terminal peptide SSESSLPNLARSLLLVDQLIDL as a bait. **c** Quantification of PDLIM5 enrichment ratios from endogenous AAK1 co-IP in RPE^WT^ versus negative control RPE^AAK1-/-^, *p* = 0.0005, *n* = three biological replicates, error bar represents mean ± SD. **d** Front view of the crystal structure 9F6S solved here (1.0 Å resolution) depicting PDZ-PDLIM5 (gold) and a stick representation of the bound AAK1 derived VDQLIDL peptide (gray sticks). Bottom panel shows a detailed view of the interaction. **e** Violin plots representing wound closure percentages in RPE^WT^, RPE^AAK1-/-^, AAK1 RPE^AAK1-rescue^, and AAK1 C-terminal GGGG mutant (RPE^AAK1-GGGG^). Statistical analysis was performed from n = three independent biological replicates (24 wells per condition), using one-way ANOVA followed by Tukey’s post hoc test. Medians and quartiles are indicated by the horizontal line. Statistical significance is indicated as NS *p* > 0.05, **p < 0.01, ***p < 0.001. **f** The schematic depicts our model spatiotemporal role of AAK1 in FA dynamics^1^: scaffolding phase^2^, localized recruitment of AAK1^3^, network destabilization. Created in BioRender. Kročianová, D. (2026) https://BioRender.com/4no8tq8.

Having established the functional hierarchy in which AAK1 phosphorylates PDLIM5 to regulate its localization and interactions at FAs (Fig. 4), we next aimed to define the temporal hierarchy of these events and relate them to the role of AAK1 in promoting FA turnover. Live-cell TIRFM revealed that immediately after cell attachment to collagen, AAK1 redistributes from diffuse presence across the PM to FAs at both the leading and trailing edges of migrating cells (Fig. 6a, b and Supplementary Movie 2). Quantitative analysis of the fluorescence intensity profiles for hundreds of FAs shows that PDLIM5 intensity peaks at the point of maximum FA expansion. (Fig. 6b, c, Supplementary Movie 3), reminiscent of force-sensitive α-actinin recruitment to FAs^52^. We overlaid the average intensity traces with their first derivatives, depicted as dotted lines indicating recruitment rates (Fig. 6c). This analysis revealed that the maximum recruitment rate of AAK1 coincides precisely with the peak intensity of PDLIM5 (Fig. 6c). This temporal relationship suggests a potential feedback loop in which PDLIM5 facilitates AAK1 localization to FAs, a hypothesis directly tested in the next section (Fig. 7). Such context-specific recruitment positions AAK1 to phosphorylate its adhesome substrates, PDLIM5 and Talin1, while excluding AP2μ.

The highest intensity of AAK1, used as a proxy for its maximum local abundance at FAs, aligns with the end of the paxillin intensity plateau, marking the transition to rapid FA disassembly (Fig. 6c). As AAK1 levels rise locally and drive PDLIM5 phosphorylation at T290, the interaction of PDLIM5 with FAs and actin filaments shifts from associative to dissociative, ultimately accelerating cell migration. This temporal coordination correlates with accelerated FA disassembly mediated by AAK1-dependent phosphorylation of PDLIM5. To functionally connect this late AAK1 enrichment at the onset of FA disassembly with substrate dynamics during turnover, we repeated the same lifetime-aligned analysis using phospho-null mutants of both substrates (PDLIM5^T290A^ and Talin1^T2270A^; Fig. 6d, e). Both substrates showed delayed loss during the disassembly phase, consistent with phosphorylation promoting timely disengagement. Talin1^T2270A^ showed a pronounced late-phase persistence including a delayed time to fall below 80% of peak intensity (from 71% of normalized FA lifetime in Talin1^WT^ to 85% in the Talin1^T2270A^) and a marked increase in end-of-lifetime residual signal (from ~1.4% of peak in Talin1^WT^ to ~22.7% of peak in Talin1^T2270A^). PDLIM5^T290A^ displayed a modest right-shift in decay (e.g., time to drop below 80% of peak: shift from 68 to 72 of normalized lifetime). Collectively, the temporal coupling of the AAK1 recruitment peak to the onset of disassembly, together with prolonged retention of phospho-null PDLIM5 and Talin1, supports a model in which locally recruited and constitutively active AAK1 promotes efficient release of key adhesome components during FA disassembly.

### The conserved LIDL motif of AAK1 mediates its direct interaction with PDLIM5

Guided by our live-cell TIRFM observations of PDLIM5-dependent AAK1 recruitment, we next mapped their molecular interaction by focusing on the predominant 961-amino-acid AAK1 splice variant expressed in RPE cells and annotated as canonical in Uniprot (Q2M2I8-1) and Ensembl database (ENST00000409085.9; Supplementary Fig. 9a). A defining feature of this isoform is the conserved LIDL motif at its extreme C-terminus, which is absent in BMP2K and suggests a distinct, AAK1 specific, physiological function (Fig. 7a). To elucidate the role of the LIDL motif, we performed an MS-based pull-down assay using a biotinylated peptide (Biotin-SSESSLPNLARSLLLVDQLIDL) representing AAK1’s flexible C-terminus (Fig. 7b, Supplementary Data 2). This experiment identified a group of PDZ domain-containing PDLIM proteins, including PDLIM5. A subsequent pull-down analysis further validated the interaction between full-length AAK1 and PDLIM5, showing a threefold enrichment compared to negative control (RPE^AAK1-/-^; Fig. 7c).

Weak and transient PDZ-mediated interactions will restrict signaling to specific subcellular regions by tethering AAK1 and its substrate in a localized complex, thereby increasing their effective local concentration^53^. This hypothesis prompted us to investigate the molecular basis of LIDL interactions using the shortened VDQLIDL peptide binding to PDLIM5 PDZ. X-ray crystallography revealed that the VDQLIDL peptide binds atypically to the PDLIM5 PDZ domain. Specifically, the AAK1 L961 residue fits into a hydrophobic pocket formed by PDZ residues I70 and L18, with the interaction further stabilized by a salt bridge between PDZ R17 and AAK1 D960 (Fig. 7d; Supplementary Fig. 9b, c; Supplementary Table 5).

Binding affinity measurements using biolayer interferometry (BLI) showed that the PDZ-PDLIM5 interaction has an apparent Kd of ~94 µM (Supplementary Fig. 10a). Notably, this value is comparable to the previously reported binding of PDLIM5 PDZ domain to the C-terminal ESDL motif of α-actinin^47,54^. This relatively weak LIDL-PDZ interaction between AAK1 and PDLIM5 likely provides specificity: AAK1 can transiently bind and phosphorylate PDLIM5, then dissociate and target new sites, facilitating repeated, localized phosphorylation events. To test this, we generated a GGGG-substitution mutant replacing the LIDL motif (RPE^AAK1-GGGG^), then assessed how disrupting LIDL-PDZ interaction affects AAK1-dependent cell migration. RPE^AAK1-GGGG^ had reduced cell migration speed by 15% compared to RPE^WT^ and RPE^AAK1-rescue^ (Fig. 7e, Supplementary Fig. 1g), highlighting the functional importance of the LIDL-PDZ PDLIM5 interaction in cell migration (Fig. 7f). In contrast, AAK1’s C-terminus is not required for AP2μ phosphorylation or TfR uptake (Supplementary Fig. 10b), consistent with our earlier findings (Fig. 3a). In combination with MS and TIRFM data (Fig. 5, Fig. 6), this detailed characterization of AAK1 and PDLIM5’s interaction reveals that transient and spatially restricted PDZ-mediated binding contributes to the capacity of AAK1 to accelerate cell migration.

## Discussion

FA dynamics are essential for efficient cell migration, enabling cells to establish, maintain, and release attachments to the ECM. While the molecular mechanisms of FA assembly are relatively well characterized, the processes that coordinate timely and spatially controlled disassembly remain comparatively less defined. In this study, we identify a new mechanism linking the kinase AAK1 to FA disassembly. Although AAK1 was previously considered functionally redundant with BMP2K in clathrin-mediated endocytosis, our data support a distinct, non-endocytic role for AAK1 as a specialized regulator of FA turnover. This regulation depends on spatiotemporally restricted recruitment of AAK1 and phosphorylation-dependent disengagement of its substrates. This kinase-centered mechanism contributing to FA turnover is likely to operate alongside established pathways that involve phosphatases or proteases, such as PTP-PEST or SHP2-mediated protein dephosphorylation^38,55^ or calpain-dependent cleavage of Talin1^56^, to weaken adhesion complexes and initiate disassembly.

Central to this new mechanism is the identification of PDLIM5 and Talin1 as direct substrates of AAK1. Three orthogonal observations converge on PDLIM5 and Talin1 as bona fide AAK1 substrates: (i) PRM shows that phosphorylation of PDLIM5 T290 and Talin1 T2270 decreases by >50% in RPE^AAK1,BMP2K-/-^; (ii) live-cell TIRFM reveals selective enrichment of AAK1 at mature FAs on collagen—where PDLIM5 and Talin1 are also present—whereas AAK1 remains diffuse under conditions that do not support FA formation; and (iii) AAK1 directly engages the PDZ domain–containing protein PDLIM5 via its C-terminal PDZ-binding motif LIDL, supported by peptide pull-down together with structural and biophysical validation. Dynamic measurements further connect these phosphorylation events to FA turnover: the phosphomimetic PDLIM5^T290D^ exhibits faster exchange by FRAP, whereas non-phosphorylatable PDLIM5^T290A^ and Talin1^T2270A^ show prolonged late-phase retention at FAs in lifetime-aligned TIRFM analyses. Collectively, these data support a model in which AAK1 recruitment to mature FA promotes phosphorylation-dependent substrate disengagement, facilitating timely FA disassembly and efficient migration. During early stages of FA formation, the PDZ domain of PDLIM5 binds α-actinin, which may be in complex with integrins^52,57^. As FAs progressively mature, the force-sensitive LIM domains avidly bind tensed actin, producing a strong, persistent FA association, facilitating actin crosslinking and FA maturation to generate traction force. Accumulation of PDLIM5-α-actinin complexes on tension-bearing actin-filaments locally enriches AAK1 at mature FAs. Our data show that the recruitment rate of AAK1 peaks concurrently with maximal PDLIM5 accumulation, positioning AAK1 to accelerate the transition from stable adhesion to disassembly. The selectivity of AAK1 for phosphorylating PDLIM5 is strengthened by the interaction of its LIDL motif with PDLIM5’s PDZ domain. This interaction could bring AAK1 in closer proximity to the PDLIM5 linker region, where T290 is located, and likely orientates itself to facilitate phosphorylation. Phosphorylation at T290 weakens PDLIM5 interactions at FAs. One plausible additional mechanism is PDZ-site competition. Both α-actinin (C‑terminal ESDL) and AAK1 (C‑terminal LIDL) present PDZ‑binding motifs that bind the PDLIM5 PDZ domain with similarly weak affinities in the tens-of-micromolar range^54^. Thus, local enrichment of AAK1 at mature adhesions could transiently occupy the PDZ pocket and reduce α-actinin engagement, further weakening PDLIM5 retention within the adhesome and biasing it toward a more exchangeable state.

AAK1 also phosphorylates Talin1, a mechanosensitive cytoskeletal adapter that binds integrin β tails and links them to F-actin, and undergoes force-dependent conformational transitions that regulate FA signaling^58^. Structural studies established an autoinhibited closed Talin1 conformation in which R12 forms a ‘lid’ over the FERM membrane/PIP2-binding surface^29^. This autoinhibited conformation is supported by charge complementarity at the F2–R12 interface: F2 residues K272/K274 contact E2288/D2297 within an acidic R12 region that includes E2288, E2294, and D2297.

Talin1’s F1–F2 segment is connected by a flexible linker and therefore samples a range of conformations^59^. In some of these conformations, the basic surface of F2 could come in closer proximity to phosphorylated T2270, thereby biasing the equilibrium toward the autoinhibited state. Interestingly, T2270 is located next to other reported R12 phosphosites (PhosphoSitePlus also lists S2273 and T2281), raising the possibility that other phosphorylation events could further modulate the charge of this helix. Consistent with this hypothesis, our live-cell analysis shows that preventing phosphorylation at T2270 increases Talin1 retention at FAs. This suggests that phosphorylation at this site promotes Talin1 turnover/dissociation and its functional engagement with the PM, integrins, and the actin cytoskeleton, ultimately influencing FA dynamics. More broadly, these findings fit a growing view that Talin1 integrates multiple kinase inputs acting on distinct rod domains to tune adhesion mechanics and dynamics across different cellular contexts^60^. For example, CDK1 binds Talin1 via an LD-motif interaction with the R8 domain and phosphorylates Talin1 at S1589, which reduces KANK adapter binding and alters the mechanosensitivity of the R7R8 module, providing a route for cell-cycle control of adhesion remodeling^61^.

Our findings also suggest evolutionary divergence between AAK1 and BMP2K. While AAK1 has specialized to regulate FA dynamics and cell migration in RPE cells, BMP2K remains critical in CME. The emergence of the AAK1 C-terminal LIDL motif in jawed vertebrates likely reflects evolutionary pressures linked to cell migration processes, such as neural crest development and/or mesodermal cell migration^62^. In C. elegans, SEL-5, a distant ortholog of AAK1 and BMP2K, functions independently of its kinase activity to regulate EGL-20/Wnt signaling during the migration of QL neuroblast daughter cells^63^. Yeast NAK orthologs (Prk1, Ark1, and Akl1) localize to actin patches, where they phosphorylate actin-related substrates to disassemble these structures after CCP scission. This resetting allows new rounds of endocytosis and actin assembly, which are critical for efficient vesicle trafficking in yeast. While vertebrate AAK1 and yeast Ark1/Prk1 both regulate actin dynamics through kinase activity, their varying biological contexts demonstrates the evolutionary adaptability of these regulatory mechanisms.

This work opens two promising avenues for future exploration. First, the identified feedback loop, in which PDLIM5 accumulates at FAs, recruits AAK1, and subsequently becomes phosphorylated, suggests a coupling between actomyosin-generated tension and AAK1-mediated signaling. Given that our data indicate PDLIM5 is not the sole interactor responsible for AAK1 recruitment to FAs (Fig. 5), it will be important to elucidate the broader set of mechanosensitive pathways involved in regulating AAK1 localization. Furthermore, recent phosphoproteomic analyses reveal that several phosphosites in the C-terminus of AAK1 robustly respond to mechanical stimuli in fibroblasts^64^, reinforcing a model in which AAK1 spatio-temporal positioning and substrate targeting at FAs are directly modulated by mechanical cues, such as cytoskeletal tension or ECM stiffness.

Second, selective targeting of AAK1 presents intriguing therapeutic potential^5^. Given the distinct localization and functional specialization of AAK1 compared to BMP2K, selective inhibitors of AAK1 could modulate FA dynamics and cell migration without perturbing BMP2K-mediated CME. Investigating how vertebrate cells balance endocytic and cytoskeletal regulation through distinct kinase networks promises important insights for developing targeted therapies, particularly for diseases involving aberrant cell migration, such as metastatic cancers and inflammatory conditions.

## Methods

### Computational screening and refinement of [L/I]xxQxTG motif in human proteins

We conducted a computational screen to identify human proteins containing [L/I]xxQxTG-based motifs. This query resulted in 187 sequences, from which duplicates were removed, yielding a total of 82 unique proteins (Supplementary Data 1). Secreted proteins and those with motifs located in extracellular regions were excluded based on UNIPROT Subcellular location annotation and not used for experimental validation. Based on the results of an in-vitro kinase assay, we refined the search to identify the consensus motif [LI]xxQ[LIMFV]TG, where X represents any residue except D or E. This refinement yielded a total of 48 proteins for in-cell validation by PRM-MS (Supplementary Data 1). All computational analyses were performed using custom Python and R scripts, which are accessible at https://github.com/sjspielman/endocytosis-slims-screen.

### In vitro NADH-coupled kinase assay

Continuous spectrophotometric in vitro NADH-coupled kinase assays were performed in transparent 96-well plates (Greiner UV Star). A master mix containing kinase buffer (30 mM HEPES, 150 mM NaCl, 10 mM KCl, 10 mM MgCl₂), 0.3 mM NADH, 0.6 mM PEP, and 0.8 µM AAK1/BMP2K kinase domain was prepared and added to each well. The expression and purification of AAK1 and BMP2K kinase domains in *E.coli* is described in later sections. Tested peptides (Genscript) were dissolved in kinase buffer to a 2 mM stock concentration and added to the reaction at final concentrations of 0 mM (to assess ATPase activity), 0.03, 0.07, 0.14, 0.21, 0.29, 0.43, 0.71, 1.14, and 1.34 mM. The reaction, with a total volume of 210 µl, was initiated by adding ATP to a final concentration of 0.43 mM. The microplate was then placed into a Clariostar spectrophotometer (BMG Labtech), shaken for 30 s at 100 RPM, and the decrease in absorbance was recorded at RT. Data were processed using MARS Data Analysis Software (BMG Labtech), analyzing the linear portion of the reaction to obtain the initial substrate conversion rate. This was achieved by fitting the absorbance decrease over time to a linear regression and calculating the reaction velocity. These data were subsequently analyzed in GraphPad Prism by fitting the initial velocities at all measured substrate concentrations to a nonlinear regression curve using the Michaelis-Menten equation. This analysis identified peptides phosphorylated by either AAK1 or BMP2K, where the decrease of NADH absorbance grew with higher peptide concentrations. In contrast, for non-phosphorylated peptides, the NADH absorbance decrease remained low and constant across all concentrations, reflecting the ATPase activity of the kinases.

### Experimental design - MS experiments

The workflow began with testing the presence of phosphorylated peptides with the consensus sequence L/IXXQXTG via PRM-MS after in vitro kinase assays with AAK1 and BMP2K kinase domains (experiments 6045 and 6138, Fig. 1h, i). Building on this, PRM-LC-MS experiment 5488 was conducted to test the presence of phosphorylated forms of the potential AAK1/BMP2K substrates in RPE^WT^ cells. The abundance of these phosphopeptides was subsequently quantified in RPE^WT^ and RPE^AAK1/BMP2K-/-^ which revealed that phosphosites on PDLIM5 (ILAQIpTGTEHLK) and Talin1 (QQLpTGHSK) were responsive to AAK1 and BMP2K deletion (Fig. 1j, k). To orthogonally validate these findings and to enhance signal detection, co-IP of PDLIM5 and Talin1 was followed by PRM-LC-MS quantification of ILAQIpTGTEHLK and QQLpTGHSK (experiment 5759). Label-free LC-MS/MS and data-independent acquisition (DIA) analyses complemented this workflow, providing a complete overview of AAK1/BMP2K-related proteome and phosphorylation changes (experiment 5488, Fig. 2c, d). Quantitative proteome and phosphoproteome profiling following acute AAK1/BMP2K inhibition was performed in experiment 5235 (Fig. 2a, b). Unless stated otherwise, cells were grown on plates coated with rat tail collagen I (Gibco A10483-01; 100 µg/mL in 20 mM acetic acid; 30 min at 37 °C), which were then washed with PBS. All experiments included at least four independent biological replicates. Other experimental MS procedures, describing co-IP and pulldown experiments, are provided in the Supplementary Information.

### PRM-LC-MS workflow for detecting phosphorylated peptides with [L/I]XXQXTG consensus sequence following in vitro kinase assay (6045)

The PRM method was set up using the retention time and m/z of both phosphorylated and non-phosphorylated version of each assayed peptide, and measured using the UltiMate 3000 RSLCnano system (Thermo Fisher Scientific) online connected to Impact II system (Bruker). Peptide samples taken directly from the in vitro kinase assay were loaded onto the pre-equilibrated analytical column (Pepmap C18 column, 2 µm particles, 300 μm × 15 cm; Thermo Fisher Scientific) operated at 40 °C and eluted at flow rate of 6 µl/min by 20 min long gradient (2-60% B; mobile phase A: 0.1% formic acid in MilliQ water; mobile phase B: 0.1% formic acid in 80% acetonitrile, 20% MilliQ water) followed by 5 min wash at 95% B and system re-equilibration.

MS/MS data were acquired in PRM mode, specifying individual phosphopeptides m/z values in separate methods (one method per monitored peptide) as per the expected modification status and charge state. The m/z values of the peptides were calculated from the peptide sequences using Skyline. Collision energies were estimated using the default values used for the data-dependent acquisition (DDA) mode and based on the given precursor m/z value (Supplementary Data 4), isolation width was set to 3 Th for all precursors.

The raw data were processed in DataAnalysis software (version 4.2) and MS/MS data were exported as Mascot generic files (MGF) files that were further searched using the in-house Mascot search engine (version 2.6.2) against database containing peptides of interest to aid with manual validation of the phosphorylation localization. Subsequently, the raw files were also loaded into the Skyline-daily software (version Skyline-daily-64_23_1_1_353) for validation of the expected peptidoform specific fragments.

### Quantitative analysis of selected phosphopeptides using PRM-LC-MS in RPE^WT^ and RPE^AAK1/BMP2K-/-^ cell lysates (5488)

Sample preparation: Total protein was extracted from RPE^WT^ and RPE^AAK1/BMP2K-/-^ cells using 8 M urea (50 mM Tris-HCl, pH 8.0) with protease and phosphatase inhibitors (Roche). For each cell line, four biological replicates, each from separate cultures, were processed. Post-homogenization, insoluble fractions were separated by centrifugation (20,000×g for 10 min at 8 °C). Protein concentrations in this and all subsequent experiments were quantified via tryptophan fluorescence (excitation: 295 nm, emission: 350 nm). The samples were reduced using 10 mM DTT (Merck), alkylated with 40 mM IAA (ThermoFisher), and quenched with 20 mM DTT. Digestion was performed using a Trypsin-Lys C (Promega) mixture at a 50:1 protein:protease ratio for 3 h at 37 °C, followed by dilution to 1 M urea using 50 mM Tris, pH 8 and an ON digestion with SoluTrypsin (Merck) at the same protein:protease ratio. The reaction was stopped with 0.2% TFA (Merck), and the insoluble fraction was separated by centrifugation (4700 × g for 5 min). Peptides were desalted using SepPak C18 columns 360 mg sorbent/cartridge (Waters). The cartridges were first washed with 1 ml of 100% methanol (Merck) and then equilibrated with 1 ml of 0.1% TFA (Merck) and 80% acetonitrile (ThermoFisher) and washed twice with 1 ml of 0.1% TFA. After loading the peptides, cartridges were washed three times with 1 ml of 0.1% TFA. Finally, the peptides were eluted into 2×1 ml of 0.1% TFA and 45% acetonitrile. Each of the samples were split into three vials (2×995 µl for phosphopeptide enrichment and 1 × 10 µl for direct analysis) and the solvent was evaporated in a SpeedVac concentrator (ThermoFisher). One vial of each sample was used for phospho enrichment which was carried out using the Fe-NTA Phosphopeptide Enrichment Kit (ThermoFisher), the second vial of each sample was enriched using TiO_2_ Phosphopeptide Enrichment Kit (ThermoFisher) and the last vial of each dried sample was immediately extracted into a LC-MS vial. Exctraction into LC-MS vials was performed by 2.5% formic acid in 50% acetonitrile (ACN) and 100% ACN with the addition of polyethylene glycol (final concentration 0.001%^65^; and concentrated in a SpeedVac concentrator (Thermo Fisher Scientific).

The data were acquired using the using RSLCnano system online connected to Orbitrap Exploris™ 480 Mass Spectrometer with EASY Spray ion source (Thermo Fisher Scientific) installed. Prior to LC separation, digests were online concentrated and desalted using trapping column (300 μm × 5 mm, μPrecolumn, 5μm particles, PepMap™ Neo Trap Cartridge, Thermo Fisher Scientific). After washing of trapping column with 0.1% FA, the peptides were eluted (flow 300 nl/min) from the trapping column onto separation column (Aurora C18, 1.7 µm particles, 75 μm × 250 mm, P/N AUR3-25075C18-CSI; IonOpticks) operated at 50 °C by linear 60 min long gradient (3–37% B; mobile phase A: 0.1% formic acid in MilliQ water; mobile phase B: 0.1% formic acid in 80% acetonitrile, 20% MilliQ water).

MS data were acquired in a product ion scan mode with additional survey scan measurement. The product ion scan mode was set for the expected and peptide forms of interest (Supplementary Data 4) monitored during the whole LC-MS run. Product ion spectra were set at follows: custom AGC target of 3000 %, automatic maximal injection time mode, isolation width 1.2Th, relative fragmentation energy 30 %, orbitrap analyzer resolution 60,000, first mass m/z 120.

The data for all the peptides were processed and manually evaluated using the Skyline-daily software (version Skyline-daily-64_23_1_1_459) to identify the peaks corresponding to all tested peptides. The peaks corresponding to PDLIM5 ILAQIpTGTEHLK and Talin1 QQLpTGHSK were then quantified. Details of the downstream data analysis for this and all subsequent MS-based experiments are provided in the Supplementary Information.

### Quantification of the phosphopeptide ILAQI(pT)GTEHLK from immunoprecipitated PDLIM5 and QQL(pT)GHSK from immunoprecipitated Talin1 (5759)

Sample preparation: for the PDLIM5 and Talin1 immunoprecipitation, total protein was extracted from RPE^WT^, and RPE^AAK1/BMP2K-/-^ cells as described above. For each experiment and each cell line, three biological replicates from separate cultures were processed. Post-homogenization, insoluble fractions were separated by centrifugation (10,000 g for 20 min at 8 °C). Talin1 and PDLIM5 were isolated from 6 mg of protein per sample each using 30 µg of anti-Talin1 antibody 97H6 (BioRad) or 30 µg of anti-PDLIM5 antibody A14933 (Antibodies.com) and 70 µl A/G Agarose beads (Pierce™ Protein A/G Agarose, 20422) per sample, incubated overnight (ON) at 4 °C.

The beads were washed five times in a buffer containing 50 mM Tris, 1 mM EGTA, 1 mM MgCl2, 150 mM NaCl. The buffer was removed and 100 µl of 8 M urea (50 mM Tris-HCl, pH 8.0) was added to each sample. The immunoprecipitate was reduced, alkylated and digested as described above. The reaction was stopped with 1% TFA (Honeywell), insoluble fraction was separated by centrifugation (4700×g for 10 min). For desalting, we followed published protocol using stage tips with SDB–RPS filler material (Empore material 3 M^66^). Desalted peptides were concentrated in a SpeedVac concentrator (Thermo Fisher Scientific). Peptide concentration and total amount were estimated from a small aliquot measured on an UltiMate 3000 RSLCnano system (Thermo Fisher Scientific) online connected to an Impact II system (Bruker). Quantification was based on the area under the total ion chromatogram curve, with an external calibration curve prepared using HeLa digest standards (Pierce, 88329). Phospho enrichment was carried out using the Fe-NTA Phosphopeptide Enrichment Kit (ThermoFisher, A32992) according to the manufacturer instructions. The enriched phosphopeptide mixture was then subjected to the concentration estimation procedure as mentioned above.

The samples were measured using the using RSLCnano system online connected to Orbitrap Exploris™ 480 Mass Spectrometer with EASY Spray ion source (Thermo Fisher Scientific) installed. The online concentration, desalting and elution procedures are identical to those used for experiment 5488.

MS data were acquired in a product ion scan mode with additional survey scan measurement. The product ion scan mode was set for the expected and peptide forms of interest (Supplementary Data 4) monitored during the whole LC-MS run. Product ion spectra were set at follows: custom AGC target of 3000 %, automatic maximal injection time mode, isolation width 1.2Th, relative fragmentation energy 30 %, orbitrap analyzer resolution 60,000, first mass m/z 120.

The data was processed and analyzed as in experiment 5488, searching for and quantifying the

PDLIM5 ILAQIpTGTEHLK and TLN1 QQLpTGHSK phosphopeptides.

### Whole cell LFQ proteome and phosphoproteome profiling using DIA and RPE^WT^ and RPE^AAK1/BMP2K-/-^ cell lysates (5488)

Sample preparation and LC are specified in the section Quantitative analysis of selected phosphopeptides using PRM and RPE^WT^, RPE^WT^LP and RPE^AAK1/BMP2K-/-^ cell lysates.

Data were acquired in a DIA mode. The survey scan covered m/z range of 350-1400 at a resolution of 60,000 (at m/z 200) and a maximum injection time of 55 ms (normalized AGC target 300%). HCD MS/MS (27% relative fragmentation energy) were acquired in the range of m/z 200-2000 at 30,000 resolution (maximum injection time 55 ms, normalized AGC target 1000%). Overlapping windows scheme in the precursor m/z range from 322 to 997 were used as isolation window placements (Supplementary Data 4). A pooled solution was prepared from all individual samples and analyzed by gas-phase fractionation (GPF), comprising six GPF analyses each covering an approximate m/z range of 100 (Supplementary Data 4). Raw data were converted to mzML files using msconvert (version 3.0.21193-ccb3e0136), with peakPicking (Vendor) and demultiplex (overlap only, 10ppm) filters applied.

MzML files were processed in DIA-NN^67^ version 1.8 in library free mode against a modified protein contaminant (cRAP) database (based on http://www.thegpm.org/crap/; 111 sequences in total) and UniProtKB protein database for *Homo Sapiens* (https://ftp.uniprot.org/pub/databases/uniprot/current_release/knowledgebase/reference_proteomes/Eukaryota/UP000005640/UP000005640_9606.fasta.gz; version 12/2022, number of protein sequences: 20,594). Fractions before and after phosphopeptide enrichment were processed in DIA-NN separately, with distinct settings applied to each. For non-enriched samples, the following parameters were used: no optional modifications, carbamidomethylation as a fixed modification and trypsin/P as the digestion enzyme with 1 allowed missed cleavage and peptide length of 7-30 amino acids. False discovery rate (FDR) control was set to 1%. For phosphopeptide enriched samples, search parameters included phosphorylation (STY) as a variable modification, carbamidomethylation as a fixed modification, and trypsin/P as the digestion enzyme with a maximum of one missed cleavage and a peptide length of 7–30 amino acids. False discovery rate (FDR) was set to 1%. For both sample types, MS1 and MS2 accuracies as well as scan window parameters were set based on the test searches. GPF analyses were included in the final search and match between runs (MBR) was switched on.

The main DIA-NN report was processed using the software container environment (https://github.com/OmicsWorkflows), version 4.7.7a. Processing workflow is available upon request. For non-enriched samples, it covered: a) removal of low-quality (Global.Q.Value or Global.PG.Q.Value > 0.01) and cRAP associated precursors, b) filtering out proteins not quantified in at least 60% of replicates, c) PG.MaxLFQ intensities log2 transformation and normalization using loessF algorithm, d) imputation of missing values using random distribution data (d-value 1.8, w-value 0.3), e) differential expression analysis using LIMMA statistical test with Benjamini-Hochberg correction.

Processing workflow for phosphopeptide enriched samples covered: a) removal of low-quality (Global.Q.Value or Global.PG.Q.Value > 0.01) and cRAP associated precursors, b) precursors table grouping on the sequence and modifications concatenated information (returning maximum of all rows in Precursor. Quantity and Precursor. Normalized columns) with pivoting using the Run column; c) filtering precursors with at least 1 phosphorylation; d) log2 transformation and loessF normalization of Precursor.Normalized values (separately for TiO2 and IMAC enriched solution results); e) imputation of data using random distribution data (d-value 1.8, w-value 0.3) separately for TiO2 and IMAC enriched solution results after filtering out precursors not quantified in at least 60% of replicates; f) differential expression analysis using LIMMA statistical test with Benjamini-Hochberg correction.

We were able to identify 7042 proteins. Out of these, 132 were upregulated upon perturbation, and 173 were downregulated upon perturbation.

Phosphoproteome analysis consisted of 17495 total phosphopeptides, out of which 13829 were identified confidently enough for statistical analysis, belonging to 4463 total phosphoproteins out of which 414 were dysregulated.

### Whole cell proteome profiling with SILAC labeling, using DDA and RPE^WT^ and RPE^WT^LP cell lysates (5235)

Sample preparation: RPE^WT^ cells were grown in DMEM for SILAC (ThermoFisher) supplemented with 10% dialyzed fetal calf serum (Invitrogen). L-arginine (Merck), L-lysine (Merck) and L-proline (Merck), were added to the “light” media, while L-arginine 13C6 and L-lysine 13C6, 15N2 (CK Isotopes Ltd) to the “heavy” media at the same concentrations. The amino acid concentrations were based on the formula for DMEM (Merck). Once prepared, the SILAC media was mixed and filtered through a 0.22-μm filter (Merck) and stored at 4 °C. The cells were passaged in SILAC media for 2 weeks (or five cells doublings) before harvesting to ensure complete incorporation of isotopic amino acids. Incorporation check which showed >98% heavy amino acid incorporation. Cells for the whole cell proteome profiling were treated with either 10 uM of LP inhibitor for 6 h or DMSO as a control, then harvested into 8 M urea (50 mM Tris-HCl, pH 8.0) with protease and phosphatase inhibitors (Roche). Eight paired cultivations (inhibitor and DMSO treatment) were done using SILAC channels swap with 4 replicates for each combination (8 replicates in total).

The subsequent procedures were identical to those used for experiment 5488.

LC-MS/MS analyses were done were done using UltiMate 3000 RSLCnano system connected to Orbitrap Exploris™ 480 Mass Spectrometer with EASY Spray ion source installed (Thermo Fisher Scientific). Prior to LC separation, digests were online concentrated, desalted, and eluted as described before onto an analytical column separation column (EASY-Spray column, 75 μm ID, 500 mm long, 2 μm particles, Thermo Fisher Scientific) by 120 min gradient program (5–37% of mobile phase B; mobile phase A: 0.1% formic acid in water; mobile phase B: 0.1% formic acid in 80% ACN). Columns were heated to 40 °C.

MS data were acquired using DDA (cycle time 2.5 s). Survey scan was set to *m/z* 320-2000 with the resolution of 120,000 (at *m/z* 200), normalized target value of 250% and maximum injection time of 500 ms. HCD MS/MS spectra (isolation window *m/z* 1.2, 30% relative fragmentation energy) were acquired from 120 *m/z* with a relative target value of 100 % (intensity threshold 1×10^3^), resolution of 30 000 (at *m/z* 200) and automatic maximum injection time. Targeted Mass Difference method node was used to define presence of the SILAC pairs (delta masses 8.0142 and 6.0201, 5ppm tolerance).

Raw data from both phosphopeptide enriched as well as non-enriched solutions were evaluated together using MaxQuant software v2.0.3.0^68^ with inbuilt Andromeda search engine^69^ using default parameters unless otherwise noted. Fractions design was set to enable MBR among all replicates of individual solutions type. Phosphopeptide enriched solutions analyses were set to a different parameter group and PTM tag was set to True. Search was done against the previously mentioned protein databases of *Homo Sapiens* and cRAP. Multiplicity of 2 was set, using Lys8 and Arg6 as heavy labels. Modifications were set as follows for database search: oxidation (M), deamidation (N, Q), acetylation (Protein N-term) and phosphorylation (S, T, Y; only for the Ti and IM analyses) as variable modifications, with carbamidomethylation (C) as a fixed modification. Enzyme specificity was set to tryptic with 2 allowed miscleavages. Only peptides and proteins with FDR threshold under 0.01 were considered.

The txt report files were further processed using the software container environment. Processing workflow is available upon request. Protein level information processing steps were as follows: a) reverse hits and cRAP removal; b) individual channels protein intensities calculation using total protein intensity per raw file (Intensity columns) and corresponding normalized H/L ratios (Ratio H/L columns); c) protein group intensities log2 transformation, normalization (loessF) and back log2 transformation; d) inhibitor/DMSO ratios calculation; e) statistical evaluation of inhibitor/DMSO log2 ratios difference from 0 using limma R package, with Benjamini-Hochberg correction.

Phosphopeptides were processed from evidence.txt, Phospho(STY)Sites.txt and proteinGroups.txt tables: a) evidences filtering for those with at least 1 phosphorylation; b) removal of peptides from cRAP proteins; c) remaining evidences grouping on the sequence and modifications concatenated information (sum of all rows in Intensity columns and median from ratio columns were returned) with pivoting using the Experiment column; d) calculation of grouped evidence intensities using total evidence intensity per raw file (Intensity columns) and corresponding normalized H/L ratios; e) log2 transformation and loessF normalization of channel intensities (separately for TiO2 and IMAC enriched solution results) and back log2 transformation of normalized values; f) inhibitor/DMSO ratios calculation using values calculated in the previous step and their log2 transformation; g) median log2 ratio calculation from TiO2 and IMAC enriched solution results; h) statistical evaluation of inhibitor/DMSO log2 ratios difference from 0 using limma R package, with Benjamini-Hochberg correction.

Using this approach, 2973 proteins were detected. Out of these, 103 were upregulated upon perturbation and 73 were downregulated upon perturbation.

The phosphoproteome analysis consisting of 18622 total phosphopeptides, out of which 9804 were identified confidently enough for statistical analysis, belonging to 4246 total phosphoproteins out of which 490 were dysregulated.

### Cell lines and cell culture

All cell lines were obtained from ATCC: hTERT-RPE1 (ATCC CRL-4000)^70^, MDA-MB-231 (ATCC HTB-26), SH-SY5Y (ATCC CRL-2266), A-431 (ATCC CRL-1555), Saos-2 (ATCC HTB-85) and cultured in DMEM, high glucose, Glutamax medium (31966-021, Thermo Fisher Scientific-Gibco) supplemented with 10% FBS (Sigma-Aldrich, F7524). All cells were cultured at 37 °C under 5% CO_2_. Cell lines were routinely tested for absence of mycoplasma contamination with MycoAlert Mycoplasma Detection Kit (Lonza, LT07-318).

### Generation of single knockout cell lines RPEAAK1-/- and RPEBMP2K-/- cells using CRISPR-Cas9

RPE^AAK1-/-^ and RPE^BMP2K-/-^ cells were generated using CRISPR-Cas9. Guide RNAs (gRNAs) targeting exon 3 of BMP2K (GenBank ID: 55589) and exon 2 of AAK1 (GenBank ID: 22848) were designed using the GenCRISPR™ system (GenScript). For both genes, a single plasmid encoding the gRNA, Cas9, and eGFP was used for co-transfection into RPE cells. The selected BMP2K gRNA (T1: 5′-TTCCTCGTGCGTACTCACGG-3′) and AAK1 gRNA (T1: 5′ TTTCTGGTGAGGACAAGCAA-3) were transfected with Lipofectamine (Invitrogen) and eGFP-positive cells were sorted using fluorescence activated cell sorting (FACS) to enrich for successfully transfected cells. Single-cell clones were isolated and screened via Sanger sequencing. For BMP2K, clones T1-2 (−1/ + 2 bp), T1-21 (−1/ + 1 bp), and T1-22 ( + 1/ + 1 bp) exhibited biallelic knockouts, while for AAK1, clones T1-16 (−10/−10 bp), T1-43 (−2/ + 2 bp), and T1-45 (−4/ + 2 bp) showed biallelic knockouts. Real time quantitative polymerase chain reaction (RT-qPCR; Supplementary Fig. 11) and Western blot analyses (Supplementary Fig. 1c) confirmed the absence of mRNA and protein expression for both genes. Western blotting was performed by lysing cells grown to 80% confluence in a 6-cm dish with 100 μL of lysis buffer (75 mM Tris, 150 mM NaCl, 2% Triton X-100, 7.5 mM EDTA, 7.5 mM EGTA). The resulting lysates were separated on a 10% SDS-PAGE gel and transferred to nitrocellulose membrane, which were then stained in 0.2% Ponceau S Staining Solution and imaged as a loading control. Membranes were then incubated in blocking solution (3% BSA in TBST) and probed with the anti-AAK1 antibody EPR5126^2^ (ab134971, Abcam), which specifically targets the kinase domain of AAK1 (residues 1–365) or anti-BMP2K Antibody E-8 (sc-514681, Santa Cruz), all in 1:1000 dilution. Blots were imaged either on Odyssey CLx (Li-Cor) or ChemiDoc (Bio-Rad). Blots were analyzed in Image Studio (Li-Cor) or Fiji and Ponceau S staining or the indicated loading controls were used to verify comparable loading and transfer. Immunoblots are representative of at least two independent biological replicates and uncut blots are presented in Source Data file. To further confirm the absence of mRNA expression, RT–qPCR was performed. Total RNA was isolated from one 10-cm dish per cell line and reverse-transcribed into cDNA using the Transcription First Strand cDNA Synthesis Kit (Roche), with 500 ng of RNA used as input. The resulting cDNA was analyzed by RT-qPCR using the LightCycler 480 SYBR Green I Master Kit (Roche) on a LightCycler 480 II instrument (Roche), following the manufacturer’s instructions. Ct values were determined using the automated Second Derivative Maximum Method in the LightCycler 480 software (Roche). Relative gene expression levels were calculated by normalization to glyceraldehyde 3-phosphate dehydrogenase (GAPDH). The primer sequences used were: AAK1: forward, GGACAAGCAATGGGATGAAATG; reverse, GCCCTGAAAGATCCCTCATTAT; BMP2K: forward, CAGAAGAGGAACTATTGGACAGAG; reverse, GTGGAGCAGAAAGTGAGTCTAC.

All clones exhibited normal growth rates and the validated clones were pooled into a single knock-out cell line for both RPE^AAK1-/-^ clones and separately for RPE^BMP2K-/-^ clones. Wild type isogenic clones were maintained as a negative control to generate isogenic pairs with mutant cell lines.

### Generation of double-knockout AAK1/BMP2K RPE^AAK1/BMP2K-/-^ cell line

Lentiviral particles carrying the sgRNA targeting exon 2 of BMP2K, along with Cas9 and eGFP (Edit-R™ CRISPR-Cas9 Gene Engineering with All-in-one Lentiviral Cas9 and sgRNA, Horizon), were used to infect the RPE^AAK1-/-^ cells. Two days post-infection, eGFP-positive population was enriched using FACS. Single-cell clones were isolated by limiting dilution and expanded. Genomic DNA from these clones was analyzed using Sanger sequencing to confirm biallelic knockout of BMP2K. Loss of BMP2K and AAK1 protein expression was validated by Western blot analysis (Supplementary Fig. 1c). In addition, DIA-MS was performed to independently confirm the absence of both AAK1 and BMP2K proteins (Supplementary Fig. 12), thereby validating the successful generation of the RPE^AAK1,BMP2K⁻/⁻^ cell line.

### Cloning and constructs

All constructs were generated using Gibson Assembly with vector pMIB6^71^ that was linearized with NotI and SalI to prepare it for the insertion of cDNA. The mScarlet-FLAG-G4S-BMP2K construct was assembled using BMP2K cDNA (NM_198892.1), generously provided by Linton Traub. The cDNA for AAK1 (NM_014911.4), obtained from Genscript in the vector pcDNA3.1-C-(k)DYK, was used to assemble both mScarlet-3×FLAG-G4S-AAK1 and its C-terminal mutant variant, mScarlet-3×FLAG-G4S-AAK1-GGGG, in pMIB6.

An internally-tagged human PDLIM5 construct was synthesized as a single gBlock by IDT for Gibson assembly. A HaloTag, followed by a G4S linker and a 3×myc tag, was inserted after amino acid 199 of PDLIM5 to generate the PDLIM5^WT^ construct. The sequence was codon-optimized to reduce secondary structures, facilitate synthesis and ensure siRNA resistance. Point mutations were introduced into the pMIB6-PDLIM5-HaloTag construct to generate two variants: a phosphoablated form (PDLIM5^T290A^) and a phosphomimetic form (PDLIM5^T290D^), both through PCR-based site-directed mutagenesis. The lentiviral construct mNeonGreen-paxillin encoding human paxillin was obtained from Addgene as a gift from Dorus Gadella (Addgene plasmid #176106^72^;). Construct mcherry-PH was a gift from Narasimhan Gautam (Addgene plasmid # 36075)^73^. The lentiviral construct mRuby2-Paxillin in pLVX vector^74^ and eGFP-CLCa in PMIB6 vector were kindly provided by the Schmid lab. Lentiviral construct Lifeact-mStayGold was a gift from the Griffiths lab, and VPS29-mClover for transient expression was a gift from the Luzio lab. The mEmerald–Talin1 construct encoding mouse Talin1 for transient expression was a gift from Michael Davidson (Addgene plasmid #62763), and the Talin1 phospho-null mutant (T2270A) was generated by site-directed mutagenesis.

### Retrovirus and lentivirus preparation and cell line engineering

Retroviral particles were generated by co-transfecting HEK 293 T cells with a complex formed by polyethylenimine (PEI) and three plasmids: Gag-Pol, VSV-G, and the retroviral transfer vector pMIB6. Similarly, lentiviral particles were prepared by co-transfecting cells with the pCMV-dR8.91 packaging vector encoding Gag-Pol, VSV-G, and the lentiviral transfer vector pLVX.

### Reconstituting RPE^AAK1-/-^ cell lines with mScarlet-AAK1^WT^ and mScarlet-AAK1^GGGG^ constructs

The RPE^AAK1-/-^ cell lines were reconstituted with mScarlet-3×FLAG-G4S-AAK1 or GGGG mutant retrovirus. The mScarlet-AAK1^WT^ or mScarlet-AAK1^GGGG^ cell line were sorted by FACS into populations of lowest expression, and cell pools were further analyzed by western blotting (Fig. S1g). The population with the AAK1 expression closest to that of RPE^WT^ cell lines was selected for further experiments.

### Cell line engineering

To establish cell lines co-expressing mScarlet-AAK1 or mScarlet-BMP2K with eGFP-CLCa, mScarlet-AAK1 with PDLIM5-HaloTag and Lifeact-mStayGold, mScarlet-AAK1 with PDLIM5-HaloTag and mNeonGreen-Paxillin, and eGFP-CLCa with mRuby2-Paxillin, RPE cells underwent sequential infection with viral particles to introduce the desired fluorescently tagged proteins. Following infection, FACS was used to isolate populations with consistent expression levels, approximating endogenous expression for each tagged protein. Expression of each marker was confirmed by western blotting using the respective antibodies listed in Supplementary Table 7, with a dilution of 1:1000.

### Engineering of cell lines expressing PDLIM5 variants

RPE^WT^ cells were infected with retroviral particles carrying PDLIM5 WT, PDLIM5 T290A, and PDLIM5 T290D constructs. FACS was employed to isolate populations with high levels of the fluorescent marker, matching the endogenous PDLIM5 expression levels in RPE cells, thereby ensuring uniform expression across the selected cell lines. Consistent and equal expression levels of PDLIM5 and its mutants were confirmed by Western blot analysis (Fig. S1e); *n* = 2.

AAK1, PDLIM5, or AP2µ mRNAs were depleted using two rounds of siRNA-mediated knockdown (Lipofectamine RNAiMax) at 16 and 36 hours post-plating; assays were performed on day four. AAK1 was targeted with an ON-TARGETplus siRNA pool (Dharmacon; sequences: CCUCGGACCUCUCAACAAA, ACAAAAGGCCGAUAUUUA, GGAAGGUGGAUUUGCUAUU, GCGUAUUCUCAGUGACGUA). PDLIM5 was targeted with the siRNA sequence CAACAUGCCUCUGACAAUC. Ectopic PDLIM5 constructs (including phosphomimetic/phosphoablated variants) were rendered siRNA-resistant by mutation to CAACATGCCCCTGACTATT. AP2µ was targeted in its 3’ untranslated region with siRNA 5’CCUACUCUGCUUUGGGAUAUU3’ (Dharmacon). Knockdown efficiencies (typically ~90% for AAK1, 95% for PDLIM5, ~80% for AP2µ) were confirmed via western blotting in parallel with each experiment (Supplementary Fig. 1d, e, h); *n* = 2. Antibodies used are listed in Supplementary Table 7, all were used in 1:1000 dilution.

### Cell migration assay

Imagelock 96-well plates (Sartorius, BA-04856) were coated with Collagen I, Rat tail (Gibco, A10483-01; 100 µg/mL in 20 mM acetic acid; 30 min at 37 °C) and washed once with PBS. Wild-type or siRNA-treated cells were seeded to form uniform monolayers ON at the following densities: 35,000 cells/well (RPE), 25,000 cells/well (SAOS2 and A-431), 90,000 cells/well (SH-SY5Y), and 30,000 cells/well (MDA-MB-231). Consistent scratches were created using the Essen Bioscience WoundMaker®, and wells were washed twice with PBS to remove debris. Complete DMEM (100 µl) was added to each well, and the plate was equilibrated in the Incucyte® (for 5 min to stabilize CO_2_ and temperature. IncuCyte S3 Live Cell Analysis System (Sartorius) was equipped with a Basler Ace 1920-155 um compact camera and 10X objective. Images were captured every 30 minutes, and wound width was analyzed using the Incucyte® Scratch Wound Module. Percentage wound closure after 6 h was calculated relative to the initial scratch width.

Drug pre-treatments were applied as follows: LP-935509 inhibitor (10 µM, MedChemExpress LLC) and LX-9211 (10 µM, MedChemExpress LLC) for 6 h, and CK666 (100 µM, MedChemExpress LLC) for 2 h prior to scratch formation, with all inhibitors maintained in the media throughout the experiment. Statistical analysis was performed from three independent biological replicates (24 wells per conditions), using one-way ANOVA followed by Tukey’s post hoc test.

### TfR and integrin α2 internalization assay

The internalization of TfR and integrin α2 was measured using primary antibodies anti-TfR mAb (OKT9, Bio-X-Cell) and anti-Integrin α2 (CD49b, HAS-4, Novus Biologicals, NB100-2608) respectively. Knockout cells or cells with AP2µ siRNA-mediated knockdown were seeded at 20,000 cells/well into 96-well plates and cultured ON at 37 °C. To minimize non-specific antibody binding to the plate surface, the coating conditions were optimized as follows: Before seeding, plates were coated with PLL (Merck P8920; 0.01% in ddH2O, prepared from a 0.1% stock) for 10 min at room temperature, then washed once with PBS. Plates were then coated with bovine skin gelatin (Merck G9382; 0.2% in ddH2O containing 2% sucrose) for 20 min at room temperature, cross-linked with 0.1% glutaraldehyde for 10 min, and washed three times with PBS. Finally, plates were equilibrated with complete medium for 30 min at room temperature before use. For the internalization assays, cells were incubated with 4 μg/mL of the corresponding primary antibody at 37 °C for various time points. To stop internalization, the cells were immediately cooled to 4 °C. Cell surface-bound antibodies were removed through five washes with a cold acidic solution (0.2 M acetic acid, 0.2 M NaCl, pH 2.5, 4 °C). The cells were then washed with PBS and fixed using 4% paraformaldehyde (PFA) in PBS for 20 min at RT. After fixation, cells were permeabilized with 0.1% Triton X-100 in PBS for 10 min. Internalized antibodies were detected using a goat anti-mouse horseradish peroxidase (HRP)-conjugated secondary antibody (Life Technologies), followed by development with OPD. The enzymatic reaction was stopped with 2 M H2SO4, and absorbance was measured at 490 nm using a BMG CLARIOstar Microplate Reader.

### In-cell ELISA for quantification of AP2μ phosphorylation at T156

In-cell ELISA assays were conducted using Corning Costar Stripwell 96-well plates (Thermo Fisher Scientific). Prior to seeding, the plates were coated as described above to minimize non-specific antibody binding to the plate surface. RPE cell lines were seeded at a density of 30,000 cells/well and cultured ON. The next day, cells were treated with 10 µM AAK/BMP2K inhibitor (LP, LP-935509, MedChemExpress, dissolved in DMSO) for 3 h at 37 °C in a humidified incubator with 5% CO2. DMSO-treated cells were used as controls. Following treatment, cells were fixed with 4% PFA for 15 min at RT and washed thoroughly with PBS. Cells were permeabilized with 0.1% Triton X-100 for 15 min at RT. Blocking was performed with 5% bovine serum albumin (BSA) in TBST (TBS + 0.1% Tween 20) for 1 h at RT. Cells were then incubated ON at 4 °C with anti-pAP2μ (T156) (D4F3) antibody (7399, Cell Signaling Technology), diluted 1:100 in 5% BSA/TBST. The following day, cells were washed three times with PBS and incubated with an anti-rabbit HRP-conjugated secondary antibody (Promega) in 5% BSA/TBST for 2 h at RT. After five additional washes with PBS, the detection step was carried out using o-phenylenediamine dihydrochloride (OPD, P6662, Merck) for 30 min, and the reaction was stopped using 3 M HCl. Absorbance was measured at 492 nm using a BMG CLARIOstar Microplate Reader. To normalize for protein content between wells, a BCA assay (Pierce) was performed. Results from five independent biological replicates were pooled, averaged, and presented as mean ± SD. Statistical significance was assessed using one-way ANOVA followed by Tukey’s post hoc analysis.

### Sample preparation, live-cell time-lapse TIRFM and epifluorescence imaging

High-precision #1.5 round coverslips (Marienfeld, 0117650) were acid-washed in 1 M HCl, rinsed thoroughly with ddH₂O, and coated with one of the following substrates, depending on the experiment. For collagen coating, coverslips were incubated with rat tail collagen I (Gibco, A10483-01; 100 µg/mL in 20 mM acetic acid) for 30 min at 37 °C. For poly‑L‑lysine (PLL) coating, coverslips were incubated with 0.01% PLL (Merck, P8920; prepared by diluting a 0.1% stock in ddH₂O) for 30 min at 37 °C. For transferrin coating, coverslips were incubated with transferrin (Merck, T5391; 0.1 mg/mL in PBS) for 30 min at 37 °C. After coating, coverslips were washed once with PBS and used immediately.

For experiments comparing ECM-dependent recruitment (collagen vs PLL), cells were reseeded onto the corresponding coated coverslips and imaged 2 h after seeding, to capture early, adhesion-dependent changes in AAK1 localization. All live-cell imaging was performed at 37 °C and 5% CO₂ in complete FluoroBrite™ DMEM supplemented with 10% FBS and 4 mM L‑glutamine (Gibco). Imaging was performed on a Zeiss Elyra PS1 inverted microscope equipped with a Zeiss 100×/1.49 NA Apo TIRF objective and Definite Focus. TIRFM or epifluorescence imaging was used as indicated. Filter sets were selected to minimize spectral bleedthrough, and laser power/exposure times were kept as low as possible to maintain signal-to-noise while limiting phototoxicity. Single-color controls were imaged under identical settings to confirm negligible bleedthrough between channels.

AAK1 recruitment to FAs (Fig. 5b). RPE cells expressing mScarlet–AAK1 and mNeonGreen–paxillin were reseeded onto collagen- or PLL-coated coverslips and imaged by TIRFM 2 h after seeding.

AAK1 colocalization with PDLIM5 and F-actin (Fig. 5c). RPE cells expressing mScarlet–AAK1, PDLIM5–HaloTag, and Lifeact–mStayGold were reseeded onto collagen-coated coverslips and imaged by TIRFM 2 h after seeding. For PDLIM5–HaloTag visualization, cells were labeled overnight with JF650 HaloTag dye (1:5000 dilution) and washed three times with complete FluoroBrite™ DMEM before imaging.

AAK1 localization relative to Talin1 and PDLIM5 (Fig. 5d). RPE cells stably expressing mScarlet–AAK1 and PDLIM5–HaloTag were transiently transfected with mEmerald–Talin1, then reseeded onto collagen-coated coverslips and imaged by TIRFM 2 h after seeding (typically 36–48 h post-transfection). PDLIM5–HaloTag was labeled as above.

AAK1 endosomal localization relative to VPS29 and integrin α2 (Fig. 5b, e, Supplementary Fig. 8a). RPE cells expressing mScarlet–AAK1 ( ± PDLIM5–HaloTag, where indicated) were transiently transfected with VPS29–mClover. At 48 h post-transfection, cells were reseeded onto collagen- or PLL-coated coverslips and imaged 2 h after seeding by epifluorescence microscopy (or TIRFM where indicated). To label integrin α2, cells were incubated for 60 min at 37 °C in FluoroBrite™ DMEM containing 4 µg/mL Alexa Fluor® 647–conjugated anti-integrin α2 antibody (HAS‑4), then washed with warm medium prior to imaging. For experiments additionally visualizing PDLIM5–HaloTag, cells were labeled overnight with JF650 HaloTag dye as described above.

Negative controls for FA-independent patterns and illumination artifacts (Fig. 5F, Supplementary Fig. 8B–D). To assess AAK1 localization under conditions that do not support FA formation, cells expressing mScarlet–AAK1, mNeonGreen–paxillin, and PDLIM5–HaloTag were reseeded onto transferrin-coated coverslips and imaged 2 h after seeding (Fig. S8B). To control for uneven attachment or TIRFM illumination, three additional controls were performed: (i) wheat germ agglutinin (CF®405 M WGA, Biotium) plasma membrane labeling (5 µg/mL for 10 min at room temperature, followed by two washes in medium) in cells seeded on collagen and imaged 2 h after seeding (Figure S8C); (ii) cytosolic BFP co-expression and imaging under identical TIRFM settings (Fig. 5F); and (iii) Cells were transiently transfected with mCherry–PH (a PtdIns^4,5^P₂ plasma-membrane probe). At 24 h post-transfection, cells were reseeded onto collagen-coated coverslips and imaged by TIRFM after 2 h (Fig. S8d).

### Quantitative analysis of BMP2K and AAK1 colocalization with egfp-CLCa in live cells

RPE cells co-expressing EGFP-CLCa with either mScarlet-AAK1 or mScarlet-BMP2K were seeded onto collagen-coated coverslips and incubated for 24 h. Single TIRFM snapshots of live cells were acquired from three biological replicates (AAK1: *n* = 15 cells; BMP2K: *n* = 17 cells).

Prior to automated quantification, in-focus cells were manually masked to restrict analysis to the cell footprint. Image analysis was performed using a custom pipeline available at: https://github.com/harmanea/AAK1_in_cell_migration. The Pearson correlation coefficient was measured across the entire cell to assess the linear correlation between EGFP-CLCa and mScarlet channels. To segment CCPs, adaptive local thresholding was applied using a 25×25 pixel Gaussian window, followed by a morphological opening operation with a 5×5 pixel circular element. This CCP mask was then used to compute both the Manders colocalization coefficient and the ratio of mean fluorescence intensities inside versus outside the mask.

### Quantitative analysis of FA lifetime and density in RPE^WT^ and RPE^AAK1-/-^ cells

RPE^WT^ and RPE^AAK1-/-^ cells expressing mNeonGreen-paxillin were seeded onto collagen-coated coverslips and cultured for ON to allow steady state cell adhesion. TIRFM was then performed over a 5 h period, with images captured every 3 min. Image analysis was performed using a custom pipeline available at: https://github.com/harmanea/AAK1_in_cell_migration. To track the FA, the acquisitions were first denoised by Gaussian filtering with a sigma of 1. Segmentation was performed with a combination of adaptive local thresholding in a 25×25 pixel Gaussian window and global triangle method thresholding, followed by a morphological opening operation with a 3×3 pixel circular element. Individual instances in the segmentation mask were identified using the Spaghetti labeling algorithm^75^. To filter out small noisy masks, instances with an area of less than 100 pixels were omitted. Detections in neighboring frames were linked by selecting pairs with the highest intersection over union of their masks. FA lifetimes were computed only for those that both appeared and disappeared within the duration of the acquisition (i.e. they were not detected in the first or last frame). FAs with lifetimes of less than 15 min were excluded from the analysis as they mostly belonged to noisy spurious detections^34^. The FA density in these cell lines fixed with 4% PFA was calculated by simply dividing the total number of segmented instances by the area of the corresponding cell mask.

### Lifetime-aligned intensity profiling of AAK1 and PDLIM5 in FAs

Lifetime-aligned fluorescence intensity profiling of AAK1 and PDLIM5 during FA turnover was performed using live-cell time-lapse TIRFM movies acquired every 3 min over 5 h as cells spread on coverslips coated with rat tail collagen I (Gibco A10483-01; 100 µg/mL in 20 mM acetic acid; coverlisps coated for 30 min at 37 °C then washed 1x with PBS). RPE cells co-expressing mScarlet–AAK1, HaloTag–PDLIM5 (labeled ON with JF650 HaloTag ligand), and mNeonGreen–paxillin were seeded and centrifuged at 10 g for 10 min to position cells near the coverslip surface prior to imaging. FAs were segmented as described above in the section Quantitative analysis of FAs lifetime, with adaptive local thresholding, morphological opening, instance labeling, and frame-to-frame linking by maximal intersection-over-union. Only valid tracks that both appeared and disappeared within the imaging time window (i.e., absent from the first and last frame) were included. Using these criteria, 823 AAK1/PDLIM5-positive FA were analyzed. For each tracked FA, mean fluorescence intensity was extracted from the corresponding segmented FA mask in mScarlet-AAK1 and PDLIM5-HaloTag JFX 650 channels at each time point. Individual FA trajectories were aligned by their appearance and disappearance and rescaled to a normalized lifetime x-axis(0–1), and their intensity was min–max normalized(0–1). Normalized intensity traces were averaged across all FAs to generate the lifetime-aligned mean profiles shown in Fig. 6C, with the corresponding paxillin profile overlaid as a reference for FA assembly and disassembly timing. Derivatives were obtained using a discrete first-difference operator on the averaged traces, no additional smoothing was applied beyond the averaging across FAs.

### Lifetime-aligned intensity profiling of PDLIM5 and Talin1 and phospho-null mutants in FAs

Lifetime-aligned fluorescence intensity profiling of PDLIM5 and Talin1 during FA turnover was performed using live-cell time-lapse TIRFM in cells co-expressing mRuby2-paxillin as the FA marker together with either PDLIM5-HaloTag (WT or T290A; labeled with JFX650 HaloTag ligand) or mEmerald–Talin1 (WT or T2270A). For Talin1 tracking, cells were transiently transfected 36 h prior to imaging, then reseeded onto collagen-coated coverslips and cultured ON before imaging the next day. FAs were segmented and tracked in the paxillin channel using the workflow described above. In total, >1,500 PDLIM5- or Talin1-positive FAs were analyzed across three independent biological replicates.

### Quantification of PDLIM5 localization and distribution relative to F-actin and paxillin

RPE^WT^ cells co-expressing mNeonGreen-paxillin and either PDLIM5^WT^ or PDLIM5^T290D^ were seeded onto collagen-coated coverslips and grown ON in complete medium with JF552 HaloTag dye. The following day, cells were washed with PBS and fixed using a simultaneous fixation and permeabilization with 2% PFA and 0.5% Triton X-100 in PBS for 2 min, followed by fixation with 4% PFA for an additional 20 min. After fixation, cells were washed with PBS and blocked for 1 h in blocking solution (PBS containing 0.01% saponin, 3% BSA, and 0.1% lysine, pH 7.4). F-actin was stained by incubating the cells with Phalloidin 647 conjugate at 1:50 dilution (Invitrogen A22287) for 2 h and washed with PBS. For segmentation and quantification of PDLIM5 signal relative to both F-actin and paxillin, images were collected from three independent biological replicates (WT: *n* = 26 cells, T290D: *n* = 21 cells). Segmentation of FA and SF was performed as described above, omitting the denoising step, and using a larger 51×51 pixel window for the adaptive thresholding.

### FRAP

FRAP experiments were performed to measure FA protein exchange kinetics in live cells expressing either mNeonGreen–paxillin (in RPE^WT^ and RPE^AAK1-/-^ cell lines) or HaloTag-fused PDLIM5 (PDLIM5^WT^ or PDLIM5^T290D^) labeled with JF503 HaloTag dye ON. Cells were plated onto collagen-coated glass coverslips and cultured for 24 h, then switched to CO₂-independent imaging medium (FluoroBrite DMEM supplemented with 4 mM L-glutamine, 50 mM HEPES, 10% FBS). Live-cell imaging was conducted on a Zeiss LSM980 confocal microscope equipped with a 63× Plan-Apochromat objective at 37 °C. Regions of interest (ROIs) were manually drawn in ZEN Black software around individual FAs (typically 5 per cell) for photobleaching; separate reference regions and background regions were also defined for intensity normalization. Two pre-bleach images were captured to establish baseline fluorescence intensities. Photobleaching was performed using a 4 s pulse of a 488 nm laser, reducing fluorescence intensity by approximately 50–75%. Fluorescence recovery was monitored at 1-second intervals for a total of 150 s. Image series were analyzed using Zeiss ZEN Black software, applying background subtraction and correcting for incidental bleaching during image acquisition. Normalized recovery curves were then fitted to a double-exponential recovery model to determine the fluorescence recovery rate constant (k₁, k_2_) and mobile fractions. The double-exponential model was chosen based on previous reports indicating multiple kinetic subpopulations in actin-associated proteins^35,76,77^. Each condition was analyzed in at least three independent experiments, with more than 20 cells per condition and 5 FAs per cell.

### PDZ-PDLIM5 expression and purification

The GST-PDZ domain of PDLIM5 (residues 1–80) was expressed in *E. coli* BL21(DE3) cells using the pGEX-6P-1 vector. For purification, the cell pellets were resuspended in lysis buffer containing 20 mM Tris-HCl, pH 7.5, 200 mM NaCl, 2 mM BME, cOmplete mini EDTA-free protease inhibitor (Roche) and Benzonase, and lysed using a sonication. The lysate was clarified by centrifugation at 40,000 g for 30 min at 4 °C. The supernatant was incubated with Glutathione Sepharose 4 Fast Flow resin (Cytiva) for affinity purification in buffer containing 20 mM Tris-HCl, pH 7.5, 200 mM NaCl and 2 mM BME. After binding, the GST tag was cleaved by ON treatment with 3 C protease at 10 °C. The cleaved PDZ domain was further purified by SEC using a HiLoad 75 16/600 column (Cytiva) equilibrated with 20 mM Tris-HCl, pH 7.5, 200 mM NaCl, and 2 mM BME. Fractions were collected and analyzed by SDS-PAGE. Pooled fractions were concentrated, supplemented with 20% glycerol, snap-frozen in liquid nitrogen, and stored at −80 °C.

### Crystallization, Synchrotron data collection and structure determination of DQLIDL in complex with PDZ-PDLIM5

Before crystallization trials, the PDZ-PDLIM5 domain was further purified using SEC on a Superdex 75 Increase 10/300 GL column (Cytiva) in SEC buffer (20 mM Hepes, pH 7.5, 150 mM NaCl, 0.5 mM TCEP). The purified PDZ domain was concentrated to 10 mg/ml by using Amicon Ultra-15 3000 MWCO filter (Millipore) and 200 µl of the PDZ domain was supplemented with 3.5 mg of the AAK1 peptide DQLIDL. The crystals grew in a sitting drop that was prepared by mixing 400 nl of the protein solution and 200 nl of the well solution (0.2 M NaCl, 2.0 M (NH_4_)_2_SO_4_, 0.1 M C_2_H_6_AsNaO_2_, pH 6.5). The data were collected at 100 K on the beamline 14.1 at the BESSY synchrotron^78^. The crystals belonged to the orthorhombic P2_1_2_1_2_1_ spacegroup and diffracted to 1 Å resolution. The structure was solved by molecular replacement using another structure of the PDZ-PDLIM5 domain (PDB code: 2uzc;. The structure was refined using Coot^79^ and Phenix^80^ to good geometry and R-factors (Supplementary Table 5).

### PDLIM5 expression and purification

The cDNA encoding His6-tagged PDLIM5 within the pSUMO3 vector, a gift from Dr. Fiona Karet^81^, was expressed in *E. coli* BL21(DE3) cells. After harvesting, the cell pellet was resuspended in lysis buffer (50 mM Tris-HCl, pH 7.5, 150 mM NaCl, 5 mM BME, 10 mM Imidazole, cOmplete mini EDTA-free protease inhibitor and Benzonase). Cell disruption was performed by sonication, followed by centrifugation at 40,000 g for 30 min at 4 °C. The supernatant was applied to a HisCube Ni-INDIGO agarose resin (Cube Biotech) column for affinity purification. The protein was washed (50 mM Tris-HCl, pH 7.5, 150 mM NaCl, 5 mM BME, and 50 mM Imidazole) eluted (50 mM Tris-HCl, pH 7.5, 150 mM NaCl, 5 mM BME, and 250 mM Imidazole) and dialyzed ON at 10 °C against 50 mM Tris-HCl, pH 7.5, 150 mM NaCl, and 2 mM DTT. SUMO protease was added to cleave the His6-SUMO3 tag during dialysis. The cleavage reaction was completed at 30 °C for 1 h, followed by RT incubation for an additional hour. The cleaved protein was further purified using a Superdex 200 Increase 10/300 column (Cytiva) with a mobile phase of 50 mM Tris, pH 7.5, 150 mM NaCl, 10% glycerol, and 2 mM DTT. Fractions were collected and analyzed by SDS-PAGE with Coomassie staining, followed by western blotting (with antibodies specified in Supplementary Table 7, dilution 1:1000) and MS. Pooled fractions were supplemented with 20% glycerol, snap-frozen in liquid nitrogen, and stored at −80 °C.

### AAK1 and BMP2K kinase domain expression and purification

His6 tagged AAK1 (27–365) construct in the pNIC-CTH0 vector and BMP2K (38–345, K320A, K321A) construct in the pNIC-ZB vector, both obtained from Jon Elkins and previously published^4^, were expressed in BL21(DE3) cells. For purification, cell pellets were lysed in buffer (50 mM HEPES, pH 7.5, 500 mM NaCl, 5 mM Imidazole, 5% glycerol, 0.5 mM TCEP, cOmplete mini EDTA-free protease inhibitor and Benzonase) followed by sonication and centrifugation at 40,000 g for 30 min a 4 °C. The supernatant was purified using affinity chromatography with HisCube Ni-INDIGO agarose resin (Cube Biotech) washed (50 mM HEPES, pH 7.5, 500 mM NaCl, 50 mM Imidazole, 5% glycerol, 0.5 mM TCEP) and eluted with buffer containing 250 mM Imidazole. Protein was dialyzed ON at 10 °C against 50 mM HEPES, pH 7.5, 500 mM NaCl, 5% glycerol, 0.5 mM TCEP. His6 tag was cleaved during dialysis using TEV protease and removed from protein sample by reverse Ni-NTA (HisCube Ni-INDIGO agarose resin, Cube Biotech). Final purification was performed using SEC with Superdex 200 Increase 10/300 (Cytiva) in buffer containing 50 mM HEPES, pH 7.5, 500 mM NaCl, 5% glycerol, 0.5 mM TCEP and purified fractions were supplemented with 20% glycerol, snap-frozen in liquid nitrogen, and stored at −80 °C.

### Biolayer interferometry

BLI measurements were performed using a ForteBio Octet RED96e instrument. The biotinylated SSESSLPNLARSLLLVDQLIDL peptide and a scrambled sequence negative control peptide were loaded onto High Precision Streptavidin 2.0 sensors (SAX2, Sartorius) by immersion for 600 s in loading buffer (20 mM PBS, pH 7.5) containing 20 μg/ml biotinylated peptide. To compensate the potential nonspecific interactions of PDZ-PDLIM5 with the sensors, biotinylated control peptide-loaded SAX2 sensors were used as blank. The binding assays were conducted at 25 °C with a 5 Hz acquisition rate and a sample plate shake speed of 1000 rpm. Both active and blank sensors were used in parallel following this procedure: 120 s baseline in the assay buffer (20 mM Tris-HCl, pH 7.5, 200 mM NaCl, 2 mM DTT, 0.005% Tween 20, 600 seconds association in the assay buffer containing increasing concentrations of PDZ-PDLIM5 (5-points of 3-fold dilution series ranging from 1.71 μM to 137 μM for PDZ-PDLIM5) and 300 seconds dissociation in the assay buffer. Before the first measurement cycle and after each following cycle, three repetitions of 30 seconds regeneration in 50 mM NaOH followed by 30 s wash with assay buffer were performed to regenerate the sensor surface. The obtained data were processed using ForteBio Data Analysis 11.1 software. After the blank subtraction and alignment of baselines, the resulting sensorgrams were analyzed using steady-state analysis (averaging signal of 5 s window starting 10 s before the end of association phase) fitting with 1:1 binding model. The K_d_ values determined as an average of three independent measurements.

### Reporting summary

Further information on research design is available in the Nature Portfolio Reporting Summary linked to this article.

## Supplementary information

Supplementary Information

Description of Additional Supplementary Information

Supplementary Data 1

Supplementary Data 2

Supplementary Data 3

Supplementary Data 4

Supplementary Movie 1

Supplementary Movie 2

Supplementary Movie 3

Reporting Summary

Transparent Peer Review file

## Source data

Source data

## Data Availability

The data sets generated during this study are available as follows: Structures coordinates have been deposited on the PDB: with the accession 9F6S for the complex of PDZ-PDLIM5 in complex with VDQLIDL. The MS DDA and DIA data have been deposited to the ProteomeXchange Consortium via the PRIDE^82^ partner repository with the dataset identifiers: 5488: PXD055851. 5235: PXD055831. 5295: PXD055980. 5816: PXD055873. 5489: PXD056027. 5563: PXD056013. 5880: PXD055912. The MS PRM data have been deposited to Panorama Public and are available at: https://panoramaweb.org/AAK1-BMP2K-substrates.url. Source data are provided with this manuscript. The raw live-cell imaging and immunofluorescence datasets generated in this study are available from the corresponding author upon request. Source data are provided with this paper.

## Code availability

Python code created by Adam Harmanec for fixed cell or live-cell quantitative TIRFM analysis is available on Github: https://github.com/harmanea/AAK1_in_cell_migration Code for in-silico screen for [L/I]XXQXTG created by Stephanie J Spielman is available on Github: https://github.com/sjspielman/endocytosis-slims-screen and the resulting CSV file which is in the repository as Supplementary Data 1: https://github.com/sjspielman/endocytosis-slims-screen/blob/main/results/motif-results.csv.
