# Supplementary material for: AAK1-mediated phosphorylation of PDLIM5 and Talin1 promotes focal adhesion disassembly to accelerate cell migration
Source: Nat Commun. 2026 May 4;17:6023. doi: 10.1038/s41467-026-72501-w (PMC13346677; doi:10.1038/s41467-026-72501-w)
Supplement: Supplementary file 1 — Supplementary Information [file 41467_2026_72501_MOESM1_ESM.pdf]

Supplementary Methods

Mass spectrometry experiments and downstream data analyses

Co-IP of endogenous AAK1 under steady state conditions using DIA (5295, Fig. 5A)

For the co-IP of endogenous AAK1 RPE^{WT} and $RPE^{AAK1-/-}$ cells were grown ON on collagen I coated plates (Rat tail, Gibco, A10483-01; 100 ug/mL in 20 mM acetic acid, coating for 30 min at 37°C) and washed once with PBS. $RPE^{AAK1-/-}$ cell line was used as a negative control to account for non-specific interactions of the antibody and beads. Total 2 mg protein was extracted from RPE^{WT} and $RPE^{AAK1-/-}$ cells using a lysis buffer containing 50 mM HEPES, 1 mM EGTA, 0.5% NP-40, 1 mM MgCl2, 200 mM NaCl, and protease inhibitors (Roche). For the RPE^{WT}, 4 replicates were processed from individual cell cultures. For $RPE^{AAK1-/-}$, 6 replicates were processed from individual cell cultures. Post-homogenization, insoluble fractions were separated by centrifugation (10 000×g for 20 min at 8 °C). In this and all subsequent experiments, protein concentrations were quantified via Tryptophan assay (excitation: 295 nm, emission: 350 nm). AAK1 was immunoprecipitated ON at 4 °C using 60 µl A/G Agarose beads (Pierce™ Protein A/G Agarose, 20422) per sample and 20 µg of a custom-made rabbit polyclonal antibody targeting the N-terminus of AAK1, produced by Eurogentec using a peptide with the sequence KKFFDSRREQGGSGLGSGSSGGGGSTSGL, followed by cross-affinity purification with their custom polyclonal antibody service. The beads were subsequently washed 4 times in a buffer containing 50 mM HEPES, 1 mM EGTA, 1 mM MgCl2, 200 mM NaCl. The buffer was then replaced with 8 M urea in Tris-HCl. The immunoprecipitate was reduced using 10 mM DTT (Merck), alkylated with 40 mM IAA (ThermoFisher), and subsequently quenched with 20 mM DTT. Digestion was performed using a Trypsin-Lys C (Promega) mixture at a 50:1 protein:protease ratio for 3 hours at 37°C. This was followed by dilution to 1M urea using 50 mM Tris, pH 8 and an ON digestion with SOLu-Trypsin (Merck, EMS0004) at the same protein:protease ratio. The reaction was stopped with 1% TFA (Honeywell), and the insoluble fraction was separated by centrifugation (4 700×g for 10 min). SDB–RPS stage tips were used for desalting. The peptides were then concentrated in a SpeedVac concentrator (Thermo Fisher Scientific).

Peptides were extracted into LC-MS vials by 2.5% formic acid in 50% ACN, followed by 100% ACN with the addition of polyethylene glycol (final concentration 0.001%) and concentrated in a SpeedVac concentrator. Peptide concentration was estimated using small-aliquot injections analyzed on an UltiMate 3000 RSLCnano system (Thermo Fisher Scientific) online connected to Impact II system (Bruker) using the area under the total ion chromatogram curve as a total peptide quantity estimate and using external calibration curve done using HeLa solutions (Pierce, 88329).

LC-MS/MS analyses of the peptide mixtures were done using UltiMate 3000 RSLCnano system connected to timsTOF Pro 2 mass spectrometer (Bruker). Prior to LC separation, tryptic digests were online concentrated and desalted using a trapping column (Acclaim PepMap 100 C18, dimensions 300 µm ID, 5 mm long, 5 µm particles, Thermo Fisher Scientific). After washing the trapping column with 0.1% formic acid, the peptides were eluted (flow rate 200 nl/min) from the trapping column onto an analytical column (Aurora C18, 75 µm ID, 250 mm long, 1.6 µm particles, Ion Opticks, PN AUR2-25075C18A-CS) by 60 min linear gradient program (3-42% of mobile phase B; mobile phase A: 0.1% formic acid in water; mobile phase B: 0.1% formic acid in 80% ACN). Equilibration of the trapping and analytical column was done before sample injection into the sample loop. The analytical column was placed inside the Column Toaster heater (Bruker) and its emitter side was installed inside the CaptiveSpray ion

source (Bruker) according to the manufacturer's instructions with the column temperature set to 50 ºC.
MS data were acquired in DIA mode with base method m/z range of 100-1700 and 1/k0 range of 0.6-1.6 V×s×cm-2 covering m/z 400-1000 precursor range with equal windows size of 25 Th using two steps for each PASEF scan (Data S4) and cycle time of 100ms locked to 100% duty cycle.
DIA data were processed in DIA-NN (version 1.8.1) in library-free mode against the modified cRAP database (based on http://www.thegpm.org/crap; 111 sequences in total) and UniProtKB protein database for Homo sapiens (https://www.uniprot.org/proteomes/UP000005640); version 2023/06, number of protein sequences: 20,586). During library preparation, the following parameters were applied: no variable modifications; carbamidomethylation set as a fixed modification; trypsin/P specified as the enzyme with up to one missed cleavage; and a peptide length range of 7–30 amino acids. FDR control was set to 1%. MS1 and MS2 accuracies as well as scan window parameters were set based on the initial test searches (median value from all samples ascertained parameter values). MBR was switched on.
The main DIA-NN report was further processed using the software container environment (https://github.com/OmicsWorkflows), version 4.7.7a. Processing workflow is available upon request. It covered: a) removal of low-quality (Global.Q.Value or Global.PG.Q.Value >0.01) and cRAP associated precursors, b) filtering out proteins not quantified in at least 50% samples, c) normalized PG.Quantity intensities log2 transformation and normalization using loessF algorithm, d) imputation of the missing values on the loessF normalized PG.Quantity intensities level using 0.001 quantile value e) differential expression analysis using LIMMA statistical test.

For the identification of the AAK1 peptides belonging to the canonical isoform (Uniprot ID Q2M2I8-1, Ensembl transcript ID ENST00000409085.9) and the long isoform (Uniprot ID A0A096LP25, Ensembl transcript ID ENST00000606389.8), the data was processed in DIANN with the same parameters as above but with the FASTA complemented with the sequence for the long isoform. The data was then manually searched for the peptides belonging to the diverging sequences of these two isoforms.

Context dependent interactome analysis of endogenous AAK1 in RPE^{WT} cells cultured on collagen or PLL using DIA (5816, Fig. 5H)

Sample preparation
For the differential AAK1 co-IP, RPE^{WT} cells were seeded on 15 cm cell culture plates coated with either rat tail collagen I (Rat tail, Gibco, A10483-01; 100 ug/mL in 20 mM acetic acid) or 0.01% PLL (Merck, P8920; prepared by diluting a 0.1% stock in dH20) for 30 min at 37°C and washed once with PBS. Two hours post-seeding, total protein was extracted from the cells using a buffer containing 150 mM NaCl, 10 mM MgCl2, 1 mM EGTA, 1% glycerol, 20mM HEPES and 1% digitonin. The experimental design is visualized in Fig. 5G.
Post-homogenization, insoluble fractions were separated by centrifugation (10 000×g for 20 min at 8 °C). Endogenous AAK1 was immunoprecipitated ON at 4 °C from 3.5 mg protein per sample using 60 µl A/G Agarose beads (ThermoFisher) per sample and 20 µg of the previously mentioned custom-made anti-AAK1 antibody. The beads were subsequently washed 4 times in a buffer containing NaCl 150 mM, MgCl2 10 mM, 1 mM EGTA, 1% glycerol, 20 mM HEPES. Sample preparation procedures were identical to those used for experiment 5295.
LC-MS/MS analyses of the samples were done using UltiMate 3000 RSLCnano system (Thermo Fisher Scientific) connected to timsTOF Pro mass spectrometer (Bruker). Prior to LC separation, tryptic digests were online concentrated, desalted and eluted identically as in

experiment 5295. Equilibration of the trapping and analytical column was done before sample injection into the sample loop.
MS data were acquired in DIA mode with base method m/z range of 100-1700 and 1/k0 range of 0.6-1.4 V×s×cm-2 covering m/z 400-1000 precursor range with equal windows size of 25 Th using two steps for each PASEF scan (Data S4) and cycle time of 100 ms locked to 100% duty cycle.
DIA data were processed as in experiment 5295, using MaxLFQ intensities for the statistical analysis.

Pulldown-based characterization of interactors of the AAK1 C-terminus using DIA and RPE^{WT} cell lysates (5489 and 5563, Fig. 7B)

For the C-terminal co-IP, total protein was extracted from RPE^{WT} cells grown ON on collagen I using a buffer containing NaCl 150 mM, MgCl2 10 mM, 1 mM EGTA, 1% glycerol, 20 mM HEPES and 1% digitonin and protease inhibitors (Roche). Three biological replicates, each from separate cultures, were processed. Post-homogenization, insoluble fractions were separated by centrifugation (10 000×g for 20 min at 8 °C). AAK1 C-terminal interactome was pulled down using 9 µg of biotinylated C-terminal AAK1 peptide SSESSLPNLARSLLLVDQLIDL (Genscript) and 60 µl Streptavidin beads (ThermoFisher) per sample, incubated ON at 4 °C.
The beads were subsequently washed twice in a buffer containing 150 mM NaCl, 10 mM MgCl2, 1 mM EGTA, 1% glycerol, 20 mM HEPES and 0.5% digitonin and three-times in the same buffer without digitonin. Sample preparation procedures were identical to those used for 5295.
Initially, the first replicate samples were prepared and processed (5489) followed by 2nd and 3rd replicate preparation and processing (5563). The data from both separate experiments were combined on protein level, see below for more details.
LC-MS/MS analyses of the samples (200ng per injection) were done using UltiMate 3000 RSLCnano system (Thermo Fisher Scientific) connected to timsTOF Pro 2 mass spectrometer (Bruker). Prior to LC separation, tryptic digests were online concentrated, desalted and eluted identically as in experiment 5295. Equilibration of the trapping and analytical column was done before sample injection into the sample loop.
MS data were acquired in DIA mode with base method m/z range of 100-1700 and 1/k0 range of 0.6-1.6 V×s×cm-2 covering m/z 400-1000 precursor range with equal windows size of 25 Th using two steps for each PASEF scan (Data S4) and cycle time of 100 ms locked to 100% duty cycle.
DIA data were processed in DIA-NN as described for experiment 5295.
The main DIA-NN report was further processed using the software container environment (https://github.com/OmicsWorkflows), version 4.7.7a. Processing workflow is available upon request. It covered removal of low-quality (Global.Q.Value or Global.PG.Q.Value >0.01) and cRAP associated precursors and filtering out precursors not quantified in all replicates of at least one sample type. The data was then exported and PG.MaxLFQ intensities were further analyzed by calculating ratios of sample/control for each experiment followed by averaging.

Comparative interactome profiling of PDLIM5 in RPE^{WT} and $RPE^{AAK1-/-}$ using DIA (5880, Fig. 4E)
For endogenous PDLIM5 co-IP, total protein was extracted from RPE^{WT} and $RPE^{AAK1-/-}$ cells grown ON on plates coated with collagen I. Lysis buffer contained 150 mM NaCl, 10 mM MgCl2, 1 mM EGTA, 1% glycerol, 20 mM HEPES and 1% digitonin and protease inhibitors (Roche). Four biological replicates per condition, each from separate cultures, were processed. Post-homogenization, insoluble fractions were separated by centrifugation (10 000×g for 20

min at 8 °C). PDLIM5 was immunoprecipitated using 25 µg of anti-PDLIM5 antibody A14933 (Antibodies.com) and 60 µl A/G Agarose beads (ThermoFisher) per sample, incubated ON at 4 °C.

The beads were subsequently washed 4 times in a buffer containing NaCl 150 mM, MgCl2 10 mM, 1 mM EGTA, 1% glycerol, 20 mM HEPES. The following procedures are identical to those used for 5295.

LC-MS/MS analyses of the samples were done using UltiMate 3000 RSLCnano system (Thermo Fisher Scientific) connected to timsTOF Pro mass spectrometer (Bruker). Prior to LC separation, tryptic digests were online concentrated, desalted and eluted identically as in experiment 5295. Equilibration of the trapping and analytical column was done before sample injection into the sample loop.

MS data were acquired in DIA mode with base method m/z range of 100-1700 and 1/k0 range of 0.6-1.4 V×s×cm-2 covering m/z 400-1000 precursor range with equal windows size of 25 Th using two steps for each PASEF scan (Data S4) and cycle time of 100 ms locked to 100% duty cycle.

The main DIA-NN report was further processed using the software container environment (https://github.com/OmicsWorkflows), version 4.7.7a. Processing workflow is available upon request. Briefly, it covered: a) removal of low-quality (Global.Q.Value or Global.PG.Q.Value >0.01) and cRAP associated precursors, b) filtering out precursors not quantified in all replicates of at least one sample type, c) normalized precursor intensities log2 transformation and normalization using loessF algorithm, d) imputation of the missing values on the loessF normalized precursor intensities level random distribution data (d-value 1.8, w-value 0.3), e) back log transformation of imputed precursor intensities, f) PG.MaxLFQ protein intensities calculation using using the iq R package on back log2 transformed precursor intensities from the step g) log2 transformation of PG.MaxLFQ protein intensities, h) differential expression analysis using LIMMA statistical test.

Downstream data analyses

For the analyses of the PRM measurements of endogenous PDLIM5 and Talin1 phosphopeptides to assess their abundance in RPE^{WT} and $RPE^{AAK1/BMP2K-/-}$, the intensities of the fragments with the highest peaks from two individual experiments of 7 replicates total were normalized to the lowest value within each experiment and pooled together. For all statistical analyses of the PRM data, an unpaired t-test was used.

For the GO analyses of the global proteome profiling, proteins with significant change in abundance - adjusted p-value < 0.05 and a log2 fold change over 0.5 - were used as input for the GO analysis and visualization of the experiment using genetic perturbation. For the analysis of the experiment with pharmacological perturbation, a log2 fold change cutoff of 0.3 was used, as the overall number of dysregulated proteins was lower than upon genetic perturbation (Fig. 2A, C, Fig. S4).

For the calculation of the intersection of proteins upregulated upon perturbations (Supplementary Fig. 6A, left), significantly upregulated proteins (adjusted $p < 0.05$) were selected. Overlap was quantified as intersection-over-union, $|A \cap B| / |A \cup B|$, and reported as a percentage.

For phosphoproteome GO analyses, TiO_2 and IMAC enrichment datasets were combined. To isolate phosphorylation changes from protein abundance effects, phosphoproteins with significant changes at the total-protein level (adjusted $P < 0.05$; $|\log_2$ fold change$| > 0.5$ for genetic perturbation or > 0.3 for pharmacological perturbation) were excluded. GO analysis was then performed on phosphoproteins containing at least one significantly dysregulated

phosphopeptide (adjusted P < 0.05) meeting the fold-change cut-offs (|$\log_2$ fold change| > 1 for genetic perturbation; |$\log_2$ fold change| > 0.5 for pharmacological perturbation; Fig. 2B, D).
For the calculation of the intersection of phosphoproteins dysregulated upon perturbations (Fig. S6B), first, all proteins with at least one dysregulated phosphopeptide (adjusted p < 0.05) were selected, and then all proteins showing significant whole-protein changes (adjusted p < 0.05) were excluded to specifically interrogate phosphorylation regulation. Subsequently, a number of phosphoproteins shared between genetic and pharmacological perturbations were visualized as a Venn diagram (Supplementary Fig. 6A, right). GO analysis was subsequently conducted using this dataset (Supplementary Fig. 6B).
To determine the overlap of significantly enriched GO-CC terms (adjusted p < 0.05), data was obtained from Data S3, and intersections between perturbations were calculated. The resulting overlaps were displayed as Venn diagrams (Supplementary Fig. 6C).

For the visualisation of co-IP experiments in figures, $\log_2$ fold-change thresholds were selected based on expected effect sizes and background levels. For the GO analysis of AAK1 steady-state interactome compared against a bait-free control, proteins with adjusted p-value < 0.05 and logFC > 1 were used (Fig. 5A). For the GO analysis of the differential AAK1 interactome, which is a comparison between two closely related biological states, proteins with a p-value ≤ 0.05 and logFC > 0.3 were used. These were filtered for FA, endosome and actin cytoskeleton components and the previously published interactions between these proteins were sourced from STRING. The figure was then created in Cytoscape version 3.10.2 (Fig. 5H). For the GO analysis of differential PDLIM5 interactome in presence and absence of AAK1, which is a comparison between two closely related biological states, proteins with p-value < 0.05 and logFC > 0.3 were used (Fig. 4E).
For all the GO analyses, DAVID GO platform was used. Log_2 fold-change cutoffs were used only to define the subsets visualized in GO/STRING/Cytoscape summary plots. Statistical testing was performed on the full datasets, and complete result tables (including proteins below visualization thresholds) are provided in the Supplementary Datasets (Supplementary Data 2 and Supplementary Data 3).

Co-IP of PDLIM5 with AAK1 (Fig. 7C)

For PDLIM5 co-IP with AAK1, total protein was extracted from RPE^{WT} and $RPE^{AAK1-/-}$ cells using a buffer containing 50 mM HEPES, 1 mM EGTA, 0.5% NP40, 1 mM $MgCl_2$, 200 mM NaCl, and protease inhibitors (Roche). For both RPE^{WT} and $RPE^{AAK1-/-}$ conditions, three biological replicates were processed from individual cell cultures. After homogenization, insoluble fractions were separated by centrifugation at 10,000 g for 20 minutes at 8°C. Protein concentrations were determined using the BCA assay (ThermoFisher). AAK1 was immunoprecipitated for 2 hours at RT from fully confluent 15 cm dishes (one dish per sample) using 50 µl of Protein G Dynabeads (Invitrogen) and 10 µg of the previously described custom-made anti-AAK1 rabbit polyclonal antibody. The beads were then washed three times with the same buffer that was used for the lysis. Next, 7 µg of recombinant PDLIM5 protein in 1 ml of PBS was added and incubated for 2 hours at RT. The beads were then washed five times with PBS. Samples were analysed by western blotting using a 10% SDS-PAGE gel and primary anti-PDLIM5 antibody (ab196559), dilution 1:1000.

Graphs and ilustrations
The workflow and model illustrations were created using BioRender (https://biorender.com/). The consensus sequence logo was created using the ggseqlogo library in Rstudio. The dotplots

were created using the ggplot2 library in Rstudio. Rstudio version 4.3.2 was used. The bar graphs were created using GraphPad Prism (version 10.2.3).

Supplementary Figures

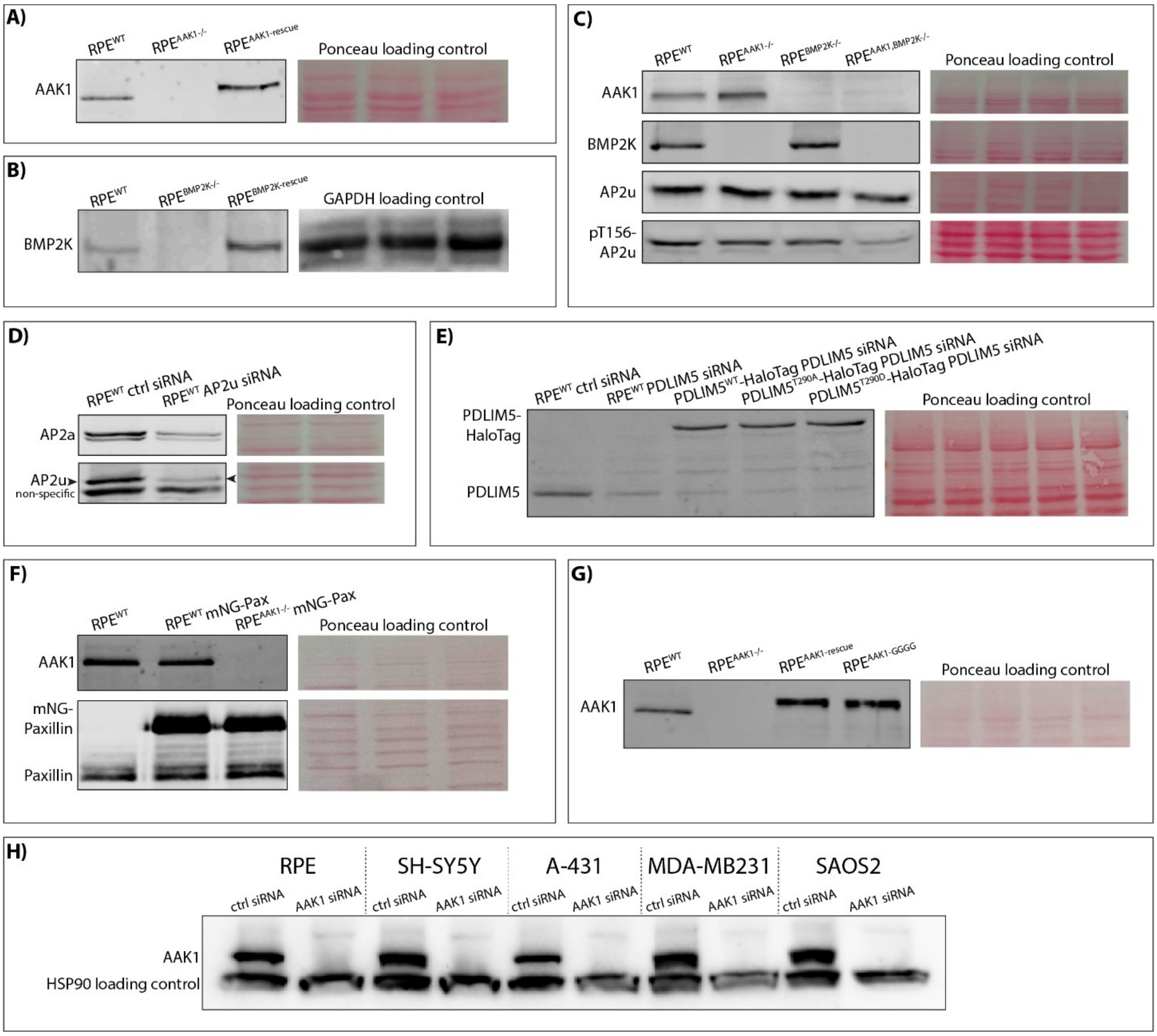

Supplementary Figure 1. Validation of engineered cell lines used in this study.
A) Validation of $RPE^{AAK1-/-}$ cell line reconstituted with mScarlet-AAK1 ($RPE^{AAK1\text{-}rescue}$), n = one independent experiment. **B)** Validation of $RPE^{BMP2K-/-}$ reconstituted with mScarlet-BMP2K ($RPE^{BMP2K\text{-}rescue}$), n = two independent experiments. **C)** Validation of single AAK1 or BMP2K knock-out cell lines and double knockout cell lines: $RPE^{AAK1-/-}$, $RPE^{BMP2K-/-}$, $RPE^{AAK1, BMP2K-/-}$, n = one independent experiment. **D)** AP2μ siRNA-mediated knockdown of AP2 complex achieves approximately 80% reduction in AP2μ and AP2α expression in RPE cells. The AP2μ-specific band is indicated with an arrow, n = one independent experiment. **E)** Validation of PDLIM5 knockdown (90% efficiency) in RPE cells using siRNA. Validation of $PDLIM5^{WT}$-HaloTag, $PDLIM5^{T290A}$-HaloTag, or $PDLIM5^{T290D}$-HaloTag **-**expressing RPE cell lines and confirmation of equal ectopic expression levels of PDLIM5 mutants, n = three independent experiments. **F)** Validation of mNeonGreen-paxillin expressing RPE^{WT} and $RPE^{AAK1-/-}$ cell lines, n = one independent experiment. **G)** Recovery of AAK1 expression in $RPE^{AAK1-/-}$ cells through reconstitution with mScarlet-AAK1 WT ($RPE^{AAK1\text{-}rescue}$) or the mScarlet-AAK1 C-terminal GGGG mutant construct ($RPE^{AAK1\text{-}GGGG}$), with equal ectopic expression levels of AAK1 in both cases, n = one independent experiment. **H)** Analysis of AAK1 knockdown efficiency (~95%) across multiple cell lines (RPE, SH-SY5Y, A431, MDA-MB-231, and

SAOS2), n = one independent experiment. Antibodies used for western blotting are listed in Table S7, 1:1000 dilution was used for all the antibodies.

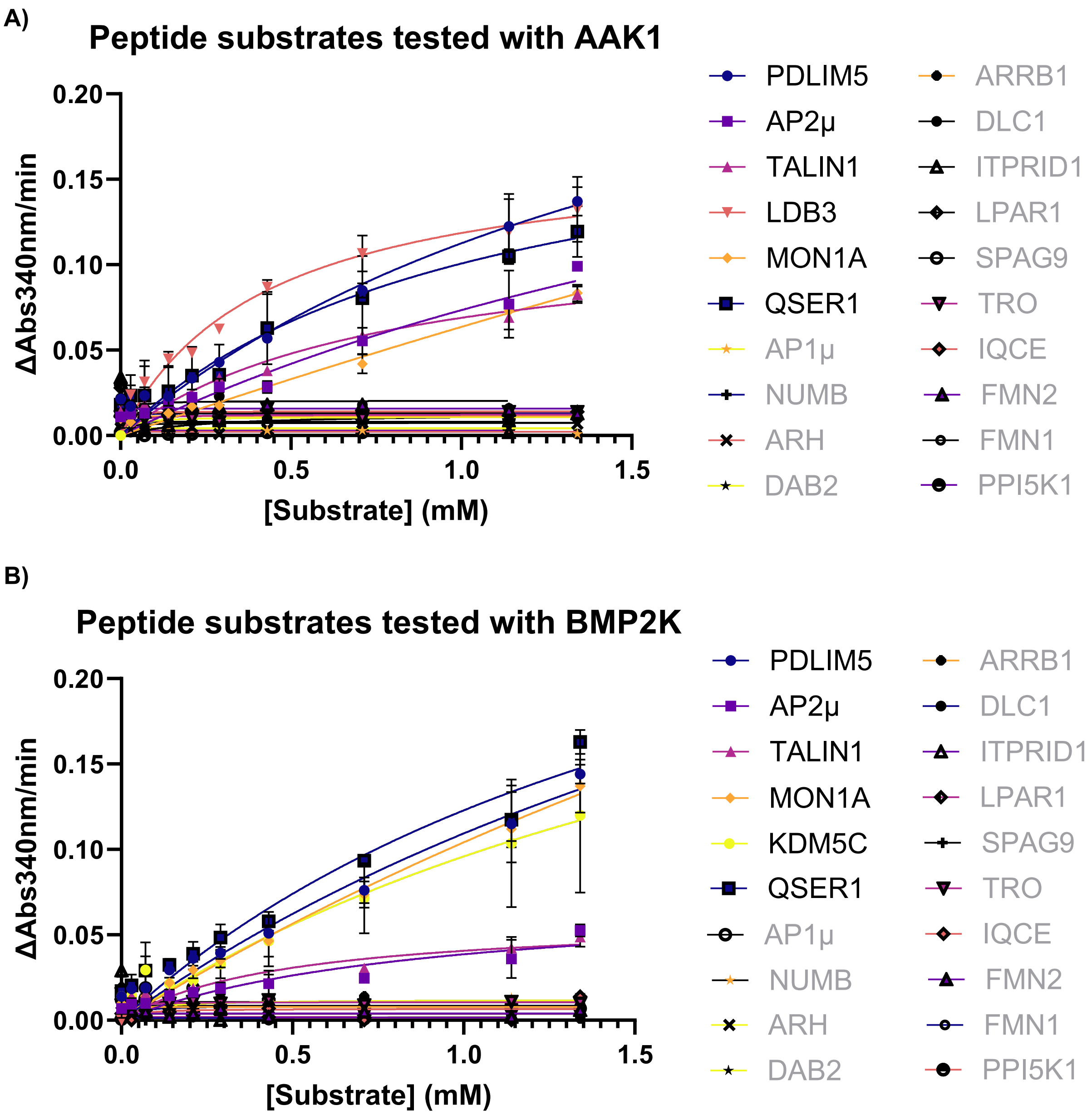

Supplementary Figure 2. Related to Figure 1. Identification of AAK1 and BMP2K substrates using NADH coupled kinase assay.

The reaction rates of AAK1 (A) and BMP2K (B) with various substrates were assessed by monitoring the decrease in NADH absorbance at 340 nm, which was used to calculate initial reaction rates across a range of substrate concentrations. Initial rates, determined at low substrate conversion, were plotted as ΔAbs340nm/min against substrate concentration and fitted with nonlinear regression curves using the Michaelis-Menten equation. PDLIM5 T290, AP2M1 T156, TALIN1 T2270, KDM5C T1385**,** and QSER1 T206 displayed clear concentration-dependent increases in reaction rates, indicating efficient phosphorylation by AAK1 or BMP2K. In contrast, non-phosphorylated peptides, represented by grey data points (e.g., ARRB1, DLC1, and others), exhibited consistent, low ΔAbs340nm/min values across all concentrations, reflecting the baseline ATPase activity of AAK1 and BMP2K independent of substrate phosphorylation. For each peptide, n = 2 independent experiments. Error bars represent mean ± SD.

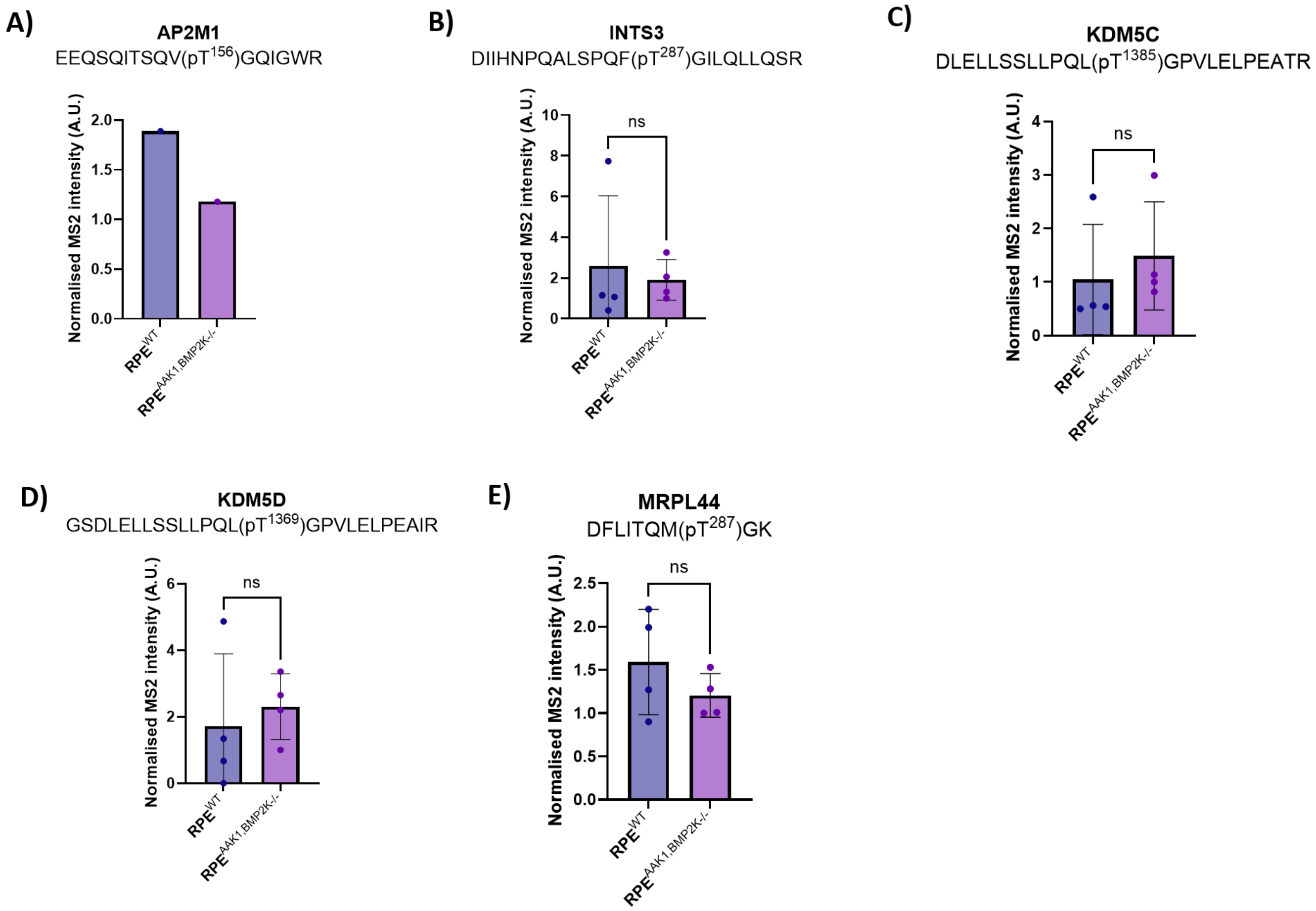

Supplementary Figure 3. Related to Figure 1.

Comparison of candidate phosphopeptide abundance between RPE^{WT} and $RPE^{AAK1,BMP2K-/-}$. **(A)** AP2µ pT156, a well-established AAK1/BMP2K substrate, is included as a positive control and shows reduced phosphorylation in the double-knockout condition**. (B-E)** Candidate phosphopeptides INTS3 pT287, KDM5C pT1385, KDM5D pT1369, and MRPL44 pT287 show no significant change between genotypes. Bars show mean ± SD, two-tailed unpaired t-test; ns, $p > 0.05$.

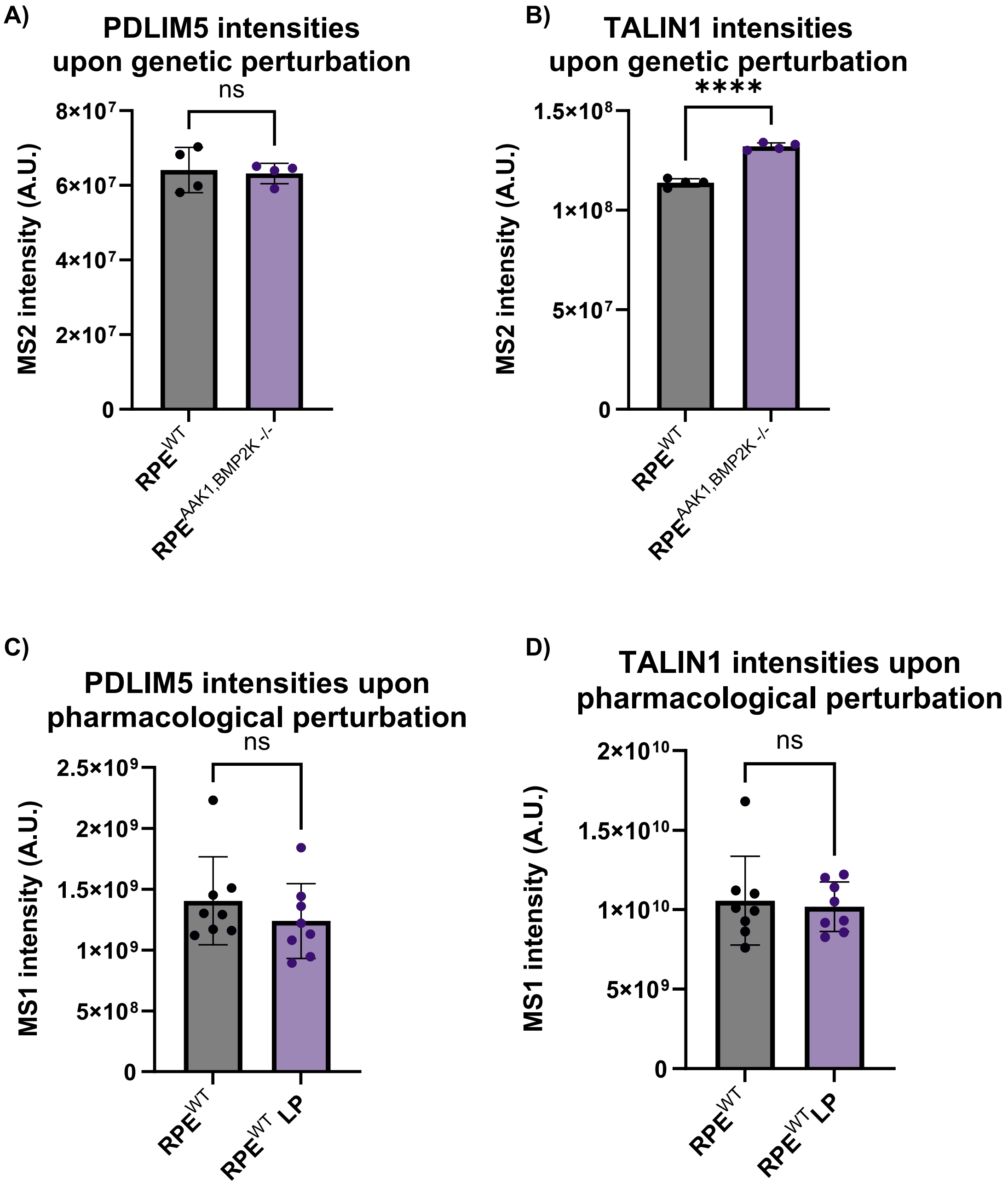

Supplementary Figure 4. Related to Figure 1. PDLIM5 and TALIN1 protein levels did not decrease upon genetic or pharmacological perturbation of AAK1 and BMP2K.
In RPE cells, the total protein abundance of PDLIM5 and TALIN1 did not decrease following genetic (**A, B**) or pharmacological perturbation (**C, D**) of AAK1 and BMP2K. For genetic perturbation, MaxLFQ MS2 intensities obtained from DIA measurements are plotted, n = four biological replicates (A, B). For pharmacological perturbation, MS1 intensities obtained from DDA measurements are plotted, n = eight biological replicates (C, D). Error bars represent the mean ± SD, unpaired t-test used for statistical analysis, p-values for A, B, C and D respectively are 0.79, <0.0001, 0.34 and 0.74.

A

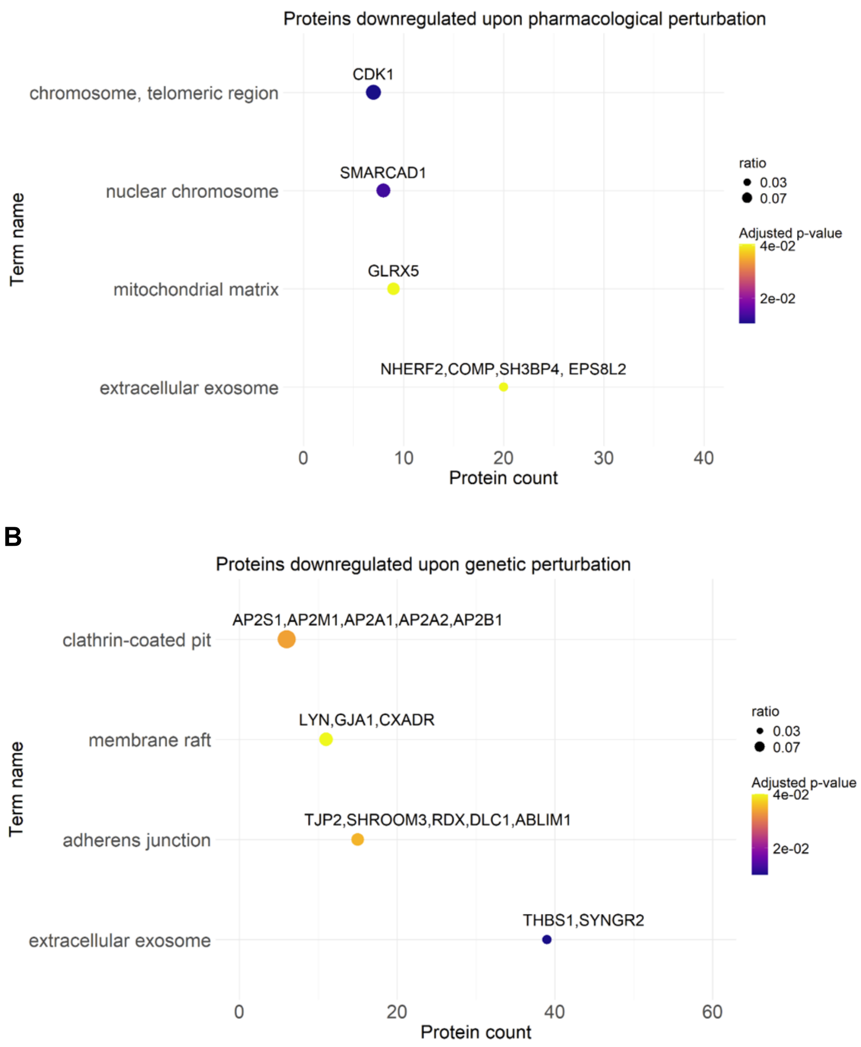

Supplementary Figure 5. Related to Figure 2.
(A) GO analysis of proteins significantly downregulated upon pharmacological perturbation of AAK1/BMP2K. Dot plots show the cellular components most enriched for proteins downregulated in RPE^{WT}LP. Only proteins downregulated with log2 fold change > 0.3 and adjusted p-value < 0.05 were used for the analysis. **(B)** GO analysis of proteins significantly downregulated following genetic perturbation of AAK1/BMP2K**.** Dot plots show the cellular components most enriched for proteins downregulated in RPE$^{AAK1/BMP2K\ -/-}$. Only proteins downregulated with log2 fold change > 0.5 and adjusted p-value < 0.05 were used for the analysis. Statistical analysis was performed using LIMMA with Benjamini-Hochberg correction.

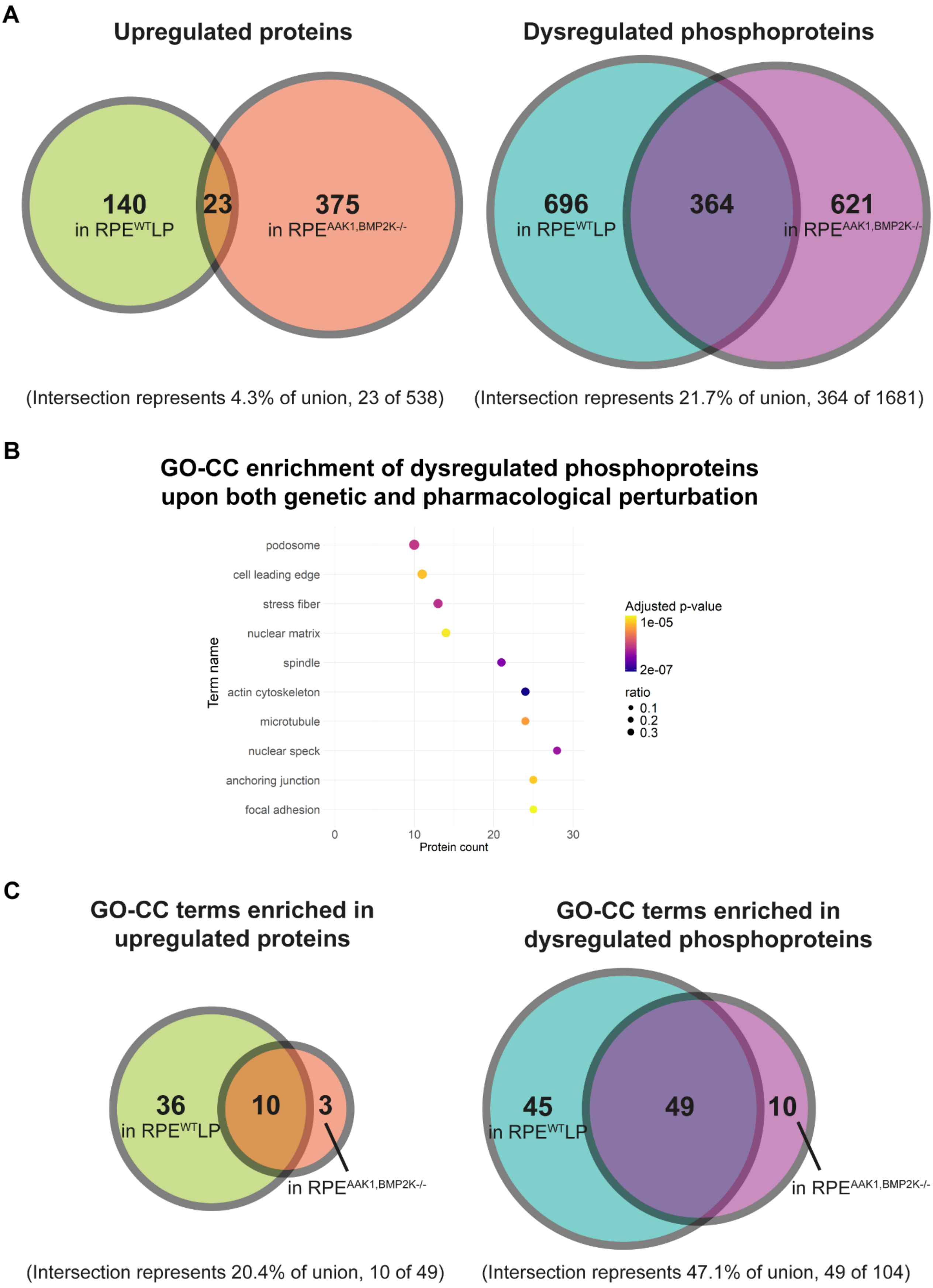

Supplementary Figure 6. Related to Figure 2.

(A) Protein-level intersection between pharmacological inhibition and genetic loss of AAK1/BMP2K. Venn diagrams summarizing intersection between pharmacological inhibition (LP-935509; 10 μM, 6 h) and AAK1/BMP2K double knockout. Numbers indicate unique/shared hits; percentages indicate intersection relative to the union. **(B)** GO analysis of the intersection of dysregulated phosphoproteins. **(C)** Venn diagrams summarizing pathway-level intersection of GO-CC enrichment comparing pharmacological inhibition versus AAK1/BMP2K double knockout. Numbers indicate unique/shared GO-CC terms; percentages indicate intersection relative to the union.

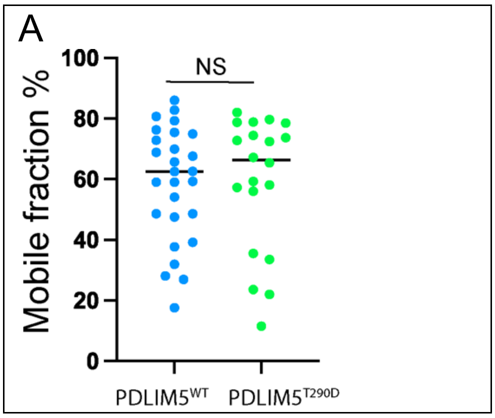

Supplementary Figure 7. Related to Figure 4.

(A) FRAP analysis comparing the percentage of mobile fractions of PDLIM5^{WT} and PDLIM^{T290D} constructs localized at FAs. Each data point represents measurements from individual FAs, averaged across 20 cells from three independent biological replicates. Statistical comparison (NS = not significant, $p > 0.05$) was performed using an unpaired two-tailed t-test. Horizontal bars indicate mean values.

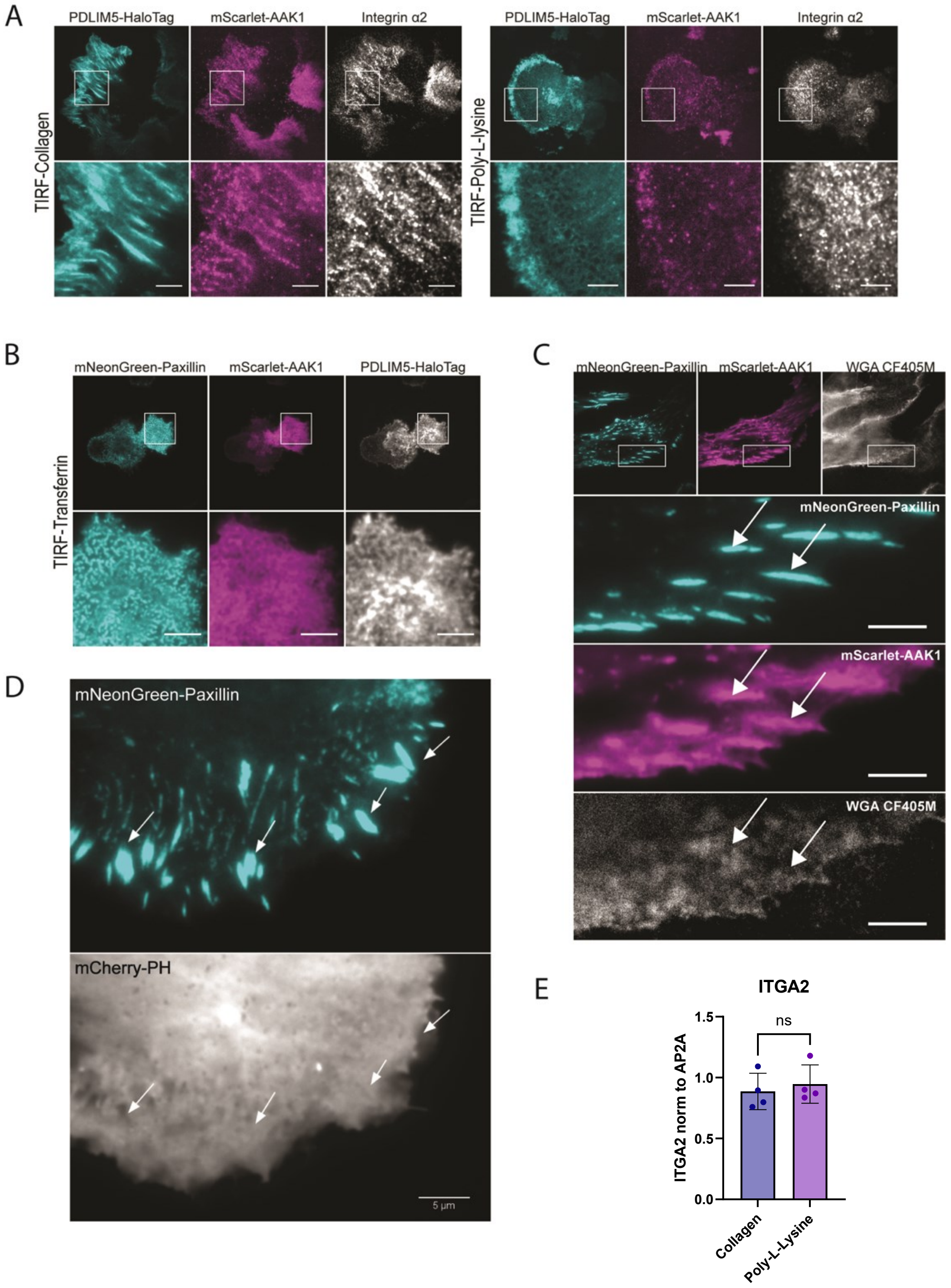

Supplementary Figure 8. Related to Figure 5.

(A) AAK1 distribution at the PM relative to PDLIM5 and Integrin α2 on collagen- (left panel) versus PLL-coated surfaces (right panel). TIRFM images show mScarlet-AAK1 localization alongside PDLIM5-HaloTag and Integrin α2 in RPE cells. **(B)** Localization of AAK1, paxillin, and PDLIM5 at the PM in RPE cells seeded on transferrin-coated surfaces (negative control). TIRFM images depict the distribution of mScarlet-AAK1 relative to mNeonGreen-paxillin and PDLIM5-HaloTag. The images reveal the absence of organized FAs in RPE cells seeded on transferrin-coated surfaces and the lack of distinct streak-like AAK1 concentration at the PM, in contrast to cells seeded on collagen-coated surfaces. **(C)** TIRFM demonstrates that non-specific PM probe WGA does not localize to FAs, supporting the regulated recruitment of AAK1. mNeonGreen-Paxillin (cyan) marks FAs, while mScarlet-AAK1 (magenta) specifically colocalizes at these sites. **(D)** mCherry-PH (gray), a PtdIns(4,5)P2 specific PM probe, displays diffuse localization and does not enrich at FAs, ruling out irregular illumination or general

membrane attachment as explanations for the AAK1 pattern. These findings confirm that AAK1 localization to FAs is driven by regulated recruitment. Scale bar: 5 µm. (A)-(D) n = three independent experiments. **(E)** Total integrin α2 protein abundance remains unchanged in cells seeded on collagen versus PLL. Western blot analysis of integrin α2 in whole-cell lysates from cells seeded on collagen or PLL for 2 h. N = four independent biological replicates. Two-tailed unpaired t-test was used for statistical analysis (ns = not significant, $p > 0.05$).

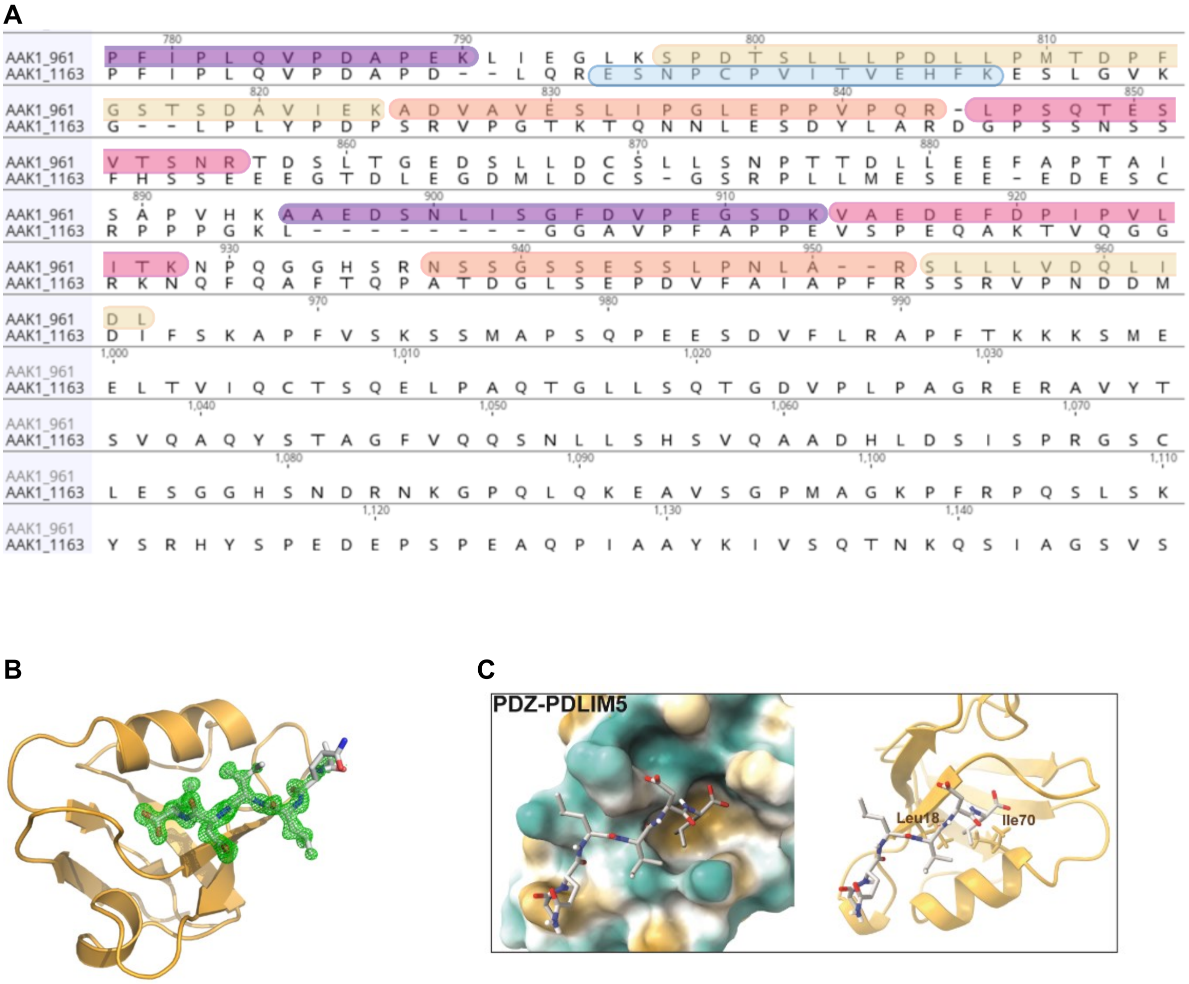

Supplementary Figure 9. Related to Figure 7.
(A) Identification of the 961-amino-acid AAK1 isoform as the predominant variant in RPE cells by LC-MS. DIA MS analysis of AAK1 immunoprecipitation identified eight tryptic peptides corresponding to the LIDL-containing AAK1 isoform (961 amino acids; Uniprot ID Q2M2I8-1, Ensembl transcript ID ENST00000409085.9). In contrast, only a single tryptic peptide unique to the longer isoform (1163 amino acids; Uniprot ID A0A096LP25, Ensembl transcript ID ENST00000606389.8) was detected. No unique peptides corresponding to other isoforms were identified. **(B)** Final, post-refinement, omit mFo-DFc map contoured at I/sigma = 3 for peptide complexed to PDZ-PDLIM5. **(C)** Details of hydrophobic interactions in each binding site (brown hydrophobic surface, cyan hydrophilic surface). PDLIM5-PDZ: Hydrophobic pocket formed by PDZ I70 and PDZ L18 accommodates the side chain of AAK1 L961.

A

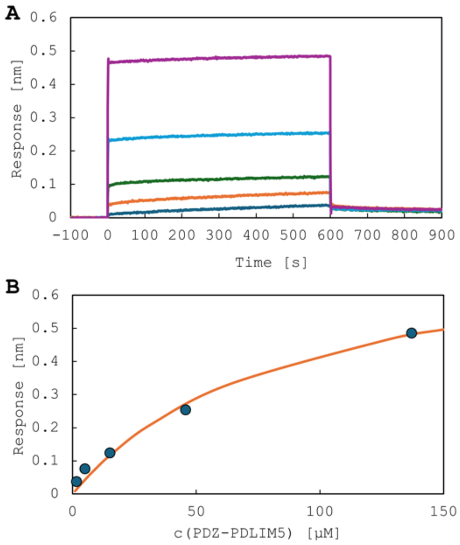

B

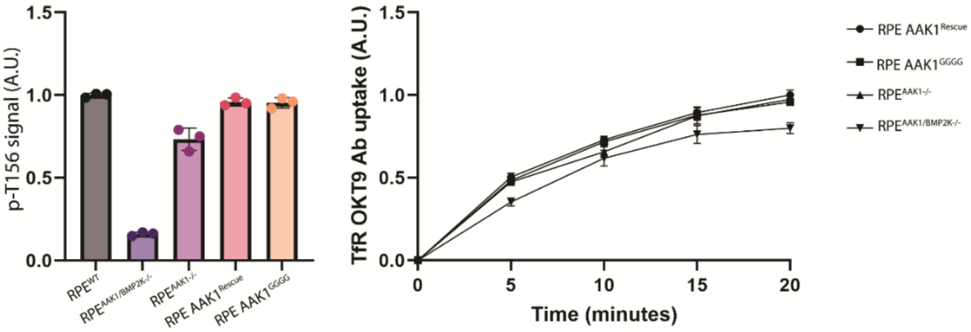

Supplementary Figure 10. – Related to Figure 7

(A) BLI analysis of AAK1 C-terminal peptide. A) BLI sensorgrams for 3-fold dilution row of PDLIM5-PDZ interaction with immobilized peptide AAK1 C-terminal peptide SSESSLPNLARSLLLVDQLIDL (blank subtracted), B) One-site binding model fitting of steady-state data of PDLIM5-PDZ interaction with AAK1 C-terminal peptide. The plots represent one typical example from four independent experimental repeats. **(B)** The AAK1 C-terminus is dispensable for AP2 μ phosphorylation and TfR uptake. Left panel: Quantification of AP2 μ T156 phosphorylation signal in different cell lines, including RPE^{WT}, RPE$^{AAK1/BMP2K-/-}$, RPE$^{AAK1-/-}$, RPE AAK1^{Rescue}, and RPE AAK1^{GGGG}. Bars represent the mean, n = three independent replicates. Right panel: Time-course analysis of TfR internalization measured over 20 min in RPE^{WT}, RPE$^{AAK1/BMP2K-/-}$, RPE$^{AAK1-/-}$, RPE AAK1^{Rescue}, and RPE AAK1^{GGGG}. Results demonstrate that the AAK1 C-terminus is not required for TfR uptake. Data are presented as mean ± SD, n = four independent biological replicates.

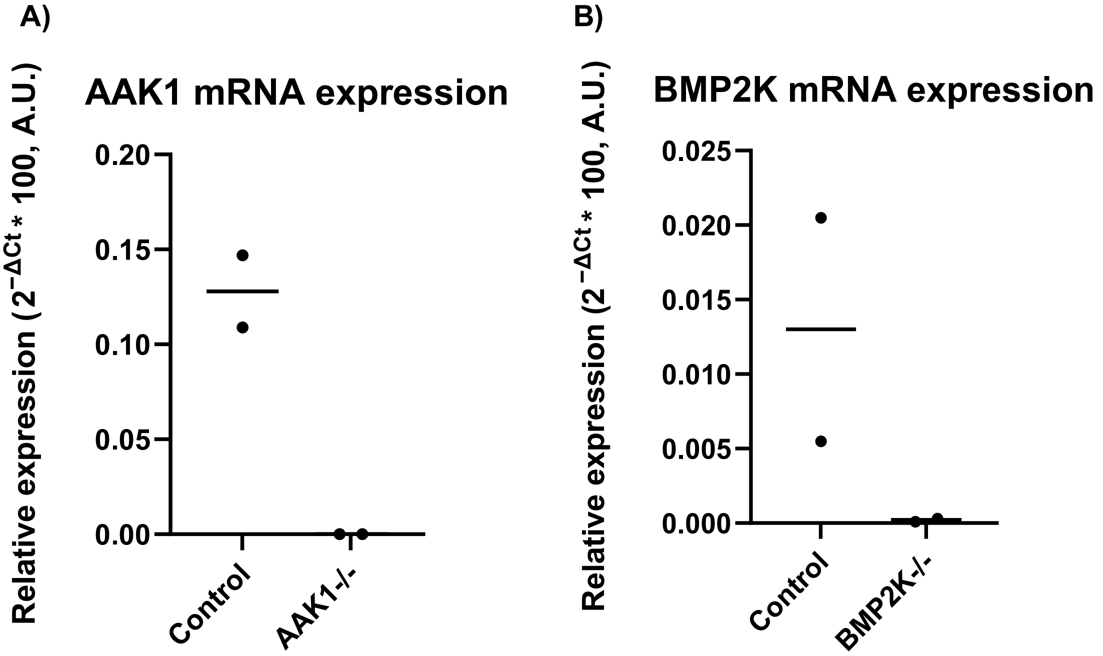

Supplementary Figure 11. Related to Materials and Methods.
AAK1 **(A)** and BMP2K **(B)** mRNA levels were assessed in the $RPE^{AAK1-/-}$ and $RPE^{BMP2K-/-}$ by RT–qPCR, normalized to GAPDH and compared to control. Data are presented as relative expression values, calculated as $2^{-\Delta Ct}$ *100. For $RPE^{AAK1-/-}$, a Ct value of 40 (corresponding to the maximum number of amplification cycles) was assigned, as signal was undetected in both replicates. Horizontal bars indicate mean values, n = two independent experiments.

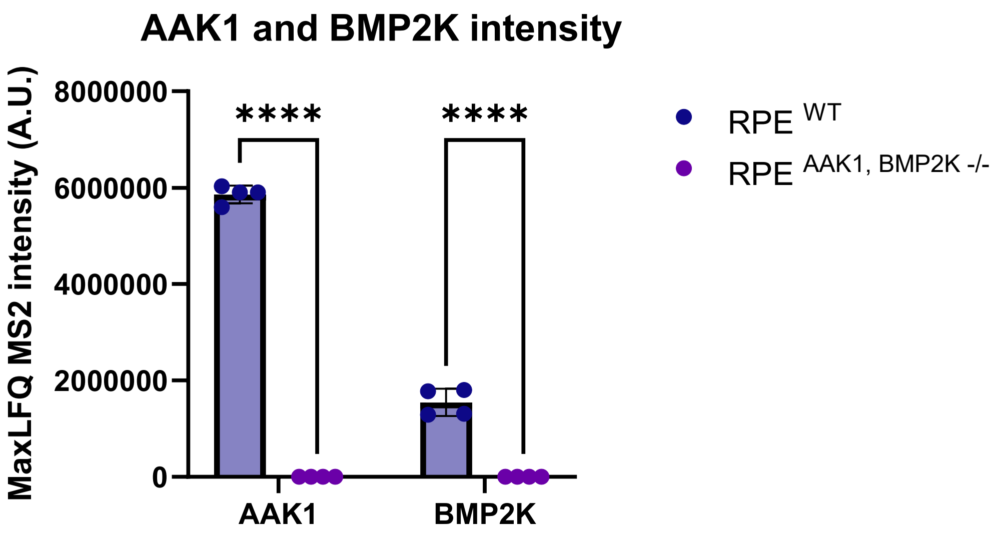

Supplementary Figure 12. Related to Materials and Methods. Loss of AAK1 and BMP2K protein expression in RPE$^{AAK1,\ BMP2K-/-}$.
DIA-MS analysis of AAK1 and BMP2K protein levels in RPE^{WT} and RPE$^{AAK1,\ BMP2K-/-}$ cells. MaxLFQ intensities are shown for individual biological replicates. Both AAK1 and BMP2K are undetectable in double-knockout cells, confirming successful disruption of the corresponding genes. Two-tailed unpaired t-test was used for statistical analysis, p-value < 0.0001. Data are presented as mean ± SD, n = four independent biological replicates.

		AAK1	BMP2K	
**Uniprot ID**	**Gene name**	**Phospho site analysis (Ion Score)**	**Phospho site analysis (Ion Score)**	**High-throughput ID in Phosphosite plus**
Q96HC4	**PDLIM5**	**98%**	**98%**	✓
O75112	**LDB3**	**100%**	**100%**	✓
Q86VX9	**MON1A**	**100%**	**100%**	✓
Q9Y490	**TALIN1**	**100%**	**100%**	✓
P41229	**KDM5C**	**100%**	**100%**	**X**
Q2KHR3	**QSER1**	**99%**	**99%**	**X**
Q96CW1	**AP2M1**	**100%**	**100%**	✓
Q9H9J2	**MRPL44**	**99%**	**99%**	**X**
Q68E01	**INTS3**	**99%**	**99%**	**X**
Q9NRD8	**DUOX2**	**99%**	**99%**	**X**
P26373	**RPL13**	**99%**	**99%**	✓
Q96QB1	**DLC1**	**61%**	**50%**	✓
Q6ZRS4	**ITPRID1**	**98%**	0%	✓
P49757	**NUMB**	0%	0%	**X**
Q9BXS5	**AP1M1**	0%	0%	✓
P98082	**DAB2**	0%	0%	**X**
Q5SW96	**ARH**	0%	0%	**X**
Q6IPM2	**IQCE**	0%	0%	**X**
Q12816	**TRO**	0%	0%	✓
Q9NZ56	**FMN2**	0%	0%	✓
Q68DA7	**FMN1**	0%	0%	**X**
P49407	**ARRB1**	0%	0%	✓
O60271	**SPAG9**	0%	0%	**X**
Q6PFW1	**PPIP5K1**	0%	0%	**X**
Q96CW1	**P-AP2M1**	0%	0%	✓
Q9UI08	**ENAH**	0%	0%	**X**
Q8TDZ2	**MICAL1**	0%	0%	**X**
Q15735	**INPP5J**	0%	0%	**X**
Q6NSJ2	**PHLDB3**	0%	0%	✓
Q92633	**LPAR1**	0%	0%	**X**

Supplementary Table 1. MS validation of threonine phosphorylation in peptide products after NADH-coupled kinase assay. Reaction products were analyzed by PRM to confirm phosphorylation at the specified threonine site. The table displays experimental ion scores for threonine phosphorylation, indicating confidence levels for detection. For DLC1 and ITPRID1, only trace levels of phosphopeptides were detected, falling below the sensitivity threshold of the NADH-coupled kinase assay. Data from PhosphoSitePlus is also referenced for comparison, corroborating our experimental detection and identification of phosphorylation sites.

Protein name	Sequence							
MICAL1	S	L	S	E	Q	G	T	G
PPIP5K1	G	L	G	S	Q	C	T	G
RUNDC3A	E	L	Q	E	Q	L	T	G
ENAH	V	L	N	S	Q	E	T	G
PHLDB3	S	I	G	L	Q	R	T	G
FMN1	G	L	E	H	Q	Q	T	G
CCDC88A	V	L	A	G	Q	W	T	G
INPP5J	R	L	G	T	Q	S	T	G
P-AP2μ	Q	I	T	pS	Q	V	T	G
SPAG9	E	T	R	N	V	S	T	G
DAB2	K	I	I	D	E	K	T	G
ARRB1	E	E	E	E	D	G	T	G
ITPRID1	R	A	V	A	L	G	T	G
DLC1	G	S	V	N	W	R	T	G
IQCE	S	P	I	A	Q	A	T	G
TRO	Q	G	S	Q	S	P	T	G
ARH	I	L	T	D	N	L	T	N
FMN2	D	S	Q	A	L	Q	T	G
NUMB	R	V	V	D	E	K	T	K
AP1μ	E	G	H	K	L	E	T	G
LPAR1	Q	R	S	E	N	P	T	G

Supplementary Table 2. Amino acid composition of peptides that failed to exhibit phosphoryl transfer in the NADH-coupled in vitro kinase assay and PRM-LC-MS validation.

Peptides were evaluated for their potential to undergo phosphorylation by AAK1 or BMP2K. Key sequence features associated with the absence of phosphoryl transfer are highlighted: negatively charged residues (yellow) inhibit phosphorylation; sequences lacking the [L/I]XXQXTG consensus motif (orange); and sequences with residues that deviate from optimal hydrophobicity or contain bulky tryptophan residues adjacent to the threonine in the consensus motif (purple). Residues that define the consensus motif or lack specific characteristics required for kinase activity are also indicated.

Uniprot ID	Gene name	Phosphosite position	Sequence	Tryptic peptide length	Solvent used	Identified in RPE cells by PRM
O75112	**LDB3**	282	ILAQMTG	20	0.2M acetic acid	X
P26373	**RPL13**	151	LATQLTG	13	0.2M acetic acid	X
P41229	**KDM5C**	1385	LLPQLTG	24	DMSO	✓
Q86VX9	**MON1A**	199	LSTQLTG	18	DMSO	X
Q96CW1	**AP2M1**	156	ITSQVTG	17	PBS	✓
Q96HC4	**PDLIM5**	290	ILAQITG	12	DMSO	✓
Q9Y490	**TALIN1**	2270	LKQQLTG	8	0.2M acetic acid	✓
Q9H9J2	**MRPL44**	224	LITQMTG	10	DMSO	✓
Q68E01	**INTS3**	287	LSPQFTG	23	0.2M acetic acid	✓
Q9BY66	**KDM5D**	1369	LLPQLTG	26	DMSO	✓
Q9NRD8	**DUOX2**	176	LANQVTG	27	DMSO	✓

Supplementary Table 3. Selection and validation of phosphopeptides for PRM-LC-MS analysis in RPE cells. Phosphopeptides were synthesized as standards to establish the PRM method, based on key selection criteria including tryptic peptide length, solubility, ionization efficiency, fragment ion intensity, and retention time reproducibility. The table details these selection criteria and annotates the phosphosites that were successfully detected in RPE cells using PRM with a ✓, while those not detected are marked with an X.

Uniprot number	Peptide sequence	Gene name	Log2 fold change	Adjusted p-value
P49407	EEEEDGTGSPQLNNR	ARRB1	-2.93	0.000
Q12816	AAQGSQSPTGHEGGTIQLK	TRO	-1.78	0.000
Q9NZ56	EDVLDSQALQTGELDSAHSLLTK	FMN2	-0.55	0.047
Q6IPM2	VPSPIAQATGSPVQEEAIVIIQSALR	IQCE	-0.97	0.009

Supplementary Table 4. Synthetic peptides with TG used in in vitro kinase experiments to test the selectivity of AAK1/BMP2K kinase domain.

Phosphopeptides containing threonine followed by glycine (TG motif) were selected for testing in-vitro in coupled kinase assay based on significant decreases in abundance observed in $RPE^{AAK1,BMP2K-/-}$ cells from global phosphoproteome profiling. Only phosphopeptides from proteins with stable overall abundance were included to ensure specificity. The table lists the corresponding Uniprot ID, tested peptide sequence, gene name, log2 fold change in abundance, and the adjusted p-value. Statistical analysis was performed using LIMMA with Benjamini-Hochberg correction. These peptides were synthesized in their non-phosphorylated forms and tested in NADH-coupled in vitro kinase assays to assess phosphorylation by AAK1 or BMP2K; no phosphorylation was observed.

Crystal	PDZ + AP2-associated protein kinase 1 peptide
PDB accession code	9F6S
	**Data collection and processing**
Space group	C 2 2 21
Cell dimensions - a, b, c (Å)	36.93, 37.84, 52.39
Cell dimensions - α, β, γ (°)	90, 90, 90
Resolution range (Å)	30.68 - 1.0 (1.036 – 1.0)
No. of unique reflections	39979 (3611)
Completeness (%)	98.88 (89.94)
Multiplicity	12.0 (7.6)
Mean I/σ(I)	24.56 (0.75)
$CC_{1/2}$	1 (0.466)
CC*	1 (0.797)
	**Structure solution and refinement**
R-work (%)	20.00 (38.06)
R-free (%)	21.35 (39.99)
R.m.s.d. - bonds (Å) / angles (°)	0.008 / 1.07
Average B factors ($Å^2$)	17.58
protein	16.26
solvent	26.51
Clashscore	2.82
Ramachandran favored/outliers (%)	98.84 / 0

Supplementary Table 5. Statistics of crystallographic data collection and refinement for PDLIM5-PDZ and VDQLIDL peptide. Numbers in parentheses refer to the highest resolution shell.

Antibody/Reagent table			
**Target/Description**	**Manufacturer**	**Catalogue no.**	**Application**
AAK1 (aa 564-707)	Atlas antibodies	HPA020289	WB
AAK1 (KKFFDSRREQGGSGLGSGSSGGGGSTSGL)	Kadlecova Lab/Eurogentec	49	IP
AAK1 Kinase domain (aa 1-365)	Abcam	[EPR5126(2)] (ab134971)	WB
BMP2K (E-8)	Santa Cruz Bio	sc-514681	WB
AP2 alpha-Adaptin	ThermoFisher Scientific	AC1-M11	WB
AP2 mu subunit	BD transduction laboratories	611351	WB
p-AP2M1 (T156) (D4F3)	Cell Signaling	73995	WB, In-cell ELISA
PDLIM5/ENH	antibodies.com	A14933	IP
PDLIMS (G-2)	Santa Cruz Bio	sc-515621	WB
PDLIM5/ENH	Abcam	A8196559	WB
Paxillin	BD transduction laboratories	610052	WB
Integrin alpha 2	Abcam	[EPR5788] (ab133557)	WB
Clathrin Light chain	Santa Cruz Bio	sc-12735	WB
Integrin alpha 2 CD-49b	Novus biologicals	N8100-2608	Internalization assay
Transferrin CD71 (OKT-9)	InVivoMAb	BE0023	Internalization assay
Human Integrin alpha 2/CD49b Alexa Fluor* 647-conjugated Antibody	Biotechne	FAB1233R	Microscopy
AF 680-conjugated donkey anti rabbit IgG	Jackson Immuno Research	711-625-152	WB (Li-Cor)
AF 790-conjugated donkey anti mouse IgG	Jackson Immuno Research	715-655-150	WB (Li-Cor)
Anti Goat igG HRP conjugate	Promega	V805A	WB
Anti mouse igG HRP conjugate	Promega	W4028	WB
Anti Rabbit igG HRP conjugate	Promega	W4018	WB
JF 503-HaloTag Ligand	Lavis Lab Janelia		Microscopy
JF 552-HaloTag Ligand	Lavis Lab Janelia		Microscopy
JF503-cpSNAP-Tag ligand	Lavis Lab Janelia		Microscopy
JF552-cpSNAP-Tag ligand	Lavis Lab Janelia		Microscopy
JF650-HaloTag Ligand	Lavis Lab Janelia		Microscopy
Phalloidin 647	Invitrogen	A2287	Microscopy
WGA (CF 405)	Biotium	29028-1	Microscopy
GAPDH (G-9)	Santa Cruz Bio	sc-365062	WB
HSP90	Cell Signaling	4874	WB

Supplementary Table 7. Antibodies and microscopy reagents used in this study.
