# Supplementary material for: AAK1-mediated phosphorylation of PDLIM5 and Talin1 promotes focal adhesion disassembly to accelerate cell migration
Source: Nat Commun. 2026 May 4;17:6023. doi: 10.1038/s41467-026-72501-w (PMC13346677; doi:10.1038/s41467-026-72501-w)
Supplement: Supplementary file 2 — Description of Additional Supplementary Information [file 41467_2026_72501_MOESM2_ESM.pdf]

Description of Additional Supplementary Files

File Name: Supplementary Data 1
Description: (In silico screen, related to figure 1) CSV file listing candidate AAK1/BMP2K substrates identified by the in silico screen, comprising proteins that contain a [L/I]XXQXTG motif within predicted intrinsically disordered regions or surface-exposed protein domains.

File Name: Supplementary Data 2
Description: (Statistical analyses of MS data) Statistical analyses for all MS experiments presented in this study.

File Name: Supplementary Data 3
Description: (GO analyses) GO analyses for this study, as exported from the DAVID GO platform.

File Name: Supplementary Data 4
Description: (Transition lists) MS settings for all the experiments presented in this manuscript.

File Name: Supplementary Movie 1
Description: (Related to Figure 5) RPE cells stably expressing mNeonGreen-paxillin were seeded either on PLL or collagen (as specified in the Methods section) and imaged with live-cell TIRFM for 40 minutes. This movie highlights the absence of FA formation and cell spreading in RPE cells seeded on PLL.

File Name: Supplementary Movie 2
Description: (Related to Figure 6abc) RPE cells co-expressing mNeonGreen-paxillin, PDLIM5-HaloTag, and mScarlet-AAK1 were seeded on collagen and immediately imaged via live-cell TIRFM for 5 hours. This movie captures the temporal hierarchy of PDLIM5 and AAK1 recruitment during cell spreading and FA formation.

File Name: Supplementary Movie 3
Description: (Related to Figure 6abc) A magnified view of RPE cells co-expressing mNeonGreen-paxillin, PDLIM5-HaloTag, and mScarlet-AAK1 seeded on collagen, imaged via live-cell TIRFM for 5 hours. This close-up highlights the sequential recruitment of AAK1 and PDLIM5 to forming and disassembling FAs, showcasing their dynamic roles in FA maturation and disassembly.
