# Supplementary material for: AAK1-mediated phosphorylation of PDLIM5 and Talin1 promotes focal adhesion disassembly to accelerate cell migration
Source: Nat Commun. 2026 May 4;17:6023. doi: 10.1038/s41467-026-72501-w (PMC13346677; doi:10.1038/s41467-026-72501-w)
Supplement: Supplementary file 10 — Reporting Summary [file 41467_2026_72501_MOESM10_ESM.pdf]

nature portfolio

Corresponding author(s): Zuzana Kadlecova

Last updated by author(s): Mar 24, 2026

Reporting Summary

Nature Portfolio wishes to improve the reproducibility of the work that we publish. This form provides structure for consistency and transparency in reporting. For further information on Nature Portfolio policies, see our Editorial Policies and the Editorial Policy Checklist.

Statistics

For all statistical analyses, confirm that the following items are present in the figure legend, table legend, main text, or Methods section.

n/a	Confirmed	
☐	☒	The exact sample size (n) for each experimental group/condition, given as a discrete number and unit of measurement
☐	☒	A statement on whether measurements were taken from distinct samples or whether the same sample was measured repeatedly
☐	☒	The statistical test(s) used AND whether they are one- or two-sided<br>*Only common tests should be described solely by name; describe more complex techniques in the Methods section.*
☐	☐	A description of all covariates tested
☐	☒	A description of any assumptions or corrections, such as tests of normality and adjustment for multiple comparisons
☐	☒	A full description of the statistical parameters including central tendency (e.g. means) or other basic estimates (e.g. regression coefficient) AND variation (e.g. standard deviation) or associated estimates of uncertainty (e.g. confidence intervals)
☐	☒	For null hypothesis testing, the test statistic (e.g. F, t, r) with confidence intervals, effect sizes, degrees of freedom and P value noted<br>*Give P values as exact values whenever suitable.*
☒	☐	For Bayesian analysis, information on the choice of priors and Markov chain Monte Carlo settings
☒	☐	For hierarchical and complex designs, identification of the appropriate level for tests and full reporting of outcomes
☐	☒	Estimates of effect sizes (e.g. Cohen's d, Pearson's r), indicating how they were calculated

Our web collection on statistics for biologists contains articles on many of the points above.

Software and code

Policy information about availability of computer code

Data collection	A detailed description of data collection procedures is provided in the Methods section of the manuscript. All the used instruments are commercially available and were controlled using the software provided by the manufacturer.
Data analysis	A detailed description of data analysis is provided in the Methods section of the manuscript

For manuscripts utilizing custom algorithms or software that are central to the research but not yet described in published literature, software must be made available to editors and reviewers. We strongly encourage code deposition in a community repository (e.g. GitHub). See the Nature Portfolio guidelines for submitting code & software for further information.

Data

Policy information about availability of data

All manuscripts must include a data availability statement. This statement should provide the following information, where applicable:

- Accession codes, unique identifiers, or web links for publicly available datasets
- A description of any restrictions on data availability
- For clinical datasets or third party data, please ensure that the statement adheres to our policy

All accession codes/unique identifiers and repository links for the deposited datasets are listed in the manuscript's Data Availability statement. Any further inquiries can be directed to the corresponding author.

Research involving human participants, their data, or biological material

Policy information about studies with human participants or human data. See also policy information about sex, gender (identity/presentation), and sexual orientation and race, ethnicity and racism.

Reporting on sex and gender	N.A.
Reporting on race, ethnicity, or other socially relevant groupings	N.A.
Population characteristics	N.A.
Recruitment	N.A.
Ethics oversight	N.A.

Note that full information on the approval of the study protocol must also be provided in the manuscript.

Field-specific reporting

Please select the one below that is the best fit for your research. If you are not sure, read the appropriate sections before making your selection.

[x] Life sciences [ ] Behavioural & social sciences [ ] Ecological, evolutionary & environmental sciences

For a reference copy of the document with all sections, see nature.com/documents/nr-reporting-summary-flat.pdf

Life sciences study design

All studies must disclose on these points even when the disclosure is negative.

Sample size	Experiments were repeated in independent biological replicates and with sample sizes used as described in the Methods and figure legends.
Data exclusions	For TIRFM quantitative and colocalization analyses, only in-focus cells were included (manual masking of the cell footprint prior to automated quantification). For perturbation experiments relying on siRNA, knockdown efficiency was confirmed by western blotting in parallel with each experiment; experiments were excluded from downstream analysis when depletion was clearly inefficient. For experiments requiring transient mEmerald–Talin1 expression for long-term live TIRFM, Talin1 transfection efficiency was relatively low; therefore, analyses were restricted to cells expressing sufficient Talin1 fluorescence for reliable tracking throughout the imaging period, and cells with insufficient expression were not analyzed.
Replication	Experiments were repeated in independent biological replicates as described in the Methods and figure legends.
Randomization	For quantitative TIRFM experiments, immunofluorescence, Incucyte and FRAP analysis, randomization was ensured by taking representative pictures of different fields (or individual cells) from the same sample at different locations within the sample. All downstream image quantification and analysis (including segmentation, tracking, and intensity/colocalization measurements) was performed using a pre-defined automated image-analysis pipeline to exclude operator bias, with the only manual step being masking of in-focus cell footprints prior to automated analysis. Randomization for other experiments was not formally applied, as experimental groups were defined by genotype and/or treatment.
Blinding	Blinding was not performed for the in vitro experiments because sample identity had to be known during experimental setup and handling (e.g., distinguishing control versus transfected cells, drug-treated versus untreated conditions, and recombinant-protein interaction conditions). All experimental groups were processed in parallel and subjected to the same procedures according to the relevant protocols

Reporting for specific materials, systems and methods

We require information from authors about some types of materials, experimental systems and methods used in many studies. Here, indicate whether each material, system or method listed is relevant to your study. If you are not sure if a list item applies to your research, read the appropriate section before selecting a response.

Materials & experimental systems

n/a	Involved in the study
☐	☒ Antibodies
☐	☒ Eukaryotic cell lines
☒	☐ Palaeontology and archaeology
☒	☐ Animals and other organisms
☒	☐ Clinical data
☒	☐ Dual use research of concern
☒	☐ Plants

Methods

n/a	Involved in the study
☒	☐ ChIP-seq
☒	☐ Flow cytometry
☒	☐ MRI-based neuroimaging

Antibodies

Antibodies used	All antibodies used in this study are commercially available, listed with supplier, catalogue number, and clone name in Supplementary Table S7, and have been validated by their respective manufacturers for the indicated applications.
Validation	Custom anti-AAK1 N-terminus rabbit polyclonal with known epitope located in N-terminus of AAK1 with sequence KKFFDSRREQGGSGLGSGSSGGGGSTSGL was used and validated for immunoprecipitation using CRISPR-generated RPE AAK1 knock-out cells as a negative control and we confirmed absence of known AAK1 interactors in results obtained from knock-out cell line. Other antibodies used in this study are all commercially available and have been validated in other studies and were not separately validated here

Eukaryotic cell lines

Policy information about cell lines and Sex and Gender in Research

Cell line source(s)	ATCC
Authentication	Cell lines were obtained from ATCC and were supplier-authenticated.
Mycoplasma contamination	All cell lines were tested and confirmed negative for mycoplasma on a regular basis with MycoAlert® Mycoplasma Detection Kit (Lonza)
Commonly misidentified lines (See ICLAC register)	No commonly misidentified lines were used in this study

Plants

Seed stocks	*Report on the source of all seed stocks or other plant material used. If applicable, state the seed stock centre and catalogue number. If plant specimens were collected from the field, describe the collection location, date and sampling procedures.*
Novel plant genotypes	*Describe the methods by which all novel plant genotypes were produced. This includes those generated by transgenic approaches, gene editing, chemical/radiation-based mutagenesis and hybridization. For transgenic lines, describe the transformation method, the number of independent lines analyzed and the generation upon which experiments were performed. For gene-edited lines, describe the editor used, the endogenous sequence targeted for editing, the targeting guide RNA sequence (if applicable) and how the editor was applied.*
Authentication	*Describe any authentication procedures for each seed stock used or novel genotype generated. Describe any experiments used to assess the effect of a mutation and, where applicable, how potential secondary effects (e.g. second site T-DNA insertions, mosiacism, off-target gene editing) were examined.*
