# Supplementary material for: AAK1-mediated phosphorylation of PDLIM5 and Talin1 promotes focal adhesion disassembly to accelerate cell migration
Source: Nat Commun. 2026 May 4;17:6023. doi: 10.1038/s41467-026-72501-w (PMC13346677; doi:10.1038/s41467-026-72501-w)
Supplement: Supplementary file 11 — Transparent Peer Review file [file 41467_2026_72501_MOESM11_ESM.pdf]

nature portfolio

Peer Review File

AAK1-mediated phosphorylation of PDLIM5 and Talin1 promotes focal adhesion disassembly to accelerate cell migration

Corresponding Author: Dr Zuzana Kadlecova

This file contains all reviewer reports in order by version, followed by all author rebuttals in order by version.

Version 0:

Reviewer comments:

Reviewer #1

(Remarks to the Author)
The authors have investigated focal adhesion proteins PDLIM5 and Talin1. They have performed a large set of different sophisticated experiments in an excellent manner.
The one criticism I have is that they have analysed FRAP data on WT and mutant protein by fitting mathematical curves and derive rate constants, and then state: “mutant recovered fluorescence twice as faster than the wild type (Fig 4C), even though its mobile and immobile fractions at FAs were unchanged (Fig S5A), likely indicating a faster off-rate and a shorter residence time within the adhesion”. Indeed, a faster off-rate leads to a shorter residence time (=1/off-rate), and if then “mobile and immobile fractions were unchanged”, this also points to a faster on-rate (%immobile / %mobile = on-rate/off-rate). It would be nice if the authors would comment on this and hypothesise how this fits in the mechanism of action. In addition, it is a pity that the authors do not discuss nor cite Legerstee et al., Sci 2019 (Dynamics and distribution of paxillin, vinculin, zyxin and VASP depend on focal adhesion location and orientation), because it is very relevant for interpretation of their current results.

Reviewer #2

(Remarks to the Author)
Review report on Nature Communications manuscript NCOMMS-25-57154, by Krocianova et al.

AAK1-mediated phosphorylation of PDLIM5 and Talin1 promotes focal adhesion disassembly to accelerate cell migration

In this manuscript, Krocianova et al. identify PDLIM5 and Talin1 as direct AAK1 substrates. They also show that AAK1 and PDLIM5 localize to mature focal adhesions (FAs), affect FA turnover rate, and have an impact on cell migration. Therefore, AAK1 exhibits a function distinct from that of the related BMP2K. The topic of this study is of great importance, as the disassembly of FAs has a direct impact on cell migration.
The data in this manuscript are of high quality and obtained with appropriate and varying techniques. The engineered cell lines provide powerful tools to address the questions in this study. However, some of the conclusions appear overstated. Furthermore, the title and the abstract state that AAK1-mediated phosphorylation of PDLIM5 and Talin 1 promotes FA disassembly to accelerate cell migration, but the actual manuscript contains very little data on Talin 1.

Major comments:

- The statements that PDLIM5 and Talin 1 were identified as direct AAK1 substrates within FAs and that AAK1-dependent phosphorylation of PDLIM5 regulates localization, interactions, and FA disassembly (abstract, page 13 and 15, and Figure 7F) are not justified. Data showing that AAK1 phosphorylates PDLIM5 and Talin 1 within FAs and how this phosphorylation event affects FA disassembly is missing. The authors should include mutated versions of PDLIM5 and Talin 1, that can not be phosphorylated by AAK1, in the experimental setup presented in Figure 6 to support the conclusions drawn. Alternatively, phosphospecific antibodies against PDLIM5 and Talin 1 could be used in immunofluorescent staining and

proximity ligation assays in WT and AAK1-/- cells.

- Talin 1 localization in the cells is never shown in this manuscript. Imaging showing Talin 1, AAK1, and PDLIM5 co-localization at FAs would be needed.

- Talin 1 should be added to the discussion with proper references. The identification of a phosphosite (T2270) in the Talin 1 R12 domain, and its possible functional consequences, as well as the contribution of other Talin 1 phosphorylating kinases, should be discussed. Here are some useful references: https://doi.org/10.1038/ncomms11966, https://doi.org/10.1016/j.cell.2019.08.034, https://doi.org/10.1073/pnas.2314947121, https://doi.org/10.1016/j.jbc.2021.100837).

- On page 6, lines 156-159, it is reported that PDLIM5 and Talin 1, in addition to AP2 µ T156, are the principal substrates of AAK1. The other identified phosphorylated peptides are, however, not shown, making it difficult to judge this result.

- It is reported on page 6 that upon genetic or pharmacological perturbation, PDLIM5 and Talin 1 levels remained constant (Figure S3), but in Figure 2C, Talin 1 is upregulated. Adding statistical analysis in Figure S3 might show that Talin 1 is upregulated in these samples as well. However, if the data is not coherent, it should still be commented.

- The overlap of the data in genetic and pharmacological perturbations, or the lack of it, should be reported when presenting the results of Figure 2. Specifically, the presence of PDLIM5 and Talin 1 only in one of the panels should be discussed.

- The specificity of the inhibitors should be explained. E.g., LP-935509 is used as an AAK1 and BMP2K inhibitor (Figure 2) or an AAK1 inhibitor (Figure 3). In the referred study, it is described as a NAK family inhibitor.

- The cell lines SH-SY5Y and MDA-MB-231 should be introduced and references added. In this study, conclusions are drawn from these cell lines that AAK1-dependent kinase-driven motility is not limited to epithelial cells (page 8, line 223-226). However, MDA-MB-231 has an epithelial origin, and ATCC describes the morphology as epithelial, even though these cells have mesenchymal characteristics. Overall, the identity of these cell lines should be clarified better.

- On page 12, rows 314-315, it is nicely described that the cells were cultivated on collagen to recover FA components. How were the cells cultured in other assays, since they appear to form FAs? This information should be included.

- PDLIM5 and Talin 1 were not found as AAK1 interactors in Figure 5G. They are, however, present in the MS result lists with lower intensity ratios. This should be commented on, and possible explanations discussed. In addition, it should be discussed that in co-IP experiments, the cells are lysed, which might lead to the interaction of proteins normally separated in different compartments.

- The model in Figure 7F involves Talin 1, even though the data supporting this is insufficient.
Talin 1 should also be depicted as a dimer in a stretched form in mature FAs.

- Figure S1 has several inaccuracies that should be clarified: the number of lanes in Ponceau control does not match the immunoblot, unclear which Ponceau panel is corresponding to which immunoblot in C and D, the b tubulin lane is broken in E, and the figure legend appears to be swapped between E and F, and in G there is only one panel (not right and left). Information is missing about the GAPDH and b-tubulin (Table S7), the Ponceau S staining, how the membranes were stained, and what was used to capture the images, and information on how/if the data was quantified and/or normalized to loading controls, and the number of replicas. The panel H should also include the WT sample.

- The accuracy of data presentation in the text and figures should be improved (listed in the minor comments).

Minor comments:

Text:

- Abbreviations such as PM and CCP are not consistently used or spelled out when introduced.
- On page 3, row 100, BMP2K exhibits prominent concentration within CCPs at the PM, rather than complete concentration.
- On page 6, rows 156-159, the sentence is complex.
- On page 6, row 163. Based on the results presented in this section, the sentence “The similar sensitivity of both PDLIM5 and Talin1 suggests that AAK1 or BMP2K may regulate mechanosensitive adhesion module, extending their signalling beyond endocytosis.” is too far-reaching.
- On page 7, row 175, a reference to the dataset is missing, or where can this dataset be found?
- On page 8, row 214-216, the sentence (In contrast, the identification of PDLIM5 and Talin1 as novel substrates, together with the broad distribution of AAK1 across the plasma membrane, strongly suggests a previously unrecognized role for AAK1 in FA maintenance during cell migration) is illogical and appears abruptly at this point in the manuscript.
- On page 9, number 3 is missing from the reference Fig. 3F, G.
- The data on page 11, rows 283-290, should be better presented and explained. This also applies to the corresponding figure legend and to the materials and methods. Now it is unclear if this is a co-IP of PDLIM5 in WT and AAK1-/- cells.
- On page 11, a reference to Fig. 4D is missing.
- On page 12, row 314, a reference is missing for the data “Co-immunoprecipitation of endogenous AAK1 from collagen-adherent RPE cells under steady-state conditions recovered multiple FA components, with α-actinin-4 being the most

enriched".
- The AAK1 splice variants are presented first on page 17, row 404. The splice variant information should be included in Figure 1A.
- The data in Figure 7C should be better explained on page 17, rows 410-412. It should be mentioned that this is a co-IP experiment in WT and AAK1-/- cells.
- On page 17, row 429, a reference to Figure 7E is missing.
- On page 30, rows 819-20, the authors state that genetic modifications were verified by RT-PCR and WB, but no RT-PCR data are shown in the manuscript.
- On page 31, row 870, this data is missing: "the population with the AAK1 expression closest to that of RPEWT cell lines was selected for further experiments."
- On page 31, rows 870-871, the authors state that a population of mScarlet-AAK1-WT and mScarlet-AAK1-GGGG cell lines were selected based on having expression closest to RPE-WT levels. However, no comparison of the expression to RPE-WT cells is included in Figure S1H.
- The Materials and methods section includes many redundant titles (e.g. MS and data analysis), which hamper the clarity of this section.

Figures:

- Figure 1A: The source of the figure or data is missing, and the abbreviations (such as SLiMS) and colors are not explained.
- Figure 1B and C: The zoomed areas are not marked, and a scale bar is missing from the upper panel. Why are the morphologies of the mScarlet-BMP2K and mScarlet-AAK1 cells very different?
- Figure 1H: Nomenclature for the positive control is not coherent with the main text (AP2 µ-subunit peptide containing residue T156 (ITSQVTG) vs. AP2M1). Why is the data normalized to PDLIM5 in H and I and not to the positive control? This should be explained. Why is there an arrow between panels I and J?
- Figure 2: Why are different Log2 fold changes used for different panels? This should be explained.
- Figure 3B: Statistical comparison between RPE WT and RPE BMP2K rescue is missing.
- Figure 3C: Lacks a unit on the y-axis.
- Figure 3D: Statistical comparison to the positive control is missing.
- Figure 3E: Contains data for RPE, but RPE is not mentioned in the figure legend. The title for the y-axis is missing.
- Figure 3F: It is unclear how FA lifetimes were assessed. With paxillin or something else? It should be explained in the figure legend.
- Figure 3H (left plot): lacks a unit on the y-axis. As a supporting material for Figure 3H, Figure S1F should contain RPE AAK-/- sample as well.
- Figure 4A: The schematic illustration (upper panel) is not explained in the figure legend.
- Figure 4B: It is unclear how FA lifetimes were assessed. With paxillin or something else? It should be explained in the figure legend.
- Figure 4C: An image showing PDLIM5T290D-HaloTag distribution should be included in the upper panel.
- Figure 4D: Scale bars and y-axis values and units are missing. Should it be written Paxillin instead of Phalloidin in the left panel? The images are unclear, and it is hard to tell what these structures are. The colors in the images are not explained.
- Figure 4E, a title is missing.
- Figure 5: Why is log2 fold change > 1 used here, when in previous analyses > 0.3 was used?
- Figure 6A: scale bar is missing.
- Figure 6B: Information about the time is missing.
- Figure 7A: Isoform misspelled on the first row. Is the LIDL sequence specific for only these isoforms?
- Figure 7 D: The figure legend contains (D) twice.
- Figure 7 E: Missing relevant statistical comparisons, especially RPE-WT to RPE AAK1-rescue, to evaluate the effect of RPE AAK1-GGGG. The authors may want to refer to this figure on page 17, line 29, instead of referring only to S1H.
- Figure 7 F: Is Talin1 phosphorylation at this site known for FA disassembly? The presented data does not support that. It should be added which parts are hypothetical.
- Figure S6E: Lacks statistical analysis.

Reviewer #3

(Remarks to the Author)
I co-reviewed this manuscript with one of the reviewers who provided the listed reports. This is part of the Nature Communications initiative to facilitate training in peer review and to provide appropriate recognition for Early Career Researchers who co-review manuscripts.

Version 1:

Reviewer comments:

Reviewer #1

(Remarks to the Author)
The authors did adequately respond to my criticisms. The paper is in my view now ready for publications. I apologise for not reponding quicker

(Remarks on code availability)

.

Reviewer #2

(Remarks to the Author)
Review report on Nature Communications manuscript NCOMMS-25-57154A by Krocianova et al.

AAK1-mediated phosphorylation of PDLIM5 and Talin1 promotes focal adhesion disassembly to accelerate cell migration

I have reviewed the revised manuscript by Krocianova et al. and find that the authors have addressed the concerns raised in the previous review very thoroughly. The revisions have substantially improved the manuscript and more convincingly support the conclusions drawn. I recommend one minor improvement: please add x and y axis titles to Figures S4A–D, S8E, and S11A–B to ensure full clarity. Addressing these final details will, in my opinion, make the manuscript suitable for publication.

(Remarks on code availability)

Reviewer #3

(Remarks to the Author)
I co-reviewed this manuscript with one of the reviewers who provided the listed reports. This is part of the Nature Communications initiative to facilitate training in peer review and to provide appropriate recognition for Early Career Researchers who co-review manuscripts.

(Remarks on code availability)

Open Access This Peer Review File is licensed under a Creative Commons Attribution 4.0 International License, which permits use, sharing, adaptation, distribution and reproduction in any medium or format, as long as you give appropriate credit to the original author(s) and the source, provide a link to the Creative Commons license, and indicate if changes were made.
In cases where reviewers are anonymous, credit should be given to 'Anonymous Referee' and the source.
The images or other third party material in this Peer Review File are included in the article's Creative Commons license, unless indicated otherwise in a credit line to the material. If material is not included in the article's Creative Commons license and your intended use is not permitted by statutory regulation or exceeds the permitted use, you will need to obtain permission directly from the copyright holder.
To view a copy of this license, visit https://creativecommons.org/licenses/by/4.0/

Dear Reviewers,

Thank you for the thoughtful and constructive evaluation of our manuscript. We are particularly grateful that the Reviewers highlighted the importance of the question addressed and noted the high quality of the data and breadth of complementary approaches. In response to the main themes raised (strengthening the experimental support for Talin1 phosphorylation, avoiding overstatement and clarifying experimental context) we have revised the manuscript extensively, expanded the Methods section, and provided Source Data files. We believe these revisions address the Reviewers' concerns and strengthen the manuscript, and we hope the revised version meets the editorial expectations for Nature Communications. We provide a detailed point-by-point response below.

REVIEWER COMMENTS

Reviewer #1 (Remarks to the Author):

Remark: The authors have investigated focal adhesion proteins PDLIM5 and Talin1. They have performed a large set of different sophisticated experiments in an excellent manner.
The one criticism I have is that they have analysed FRAP data on WT and mutant protein by fitting mathematical curves and derive rate constants, and then state: "mutant recovered fluorescence twice as faster than the wild type (Fig. 4C), even though its mobile and immobile fractions at FAs were unchanged (Fig. S5A), likely indicating a faster off-rate and a shorter residence time within the adhesion". Indeed, a faster off-rate leads to a shorter residence time (=1/off-rate), and if then "mobile and immobile fractions were unchanged", this also points to a faster on-rate (%immobile / %mobile = on-rate/off-rate). It would be nice if the authors would comment on this and hypothesise how this fits in the mechanism of action. In addition, it is a pity that the authors do not discuss nor cite Legerstee et al., Sci 2019 (Dynamics and distribution of paxillin, vinculin, zyxin and VASP depend on focal adhesion location and orientation), because it is very relevant for interpretation of their current results.

Response: We thank the Reviewer for the overall positive assessment and for the insightful correction to our interpretation of data in Fig. 4C. We agree that faster FRAP recovery of the PDLIM5^{T290D} phosphomimetic mutant, despite an unchanged recovery plateau

(mobile/immobile fraction; Fig. S7A), is most consistent with accelerated exchange kinetics: an increased effective k_{off} (shorter residence time) accompanied by a commensurate increase in effective k_{on}, such that the steady-state partitioning between recoverable and non-recoverable pools remains similar. We have corrected this interpretation in the revised manuscript. We also appreciate the suggestion to cite Legerstee et al., which closely parallels our FRAP-based analysis of focal adhesion proteins. Legerstee et al. demonstrate that FA FRAP recoveries comprise multiple kinetic pools and that paxillin show comparatively slow recovery with a substantial stably associated fraction, whereas more actin-linked FA components such as zyxin and VASP exchange more rapidly. Importantly, they emphasize that faster recovery can reflect changes in effective binding kinetics (particularly k_{on} of the exchanging pool). We have now included this citation in the Results sections for Fig. 3H and Fig. 4C.

Reviewer #2 (Remarks to the Author):

Remarks: In this manuscript, Krocianova et al. identify PDLIM5 and Talin1 as direct AAK1 substrates. They also show that AAK1 and PDLIM5 localize to mature focal adhesions (FAs), affect FA turnover rate, and have an impact on cell migration. Therefore, AAK1 exhibits a function distinct from that of the related BMP2K. The topic of this study is of great importance, as the disassembly of FAs has a direct impact on cell migration.
The data in this manuscript are of high quality and obtained with appropriate and varying techniques. The engineered cell lines provide powerful tools to address the questions in this study. However, some of the conclusions appear overstated. Furthermore, the title and the abstract state that AAK1-mediated phosphorylation of PDLIM5 and Talin-1 promotes FA disassembly to accelerate cell migration, but the actual manuscript contains very little data on Talin-1.

Response: We thank the Reviewer for the positive assessment of the significance of this work for understanding FA disassembly and cell migration, and for recognizing the high quality of the data and the broad utility of the engineered cell lines. We appreciate the concern that parts of the original text could be interpreted as somewhat overstated. Accordingly, we extensively revised the Abstract and relevant sections of Results and Discussion text to more clearly distinguish direct observations from working hypotheses. To

address the concern that the functional role of Talin1 T2270 phosphorylation was not addressed, we expanded Fig. 6 and added lifetime-aligned FA analysis of $Talin1^{WT}$ and $Talin1^{T2270A}$ (along with $PDLIM5^{WT}$ and $PDLIM^{T290A}$) and by including Talin1 in co-localization experiments with AAK1 in Fig. 5. Finally, we expanded the Discussion to outline a plausible molecular consequence of Talin1 phosphorylation and to explain how convergent, orthogonal evidence supports our model connecting AAK1 recruitment and AAK1-responsive phosphosites with focal-adhesion turnover and cell migration.

Major comments:

Major comment 1: The statements that PDLIM5 and Talin-1 were identified as direct AAK1 substrates within FAs and that AAK1-dependent phosphorylation of PDLIM5 regulates localization, interactions, and FA disassembly (abstract, page 13 and 15, and Figure 7F) are not justified. Data showing that AAK1 phosphorylates PDLIM5 and Talin-1 within FAs and how this phosphorylation event affects FA disassembly is missing. The authors should include mutated versions of PDLIM5 and Talin-1, that cannot be phosphorylated by AAK1, in the experimental setup presented in Figure 6 to support the conclusions drawn. Alternatively, phosphospecific antibodies against PDLIM5 and Talin-1 could be used in immunofluorescent staining and proximity ligation assays in WT and AAK1-/- cells.

Response: We thank the Reviewer for this constructive criticism and advice. We removed statements implying that AAK1-dependent phosphorylation was demonstrated to occur in situ within focal adhesions. Our study does not include live-cell compatible phospho-specific reporters capable of directly visualizing endogenous site-specific phosphorylation events. Our conclusions are based on perturbation strategies, targeted phosphoproteomic, localization and dynamic analyses.

Because AAK1 possesses an atypical activation segment that confers constitutive catalytic competence (*1, 2*), its signalling output is most plausibly regulated by spatial and temporal recruitment. Rather, consistent with kinases of similar architecture (*3*), we propose that AAK1 activity in cells is governed by spatiotemporal proximity to substrates. This premise was corroborated by our live-cell TIRFM imaging that shows selective enrichment of AAK1 at mature, adhesions on collagen, with the AAK1 recruitment peaking at the transition into

disassembly (Fig. 6). In contrast AAK1 remains diffuse at the plasma membrane under FA-deficient attachment conditions (Fig 5B).

Regarding the functional consequences of PDLIM5 phosphorylation, Fig. 4B showed that the phosphomimetic mutant is associated with reduced steady-state FA density relative to WT. FRAP analyses further demonstrated that $PDLIM5^{T290D}$ exhibits faster molecular exchange at focal adhesions (Fig. 4C; Fig. S7A), consistent with shorter residence time. Independent TIRFM quantification reveals reduced steady-state levels of $PDLIM5^{T290D}$ at FA and along actin filaments (Fig. 4D). Together, these orthogonal readouts support a shift toward a more labile and less stably engaged PDLIM5 pool at FA upon phosphomimetic substitution at T290.

To further test functional consequences of phosphorylation at PDLIM T290 and Talin 1 T2270 we added the requested phospho-null mutant experiments (new Fig. 6D–E and in copy below). Both $PDLIM5^{T290A}$ and Talin-1^{T2270A} show prolonged late-phase retention during disassembly relative to their WT counterparts, consistent with phosphorylation promoting timely disengagement of the substrates. The effect is strongest for Talin-1^{T2270A}, which shows a delayed intensity decline (time to drop below 80% of peak: 71% → 85% of normalized FA lifetime), a marked increase in end-of-lifetime residual signal (~2% → ~23% of peak), and increased late-phase integrated signal (area under the curve, AUC(60–100): +38 %). $PDLIM5^{T290A}$ shows the same direction of effect but with smaller magnitude (e.g., time to drop below 80%: 68% → 72% of normalized lifetime).

Together with the AAK1 recruitment and localization data and the biochemically and structurally validated PDZ-mediated AAK1–PDLIM5 interaction (Fig. 7), these new experiments further strengthen our revised model. Specifically, they support a link between FA-proximal AAK1 recruitment and phosphorylation-dependent substrate disengagement during focal-adhesion turnover.

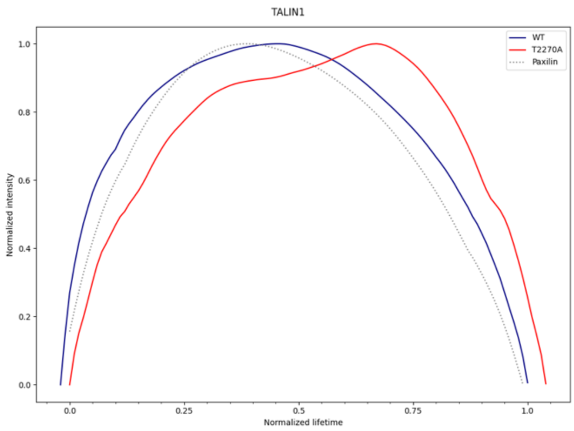

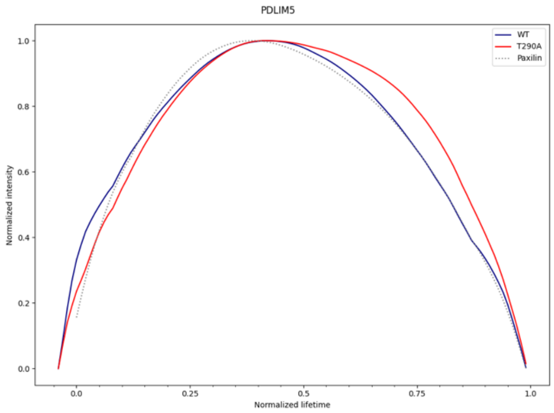

Major comment 2: Talin-1 localization in the cells is never shown in this manuscript. Imaging showing Talin-1, AAK1, and PDLIM5 co-localization at FAs would be needed.

Response: This experiment has been now carried out and images from triple colour live cell TIRFM imaging of AAK1 and TALIN1 has been included in Fig. 5D to demonstrate the colocalization of AAK1, TALIN1 and PDLIM5 in FA in live cells plated on collagen.

Major comment 3: Talin-1 should be added to the discussion with proper references. The identification of a phosphosite (T2270) in the Talin-1 R12 domain, and its possible functional consequences, as well as the contribution of other Talin-1 phosphorylating kinases, should be discussed. Here are some useful references.

Response: We have expanded the Discussion to (i) describe the known role of the Talin1 R12 domain in Talin-1 autoinhibition, (ii) discuss plausible functional consequences of the

newly identified R12 phosphosite T2270, and (iii) place our findings in the broader context of Talin-1regulation by other kinases, using the suggested references from the Reviewer.

Dedden et al. established an autoinhibited closed Talin1 conformation in which R12 forms a 'lid' over the FERM PIP2-binding surface (*4*). This autoinhibited conformation is supported by charge complementarity at the F2–R12 interface: F2 residues K272/K274 contact E2288/D2297 within an acidic R12 region that includes E2288, E2294, and D2297. Because Talin-1 F1-F2 domains contain flexible linker (*5*) and adopts a conformational ensemble, the F2 domain could potentially sample alternative geometries. The basic F2 surface could be brought into closer proximity to the negative charge of phosphorylated T2270, thereby shifting the equilibrium towards the autoinhibited state of Talin-1. Interestingly, T2270 is located next to other reported R12 phosphosites (PhosphoSitePlus database also lists phosphorylation on S2273 and T2281), raising the possibility that other phosphorylation events could further modulate the charge of this R12 helix. Consistent with this hypothesis, our new live-cell analysis shows that preventing phosphorylation at T2270 increases Talin-1 retention within focal adhesions. This suggests that phosphorylation could potentially shift conformation and Talin-1 functional engagement with the plasma membrane, integrins, and the actin cytoskeleton, ultimately influencing focal adhesion dynamics.

Major comment 4: On page 6, lines 156-159, it is reported that PDLIM5 and Talin-1, in addition to AP2 μ T156, are the principal substrates of AAK1. The other identified phosphorylated peptides are, however, not shown, making it difficult to judge this result.

Response: We thank the Reviewer for this helpful comment. We agree that the remaining PRM-tested phosphopeptides should also be shown. We have now added a new Supplementary Figure S3 showing the quantitative PRM results for the remaining PRM-detected phosphopeptides (presented in the same format as Fig. 1J–K), together with the corresponding statistical analysis.

Major comment 5: It is reported on page 6 that upon genetic or pharmacological perturbation, PDLIM5 and Talin-1 levels remained constant (Figure S3), but in Figure 2C, Talin-1 is upregulated. Adding statistical analysis in Figure S3 might show that Talin-1 is upregulated in these samples as well. However, if the data is not coherent, it should still be commented.

Response: We thank the reviewer for this careful analysis. We corrected an error in the text description to ensure it accurately reflects the data, the data are internally consistent. Figures 2C and S4 show that the Talin1 is upregulated upon AAK1/BMP2K deletion. The statistical analysis in Fig. S4 shows a reproducible increase in Talin1 abundance by 16%. This statement has now been corrected in the text and statistical analysis has now been added in Fig. S4.

Major comment 6: The overlap of the data in genetic and pharmacological perturbations, or the lack of it, should be reported when presenting the results of Figure 2. Specifically, the presence of PDLIM5 and Talin-1 only in one of the panels should be discussed.

Response: We thank the Reviewer for suggesting this. We carried out this analysis and it further supports our original conclusions, and we now include overlap metrics in the revised SI for overlap at both the protein and pathway levels.

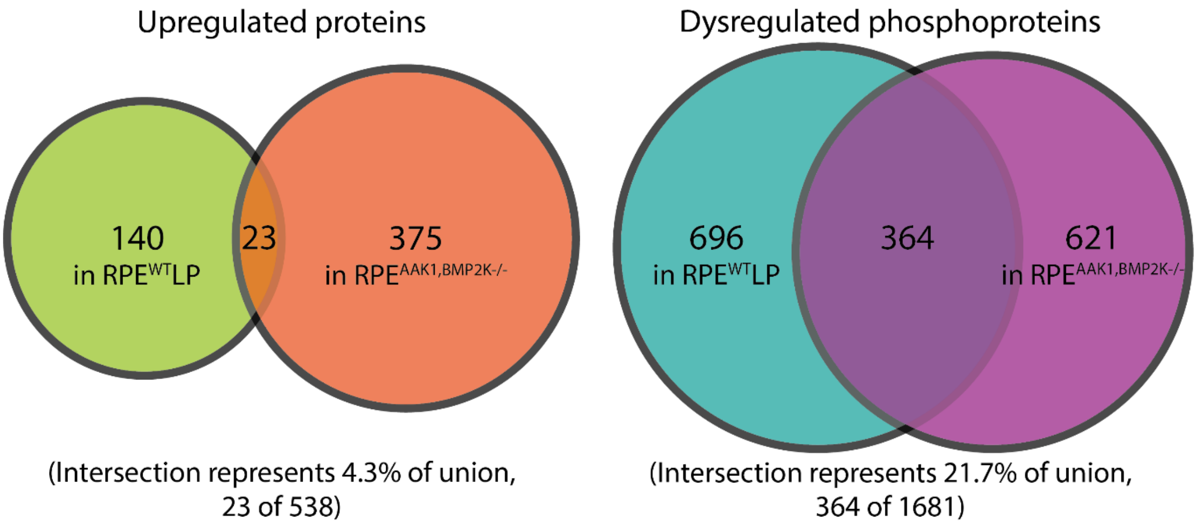

New SI Figure 6: Protein-level intersection between pharmacological inhibition and genetic loss of AAK1/BMP2K.

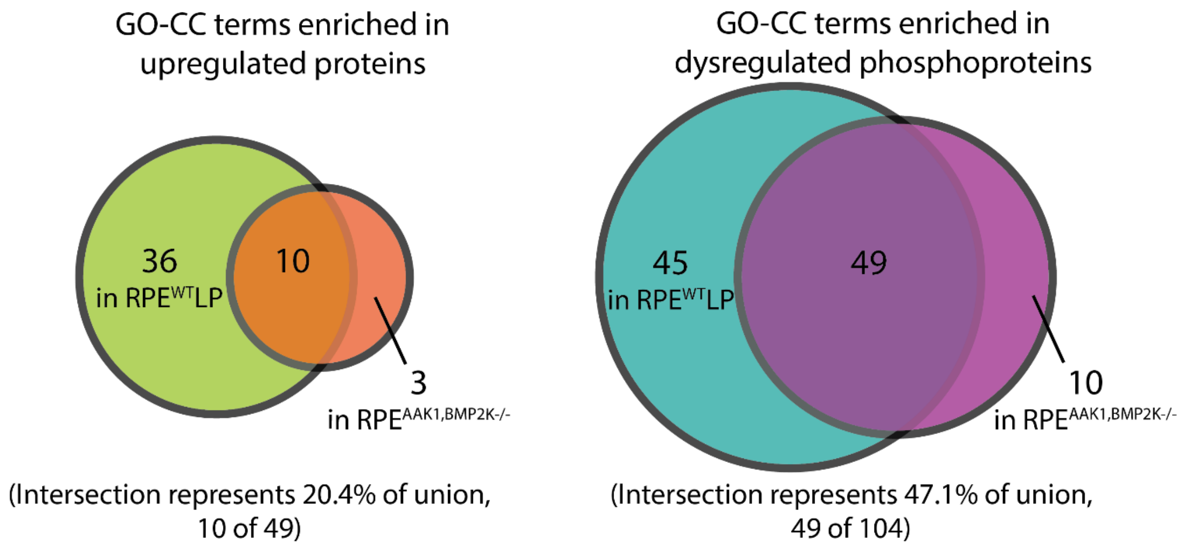

New SI Figure S6: Pathway-level intersection of GO Cellular Component enrichment.

The intersection is very robust on the phosphoprotein level. This is consistent with a kinase-centered mechanism. ~364 phosphoproteins are significantly dysregulated in both the acute inhibitor condition and the knockout (≈22 % intersection of the union), compared with ≈4 % intersection among upregulated proteins. This indicates that both perturbations consistently affect a shared set of phospho-regulated proteins. Moreover, the phosphoproteins at the intersection are enriched for focal adhesion/cytoskeletal components, as visualised by the shared GO-terms (see new Fig. S6 in the manuscript). Using GO-CC enrichment from the corresponding hit lists, we observe substantial intersection: 10 shared GO-CC terms (~20% of 49 total) for the upregulated proteome and 49 shared GO-CC terms (~47% of 104 total) for the dysregulated phosphoproteome.

Regarding the reviewer's specific point: PDLIM5 and Talin1 were detected in all datasets. However, they appear in only one Fig. 2 panel because they did not meet the significance and thresholds for explicit presentation in the other panels. The rationale for selecting thresholds is explained in detail in the section Minor comments – Fig. 2 (Page 18 of this document).

Major comment 7: The specificity of the inhibitors should be explained. E.g., LP-935509 is used as an AAK1 and BMP2K inhibitor (Figure 2) or an AAK1 inhibitor (Figure 3). In the referred study, it is described as a NAK family inhibitor.

Response: We thank the reviewer for pointing this out and we clarified the specificity of the two inhibitors (LP-935509 and LX-9211) in the text. Below, we provide information for the reviewer how the specificity of the two inhibitors used in this study is defined in the original literature. LP-935509 (an acyclic pyrazolopyrimidine) and LX-9211 (a distinct chemotype bi(hetero)aryl ether) represent two different chemical structures. In the revised manuscript, we have now also emphasized why both inhibitors were employed in parallel in the same assay (Fig. 3E). By showing that two independent inhibitors with different chemical structures and selectivity profiles produce similar outcomes, we address potential skepticism about off-target effects of these inhibitors.

LP-935509 (*Kostich et al., J. Pharmacol. Exp. Ther. 2016*)
AAK1 IC_{50} = 3.3 nM
BMP2K IC_{50} = 14 nM
GAK IC_{50} = 320 nM (hundred-fold weaker potency than for AAK1)
These IC_{50} values explain why, in our previous study and in others (e.g., Mensing et al., *Eur. J. Med. Chem.*, 2025), LP-935509 has been described as a NAK family inhibitor with strong preference for AAK1 and BMP2K

LX-9211 (*Luo et al., J. Med. Chem. 2022*)
AAK1 IC_{50} = 2 nM
BMP2K IC_{50} = 17 nM
GAK IC_{50} = no measurable inhibition of GAK at concentrations up to 1 µM, kinome-wide screening has shown is highly selective, with minimal off-target kinase activity up-to 1 µM for LX-9211

Major comment 8: The cell lines SH-SY5Y and MDA-MB-231 should be introduced and references added. In this study, conclusions are drawn from these cell lines that AAK1-dependent kinase-driven motility is not limited to epithelial cells (page 8, line 223-226). However, MDA-MB-231 has an epithelial origin, and ATCC describes the morphology as epithelial, even though these cells have mesenchymal characteristics. Overall, the identity of these cell lines should be clarified better.

Response: We thank the reviewers for their comment regarding the identity and origin of our cell lines used in the experiment depicted in Fig. 3E. We included a more accurate description of the cell lines and relevant references in the text: SH-SY5Y as a neuroblastoma (non-epithelial) and MDA-MB-231 as an epithelial-derived line with mesenchymal/invasive phenotype. MDA-MB-231 are e-cadherin negative and therefore cannot form e-cadherin mediated junctions. By adding these cell lines into our panel in Fig.3E, we aimed to strengthen our conclusions on AAK1-driven migration across different lineage backgrounds.

Major comment 9: On page 12, rows 314-315, it is nicely described that the cells were cultivated on collagen to recover FA components. How were the cells cultured in other assays, since they appear to form FAs? This information should be included.

Response: We thank the reviewers for pointing out this omission in the Materials and Methods section. We have now revised the Methods to include full details of the coating procedures used throughout the study.

Assays requiring rapid, robust FA formation were performed on collagen-coated surfaces unless stated otherwise. Specifically, for live-cell TIRFM imaging (FA lifetime tracking, AAK1/BMP2K recruitment profiling, and lifetime-aligned analyses), FRAP at adhesions, fixed-cell adhesion/cytoskeleton localization, and migration assays, cells were plated on rat tail collagen I (Gibco A10483-01; 100 µg/mL in 20 mM acetic acid; incubated at 37 °C for 30 min) to promote rapid and robust FA formation.

For experiments in which AAK1 localization and the AAK1 interactome were tested across distinct attachment contexts, we compared collagen to poly-L-lysine (PLL), which supports integrin-independent, nonspecific electrostatic attachment and is FA-deficient. PLL solution (Sigma P8920, MW 150,000–300,000 g/mol a 0.1% w/v stock in H_2O) was diluted to a 0.01% w/V working solution in deionized water for coating. Apo-transferrin (Sigma-Aldrich T5391) was used also as a FA-negative control coating at 0.1 mg/mL where indicated.

For plate-based receptor internalization assays using in-cell ELISA format requiring extensive wash steps, cells were seeded in gelatin-coated 96 well plates overnight to ensure uniform attachment compatible with assay handling and to minimize non-specific antibody adsorption to the plate.

Proteomics experiments were performed under adherent culture conditions matched to the relevant assay context. Targeted PRM and global proteome/phosphoproteome profiling were carried out with cells plated on rat tail collagen I. When FA presence/absence was the variable of interest (e.g., collagen vs PLL interactomics in Fig. 5), samples were prepared under matched collagen and PLL conditions, analogous to the corresponding TIRFM experiments, to identify adhesion-dependent differences in AAK1 localization and/or interactome.

Major comment 10: PDLIM5 and Talin-1 were not found as AAK1 interactors in Figure 5G. They are, however, present in the MS result lists with lower intensity ratios. This should be commented on, and possible explanations discussed. In addition, it should be discussed that in co-IP experiments, the cells are lysed, which might lead to the interaction of proteins normally separated in different compartments.

We thank the reviewer for raising this point and for carefully examining our supplementary MS data (Data S2). In our co-IP/MS experiments, PDLIM5 and Talin-1 were indeed detected. However, they are not shown in Fig. 5G because that panel is a GO/STRING network visualization of AAK1 interactors filtered by stringent enrichment criteria ($p \leq 0.05$ and $log_2FC > 0.3$). Neither PDLIM5 nor Talin-1 met these thresholds, as expected given that kinase-substrate interactions are transient and sub-stoichiometric. We have now clarified this in the text of the manuscript. The intent behind this experiment was to understand how ECM/integrin engagement rewires the AAK1 interactome.

We very much agree with the reviewer that co-IP experiments that are performed after cell lysis can either disrupt low-affinity interactions and/or allow post-lysis associations between components that may not interact in intact cells. To address this inherent limitation of co-IP experiments, our conclusions do not rely on co-IP/MS alone. Instead, we integrate multiple orthogonal approaches. Substrate identity and targeting are supported by:

- Cellular phosphopeptide evidence: PRM-LC-MS shows that phosphorylation of PDLIM5 T290 and Talin-1 T2270 decreases reproducibly by >50% in AAK1/BMP2K double knockout cells, identifying them as principal cellular substrates (Fig. 1J and K) which imply (in combination with in-vitro data) interaction between the substrate and the kinase, albeit transient and substochiometric.

- Evidence from live-cell TIRFM imaging**:** AAK1 is selectively recruited to mature paxillin-positive focal adhesions on collagen, where PDLIM5 and Talin1 are also present (Fig. 5 and Fig.6). AAK1 remains diffuse at the plasma membrane in cells on PLL coating where FAs do not form (Fig. 5B).
- Direct molecular and structural evidence**:** an AAK1 C-terminal peptide pull-down identifies PDZ-containing PDLIM proteins including PDLIM5, and we further validate direct binding structurally and biophysically (Fig. 7B, C, and D, Fig. S10). In the manuscript, we emphasize that the interaction between AAK1 and PDLIM5 is weak and transient (Fig. S10A). In our model this is compatible with catalytic turnover and repeated phosphorylation of PDLIM5 when it is associated in multiple copies with α-actinin on actin filaments.

Major comment 11: The model in Figure 7F involves Talin-1, even though the data supporting this is insufficient. Talin-1 should also be depicted as a dimer in a stretched form in mature FAs.

Response: We thank the reviewer for this important point. We agree that our original schematic was not supported with sufficient data on Talin-1 phosphorylation. In the revised submission, we have strengthened the experimental evidence as discussed above and adjusted Figure 7F accordingly. We implemented the reviewer's suggestion regarding Talin-1 representation. In Fig. 7F Talin-1 is now depicted as autoinhibited monomer in the cytosol based on the full-length structural model by Dedden et al (*4*), and as an extended dimer in focal adhesions.

We recognize that the downstream mechanistic consequences of Talin-1 T2270 phosphorylation (i.e., the structural consequences and/or molecular partners affected) remain to be resolved on a molecular level. We therefore revised Fig. 7F to present Talin1 phosphorylation as a proposed regulatory input rather than a fully defined pathway (dashed arrow and softened wording), consistent with the level of mechanistic resolution currently supported by the data.

Major comment 12: Figure S1 has several inaccuracies that should be clarified: the number of lanes in Ponceau control does not match the immunoblot, unclear which Ponceau panel is

corresponding to which immunoblot in C and D, the b tubulin lane is broken in E, and the figure legend appears to be swapped between E and F, and in G there is only one panel (not right and left). Information is missing about the GAPDH and b-tubulin (Table S7), the Ponceau S staining, how the membranes were stained, and what was used to capture the images, and information on how/if the data was quantified and/or normalized to loading controls, and the number of replicas. The panel H should also include the WT sample.

Response: We are grateful to the reviewer for highlighting the need for more clarity in figure S1 and providing a thorough breakdown of the issues observed. Figure S1 has been reworked using this feedback to correct mistakes and make it clearer for the reader and Source file was submitted. The reviewer stated the Ponceau S loading controls needed correcting and reorganising to make it clear which Ponceau S loading control belonged to which immunoblot. To address this, all Ponceau S loading controls have been reorganised and placed adjacent to their corresponding blot for easy comparison. All Ponceau S loading controls have been checked and corrected where needed to ensure they match the blots shown in each panel. We agree with the reviewer that immunoblot FigS1F should include $RPE^{AAK1-/-}$ mNG-Paxillin sample to support the FRAP assay (Fig. 3H), and FigS1H (now FigS1G) needs to include RPE^{WT} sample to show comparison of endogenous AAK1 to the AAK1 rescue cell lines. These blots have been redone to include these samples (Fig. S1F, S1G). The reviewer highlighted inaccuracies in the figure legend, which have been addressed. The reviewer also stated that the immunoblot in FigS1E has a break in the b-tubulin loading control, and that information about GAPDH and b-tubulin are missing from Table S7. For this, immunoblot FigS1E (now FigS1H) has been redone, utilising HSP90 loading control in the same range of molecular weights. Information regarding GAPDH and HSP90 has been added to Table S7. Lastly, we have updated the materials and methods to include relevant information about the immunoblotting procedure, including Ponceau S staining, immunoblot acquisition, data quantification, replicates and normalisation to loading controls. All uncropped immunoblots, replicates, loading controls and protein expression analyses (rescue cell lines and siRNA knockdown efficiency) have been included in the source file.

Major comment 11: The accuracy of data presentation in the text and figures should be improved (listed in the minor comments).

Response: We sincerely thank the reviewers for identifying these issues. We have addressed

all items noted in the Minor comments and revised the text and figures accordingly to improve the accuracy and clarity of the data presentation.

Minor comments and responses

a) Abbreviations such as PM and CCP are not consistently used or spelled out when introduced. **Response:** We apologise for this oversight, this has now been corrected.
b) On page 3, row 100, BMP2K exhibits prominent concentration within CCPs at the PM, rather than complete concentration. **Response:** This has been now corrected to prominent concentration.
c) On page 6, rows 156-159, the sentence is complex. **Response:** The sentence has been broken down for clarity.
d) On page 6, row 163. Based on the results presented in this section, the sentence "The similar sensitivity of both PDLIM5 and Talin1 suggests that AAK1 or BMP2K may regulate mechanosensitive adhesion module, extending their signalling beyond endocytosis." is too far-reaching. **Response:** We agree that with the data presented up to this section that this statement may be too definitive. We have altered the text to reflect this: "The similar sensitivity of both PDLIM5 and Talin1 suggests a potential yet undefined role for AAK1 or BMP2K in the adhesome, extending outside their known role of signalling in endocytosis."
e) On page 7, row 175, a reference to the dataset is missing, or where can this dataset be found? **Response:** Thank you for spotting this, this information has been now added in the text, the dataset was part of the file Data S2.
f) On page 8, row 214-216, the sentence (In contrast, the identification of PDLIM5 and Talin1 as novel substrates, together with the broad distribution of AAK1 across the plasma membrane, strongly suggests a previously unrecognized role for AAK1 in FA maintenance during cell migration) is illogical and appears abruptly at this point in the manuscript.
Response: We agree that this sentence was abrupt and over-interpreted the data at that stage. We rewrote it to present the substrate identification as motivation for the subsequent FA-localization and functional experiments and removed the causal language ('strongly suggests', 'FA maintenance'). The sentence has been rewritten to: "Together, these data indicate that BMP2K, rather than AAK1, is the primary NAK supporting clathrin-mediated endocytosis in RPE cells. However, identification of the adhesome proteins PDLIM5 and Talin1 as AAK1/BMP2K-responsive substrates, together with AAK1 broader PM distribution, prompted us to test whether AAK1 also contributes to adhesion-dependent processes during cell migration."

g) On page 9, number 3 is missing from the reference Fig. 3F, G. **Response:** This has been edited in the text.

h) The data on page 11, rows 283-290, should be better presented and explained. This also applies to the corresponding figure legend and to the materials and methods. Now it is unclear if this is a co-IP of PDLIM5 in WT and AAK1-/- cells. **Response**: The text has been edited for clarity.

i) On page 11, a reference to Fig. 4D is missing. Line 277**. Response**: The reference has been added.

j) On page 12, row 314, a reference is missing for the data "Co-immunoprecipitation of endogenous AAK1 from collagen-adherent RPE cells under steady-state conditions recovered multiple FA components, with α-actinin-4 being the most enriched". **Response:** The reference has been added.

k) The AAK1 splice variants are presented first on page 17, row 404. The splice variant information should be included in Figure 1A. **Response:** The legend of Figure 1A has been edited to clearly state that the presented splice variant is the canonical one with accession numbers and references to Uniprot and Ensembl databases.

l) The data in Figure 7C should be better explained on page 17, rows 410-412. It should be mentioned that this is a co-IP experiment in WT and AAK1-/- cells. **Response:** The sentence has been rewritten for clarity and this information explicitly stated.

m) On page 17, row 429, a reference to Figure 7E is missing. **Response:** The reference has been added.

n) On page 30, rows 819-20, the authors state that genetic modifications were verified by RT-PCR and WB, but no RT-PCR data are shown in the manuscript. **Response:** We apologise for this omission. The decrease in mRNA in the single knockout cell lines has been validated using RT-qPCR and this data has now been included in Fig. S11.

o) On page 31, row 870, this data is missing: "the population with the AAK1 expression closest to that of RPE^{WT} cell lines was selected for further experiments." **Response:** We added reference to Fig. S1G and the associated source data (western blot quantification) for cell line characterization.

p) On page 31, rows 870-871, the authors state that a population of mScarlet-AAK1-WT and mScarlet-AAK1-GGGG cell lines were selected based on having expression closest to RPE-WT levels. However, no comparison of the expression to RPE-WT cells is included in Figure

S1H. **Response:** We have included the immunoblot in Fig. S1H that shows RPE^{WT} cells for direct comparison of AAK1 expression levels.

q) The Materials and methods section includes many redundant titles (e.g. MS and data analysis), which hamper the clarity of this section. **Response:** The sub-section headers of the materials and methods relating to mass spectrometry experiments have been deleted.

<u>Figure comments - responses</u>

<u>FIG. 1</u>

Figure 1A: The source of the figure or data is missing, and the abbreviations (such as SLiMS) and colors are not explained. **Response:** We apologise for this omission. The figure legend has been updated to clarify the abbreviation "SLiMs" as short linear motifs. Identified SLiMs are depicted as coloured lines in the c-terminal tail of the kinases. The legend of SLiMs in the figure specifies the short linear motif identity based on its colour. In the legend we provide references to the UniProt accession numbers of which the canonical splice variants have been used, papers from where the cartoon is adapted, as well reference to the ELM database for short linear motifs have been included in the figure legend.

Figure 1B and C: The zoomed areas are not marked, and a scale bar is missing from the upper panel. Why are the morphologies of the mScarlet-BMP2K and mScarlet-AAK1 cells very different? **Response:** White boxes have been added to the upper panels to indicate the zoomed in area. A scale bar has been added to the upper panel. RPE cells progressively form multicellular clusters after seeding. Presenting multiple cells in a single field of view highlights the robustness of the phenotype across multiple cells and reduces the risk of selection bias. However, it prevents reliable delineation of individual cell boundaries in the TIRFM images. Consequently, the apparent morphology of these cell clusters varies with cluster size. Here the mScarlet-BMP2K image has 4 cells clustered, whereas mScarlet-AAK1 has only 2 cells more spread out.

Figure 1H: Nomenclature for the positive control is not coherent with the main text (AP2 μ-subunit peptide containing residue T156 (ITSQVTG) vs. AP2M1). Why is the data normalized to PDLIM5 in H and I and not to the positive control? This should be explained. Why is there an arrow between panels I and J? **Response:** We thank the reviewer for pointing out these issues. The nomenclature has been corrected throughout the figure and text to use AP2μ, ensuring consistency with the main manuscript. To avoid ambiguity and reduce visual clutter, the arrow has now been removed. The AP2μ T156 peptide was included as a known

positive control to confirm expected kinase activity and motif recognition. For Fig. 1H–I, initial rates were normalized to PDLIM5 because it was a robustly phosphorylated new candidate peptide across the peptide panel and conditions used in this assay, providing a practical internal reference that preserves dynamic range for weaker candidates.

FIG. 2

Figure 2: Why are different Log2 fold changes used for different panels? This should be explained. **Response:** Biologically, an acute 6-hour inhibition transiently blocks AAK1/BMP2K catalytic activity (allowing rapid but mild, transient network adaptations), whereas a chronic genetic knockout removes the kinase proteins entirely (eliminating both catalytic and scaffold functions) and thus allows sustained, longer-term network reprogramming (*6, 7*). Hence chemical and genetic perturbations are expected to yield different magnitude of changes that are reflected here by use of different logFC thresholds to visualize the data. For panels A and B, a lower threshold of logFC 0.3 and 0.5 respectively was chosen, as the pharmacological perturbation results in fewer, smaller changes on both proteome and phosphoproteome level. On the other hand, a knockout leads to bigger changes on both proteome and phosphoproteome level, due to long-term adaptation and broader proteome changes, so we chose a more stringent cutoff of logFC 0.5 and 1 for the proteome and phosphoproteome analysis respectively.

FIG. 3

Figure 3B: Statistical comparison between RPE WT and RPE BMP2K rescue is missing. **Response:** Statistical comparison between WT and BMP2K rescue has been added.
Figure 3C: Lacks a unit on the y-axis. **Response:** A.U. has been added as units to Fig 3C and all other figures where this was missing.
Figure 3D: Statistical comparison to the positive control is missing**. Response:** Statistical comparison between WT and positive control has been added.
Figure 3E: Contains data for RPE, but RPE is not mentioned in the figure legend. The title for the y-axis is missing. **Response:** Title has been added to the y-axis. Figure legend has been updated to include mention of RPE for 3E.
Figure 3F: It is unclear how FA lifetimes were assessed. With paxillin or something else? It should be explained in the figure legend**. Response:** We have added to figure legend that FA lifetimes were assessed by segmentation of mNeonGreen-paxillin signal in our time-lapse movies.

Figure 3H (left plot): lacks a unit on the y-axis. As a supporting material for Figure 3H, Figure S1F should contain RPE AAK-/- sample as well.

Response: A.U. has been added as units to Fig 3H and all other figures where this unit was missing. Immunoblot for Fig S1F has been redone to include $RPE^{AAK1-/-}$ mNeonGreen-Paxillin.

FIG. 4

Figure 4A: The schematic illustration (upper panel) is not explained in the figure legend.
Response: This has been added to the figure legend.
Figure 4B: It is unclear how FA lifetimes were assessed. With paxillin or something else? It should be explained in the figure legend. **Response**: We have added to figure legend that FA lifetimes were assessed by segmenting mNeonGreen-paxillin in our time-lapse movies.
Figure 4C: An image showing PDLIM5T290D-HaloTag distribution should be included in the upper panel. **Response:** We thank the reviewer for this suggestion. The upper panel of Fig. 4C is included to illustrate the FRAP acquisition strategy (bleach ROI, reference ROI, background ROI) and was acquired by confocal imaging under settings optimized for photobleaching/recovery measurements, rather than for visualizing global differences in PDLIM5 recruitment. Under these conditions, the reduced FA association of the $PDLIM5^{T290D}$ mutant is not readily apparent by eye from a single confocal snapshot without the accompanying quantitative and kinetic FRAP analysis. The recruitment/localization phenotype of $PDLIM5^{T290D}$ is already directly visualized and quantified in Fig. 4D, where TIRFM shows markedly reduced $PDLIM5^{T290D}$ signal at FA and along actin filaments compared with $PDLIM5^{WT}$.
Figure 4D: Scale bars and y-axis values and units are missing. Should it be written Paxillin instead of Phalloidin in the left panel? The images are unclear, and it is hard to tell what these structures are. The colors in the images are not explained**. Response**: We thank the reviewer for spotting this mistake. The left panel "Focal adhesions" should be labelled Paxillin instead of Phalloidin. This has been corrected. The y-axis has been updated to include units (A.U.) and scale bars have been added to the images. The figure has been reorganised, including labels of normalized fluorescence intensity with scale and units to provide more clarity to the reader and clarify the colours. The image labels have been made clearer to clarify the structures in each image.

Figure 4E, a title is missing. **Response:** Title "AAK1 dependent remodelling of PDLIM5 interactome" was added to the figure.

FIG. 5

Figure 5: Why is log2 fold change > 1 used here, when in previous analyses > 0.3 was used? **Response:** We used different $\log_2$ fold-change thresholds because these panels represent different types of interactomics comparisons with very different expected effect sizes and background levels.

In Fig. 5A, the goal is to define high-confidence AAK1 interactors by comparing an endogenous AAK1 co-IP in RPE^{WT} to a matched negative control in $RPE^{AAK1-/-}$ (i.e., an IP performed in the complete absence of the bait). The $RPE^{AAK1-/-}$ control captures proteins that bind non-specifically to the beads and/or the anti-AAK1 antibody, so true AAK1-dependent interactors are expected to show large enrichments over this "background-only" condition. For this reason, we applied a more stringent visualization cutoff ($\log_2FC > 1$, i.e., >2-fold enrichment) to ensure that the GO/STRING summary focuses on the most robust and reproducible AAK1-centered network.

By contrast, the analyses where we used $\log_2FC > 0.3$ (e.g., Fig. 4E, PDLIM5 co-IP: RPE^{WT} vs $RPE^{AAK1-/-}$; and Fig. 5G, AAK1 co-IP in RPE^{WT} cells plated on collagen vs PLL) are differential interactomics comparisons between closely related biological states**.** In these settings, the condition is not compared to a bait free control but directly to a "near-neighbor condition", so biologically meaningful changes in interactome composition are expected to be more modest in magnitude. Accordingly, we used a lower threshold **(**$\log_2FC > 0.3$).

Importantly, these $\log_2FC$ thresholds were applied only for GO/STRING visualization and summary plots; the underlying statistical analysis is unchanged, and the complete tables of interactors (including proteins below the visualization thresholds) are provided in the Supporting Datasets (Source file). We have also clarified this rationale in the relevant Methods/figure legend text to avoid confusion.

FIG. 6

Figure 6A: scale bar is missing. And **Figure 6B**: Information about the time is missing**.** **Response:** Scale bar and time stamps were added.

FIG. 7

Figure 7A: Isoform misspelled on the first row. Is the LIDL sequence specific for only these isoforms? **Response:** Thank you - the spelling error has been corrected. The LIDL sequence is not specific to only the isoforms shown but seen in multiple isoforms for many species. For example, the LIDL motif is found in isoforms X10, X11, X12, X13, X15 of Danio Rerio. The selected example isoforms reflect the sequences shown in Fig 7A, and are used to illustrate the evolutionary conservation of the LIDL motif in jawed vertebrates.

Figure 7 D: The figure legend contains (D) twice. **Response:** We removed the duplicate '(D)' from the Fig. 7 legend.

Figure 7 E: Missing relevant statistical comparisons, especially RPE-WT to RPE AAK1-rescue, to evaluate the effect of RPE AAK1-GGGG. The authors may want to refer to this figure on page 17, line 29, instead of referring only to S1H. **Response:** We added the statistical comparison to the panel 7E and we also added reference to the right location.

Figure 7 F: Is Talin1 phosphorylation at this site known for FA disassembly? The presented data does not support that. It should be added which parts are hypothetical. **Response:** We agree with the Reviewer that the original manuscript did not directly test whether Talin1 phosphorylation at T2270 contributes to FA disassembly. To address the role of Talin-1 T2270 phosphorylation, we have added new live-cell TIRFM experiments showing that the phospho-null Talin1 mutant (T2270A) exhibits prolonged residence at FAs compared with WT Talin1 in lifetime-aligned intensity profiles (Fig. 6E). This phenotype is consistent with the interpretation that phosphorylation at T2270 promotes timely Talin1 disengagement during the disassembly phase. We have therefore revised the Discussion and the schematic model to make explicit that the observed change is in Talin1 FA dynamics and the proposed structural/conformational explanation (informed by Dedden et al.(*4*)) remains a working hypothesis and not a demonstrated mechanism.

Figure S6E*:* Lacks statistical analysis. **Response:** Statistical analysis was added.

Reviewer #3 (Remarks to the Author):

I co-reviewed this manuscript with one of the reviewers who provided the listed reports. This

is part of the Nature Communications initiative to facilitate training in peer review and to provide appropriate recognition for Early Career Researchers who co-review manuscripts.

Response: We thank the Reviewer for the many helpful comments and suggestions. We have revised the manuscript accordingly and hope the changes satisfactorily address the points raised.

References:

1. F. J. Sorrell, M. Szklarz, K. R. Abdul Azeez, J. M. Elkins, S. Knapp, Family-wide Structural Analysis of Human Numb-Associated Protein Kinases. *Structure* **24**, 401-411 (2016).
2. J. Eswaran *et al.*, Structure of the human protein kinase MPSK1 reveals an atypical activation loop architecture. *Structure* **16**, 115-124 (2008).
3. L. J. Fulcher, G. P. Sapkota, Functions and regulation of the serine/threonine protein kinase CK1 family: moving beyond promiscuity. *Biochem J* **477**, 4603-4621 (2020).
4. D. Dedden *et al.*, The Architecture of Talin1 Reveals an Autoinhibition Mechanism. *Cell* **179**, 120-131 e113 (2019).
5. P. R. Elliott *et al.*, The Structure of the talin head reveals a novel extended conformation of the FERM domain. *Structure* **18**, 1289-1299 (2010).
6. Z. A. Knight, K. M. Shokat, Chemical genetics: where genetics and pharmacology meet. *Cell* **128**, 425-430 (2007).
7. J. E. Kung, N. Jura, Structural Basis for the Non-catalytic Functions of Protein Kinases. *Structure* **24**, 7-24 (2016).

Reviewer comments

Reviewer #1 (Remarks to the Author):
The authors did adequately respond to my criticisms. The paper is in my view now ready for publications. I apologise for not responding quicker

Reviewer #2 (Remarks to the Author):
I have reviewed the revised manuscript by Krocianova et al. and find that the authors have addressed the concerns raised in the previous review very thoroughly. The revisions have substantially improved the manuscript and more convincingly support the conclusions drawn. I recommend one minor improvement: please add x and y axis titles to Figures S4A–D, S8E, and S11A–B to ensure full clarity. Addressing these final details will, in my opinion, make the manuscript suitable for publication.

Reviewer #3 (Remarks to the Author):
I co-reviewed this manuscript with one of the reviewers who provided the listed reports. This is part of the Nature Communications initiative to facilitate training in peer review and to provide appropriate recognition for Early Career Researchers who co-review manuscripts.

Response:

Dear Reviewers,

We thank you for your time and positive assessment of the revised manuscript. We confirm that figures S4A–D, S8E, and S11A–B include axis labels. We hope the manuscript is now suitable for publication.
